# Supplementary material for: A viral-based individualized neoantigen vaccine as adjuvant treatment in resected head and neck squamous cell carcinoma: a randomized Phase I trial
Source: Nat Commun. 2026 Aug 17;17:9863. doi: 10.1038/s41467-026-76667-1 (PMC13578212; doi:10.1038/s41467-026-76667-1)
Supplement: Supplementary file 1 [file 41467_2026_76667_MOESM1_ESM.pdf]

Supplementary Information

Ottensmeier et al, A Viral-based individualized neoantigen vaccine as adjuvant treatment in resected head and neck squamous cell carcinoma: a randomized Phase I trial

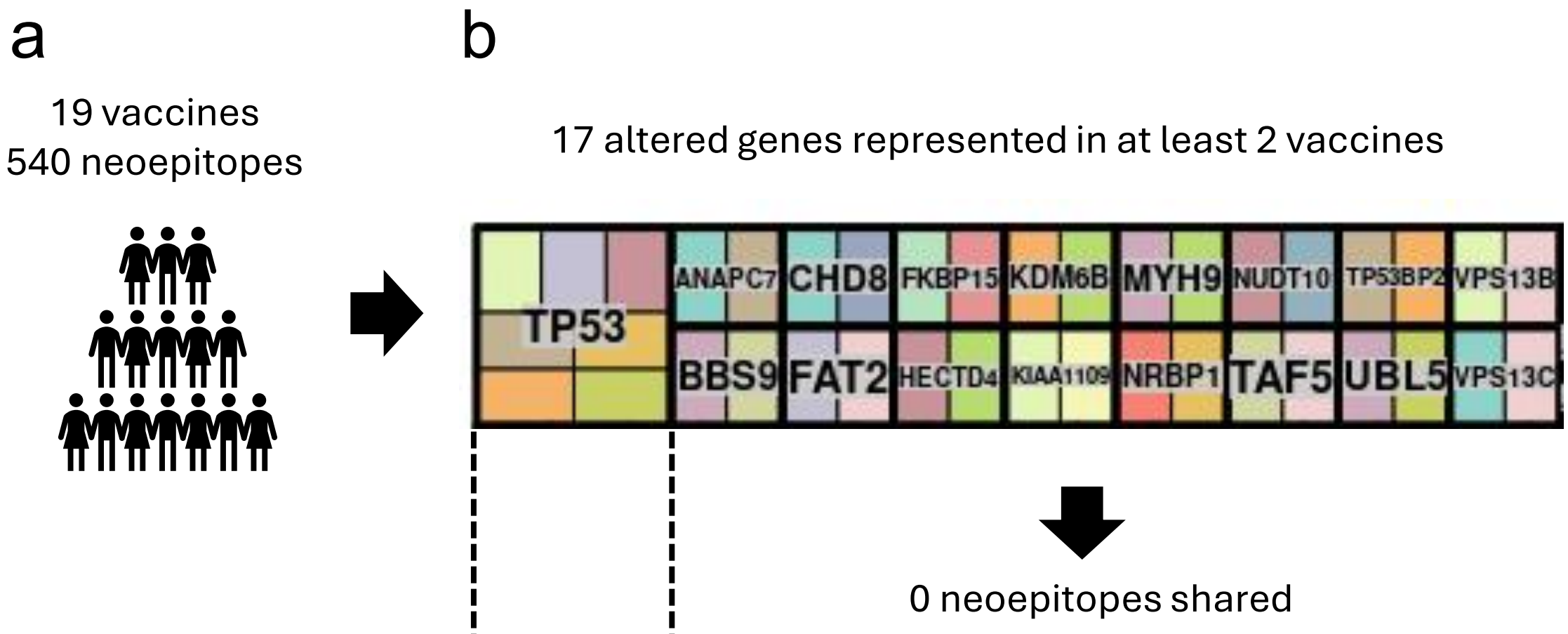

c

Patient ID	Mutation	HLA restriction	Vaccine neoantigen ID
Pt HN03	17_7673766_E/V	B*07:02	11
Pt HN07	17_7675161_P/T	B*07:02	16
Pt HN08	17_7673787_P/R	B*18:01	9
Pt HN09	17_7674950_L/R	B*07:02	2
	17_7674220_R/Q	B*07:02	17
Pt HN16	17_7674230_G/S	A*31:01	13
Pt HN10	17_7674216_R/S	A*02:01	10

Supplementary Figure 1. Conservation of vaccine neoantigens. a, Total number of vaccine neoantigens in patients treated (Arm A or B). **b**, Mutated genes selected as vaccine neoantigen in more than one vaccine. The number of squares for each gene indicates the number of vaccines containing the gene. The mutation is not necessarily the same for each vaccine. **c**, TP53 vaccine antigens included in each vaccine. All vaccine neoantigens are different because they are based on different mutations.

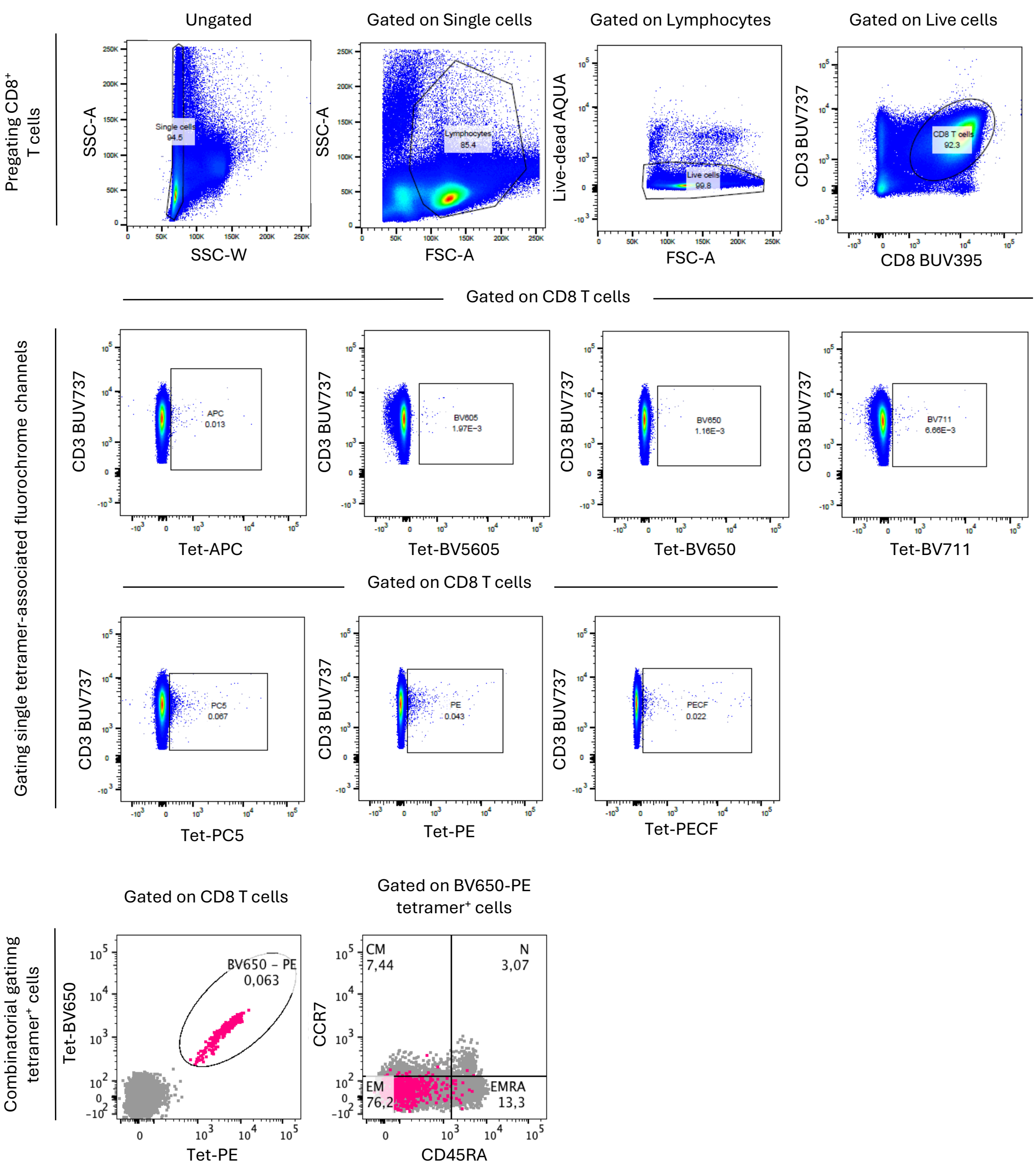

Supplementary Figure 2. Neoantigen-specific CD8⁺ T cell gating strategy. Tetramer⁺ cells were determined by pregating of CD8⁺ T cells followed by combinatorial gating of channels associated with tetramer fluorochromes (APC, BV605, BV650, BV711, PC5, PE and PECF). Positive gates in the single tetramer fluorochrome channels were determined for each patient based on the baseline sample and applied to all other samples for this patient. Following combinatorial gating, tetramer⁺ populations were determined as distinct populations double positive for the 2 tetramer fluorochromes. Frequency of Tn, Tcm, Tem and Temra was assessed based on CCR7 and CD45RA expression in the tetramer⁺ cells.

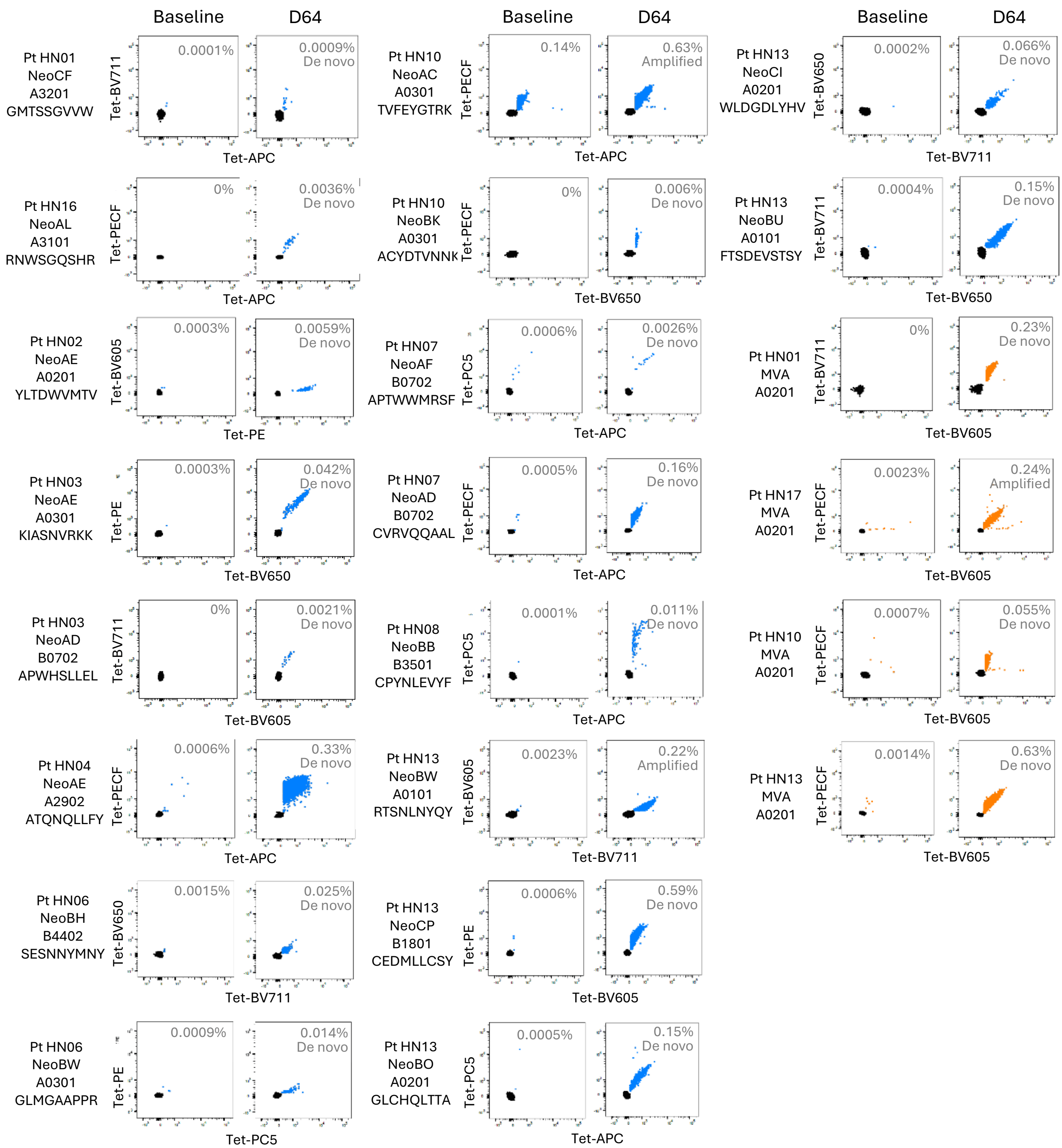

Supplementary Figure 3. Antigen-specific CD8⁺ T cell responses detected by tetramer at baseline vs D64. For each tetramer response to treatment reported in Supplementary Table 3, the overlay of the combinatorially-gated double positive tetramer population (within CD8⁺ T cells) and the CD8⁺ T cell subset negative for all tetramer-associated fluorochromes is shown. The frequency of the tetramer⁺ population within CD8⁺ T cells is indicated in grey. Responses to treatment were categorized as *de novo* if not detected at baseline (less than 10 tetramer⁺ cells) but detected at D64 and amplified if detected at baseline but increase more than four-fold at D64.

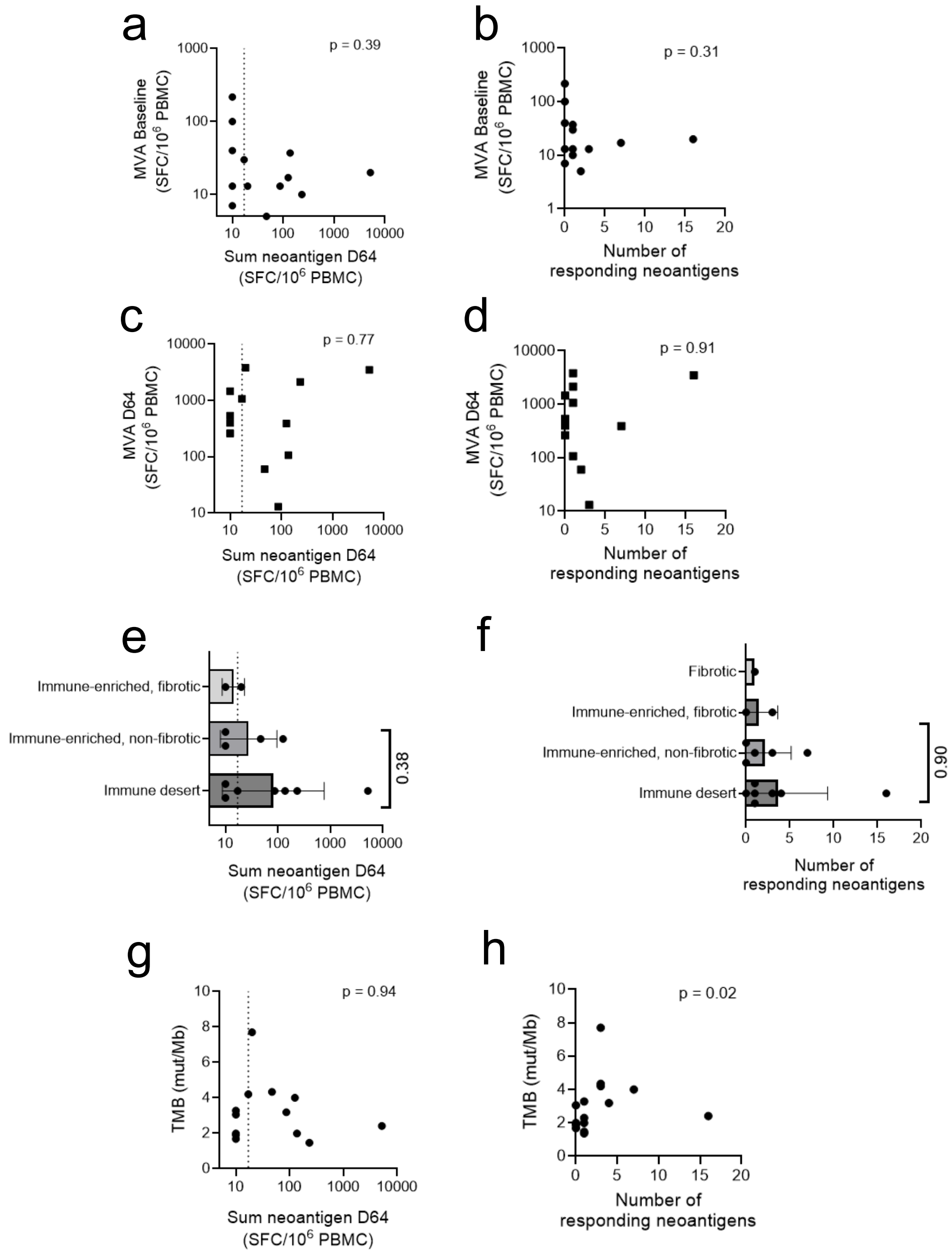

Supplementary Figure 4. Association between T cell responses to MVA backbone, TME subtype and TMB and neoantigen-specific T cell responses to treatment. a, b: Pre-existing T cell responses to the MVA vector backbone, calculated as the background-subtracted SFC per million PBMC in response to the vaccinia/MVA peptide pool at baseline vs (a) the magnitude of neoantigen-specific T cell responses, calculated as the sum of background-subtracted SFC per million PBMC across all responding neoantigens at D64 measured by *ex vivo* IFN-γ ELISpot or (b) the breadth of neoantigen-specific T cell responses, calculated as the number of responding neoantigens per patient. **c, d,** Treatment-associated T cell responses to the MVA vector backbone, calculated as background-subtracted SFC per million PBMC in response to the vaccinia/MVA peptide pool at D64 vs (c) magnitude and (d) breadth of neoantigen response. **e, f,** TME subtype determined by TumorPortrait^{TM} vs (e) magnitude and (f) breadth of neoantigen response. **g, h**, TMB in in mutations per Mb of tumor sample collected at surgery vs (g) magnitude and (h) breadth of neoantigen response. Statistical analysis: p-values were obtained using non-parametric tests on log-transformed values. Spearman's rank correlation was applied for panels (a–c). For panel (d), a Mann–Whitney test was performed between TME categories with ≥3 samples per group. Source data are provided in the source data file.

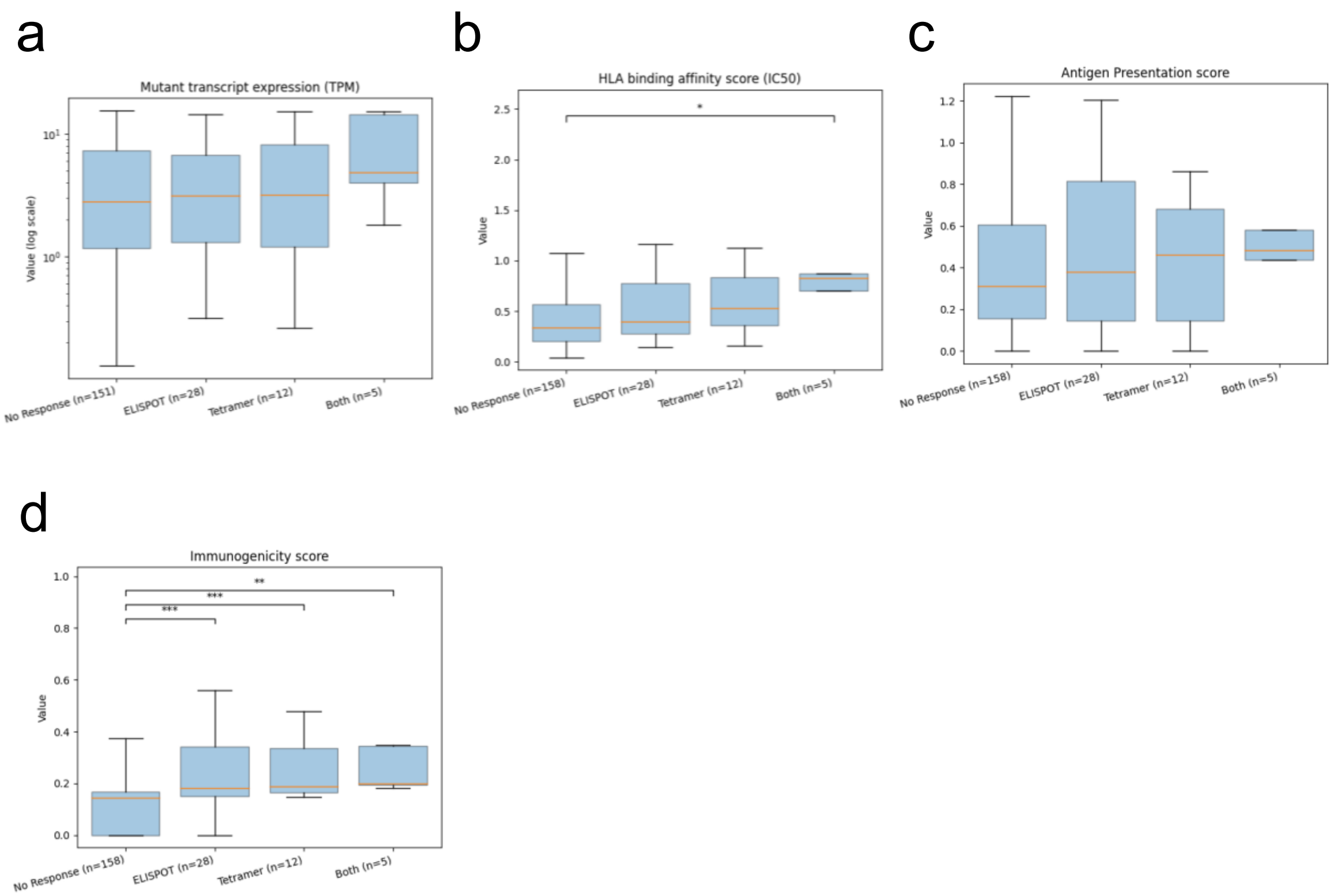

Supplementary Figure 5. Distribution of predicted determinants of immunogenicity according to vaccine-induced T-cell responses in patients. Distribution of **a,** target expression levels in transcripts per million, **b,** normalized HLA-binding affinity values, **c,** predicted antigen-presentation scores and **d,** global immunogenicity score integrating predicted antigen presentation, target expression and predicted probability of T-cell recognition for vaccine neoantigens with no detectable vaccine-induced de novo response or amplification ("No response"), targets with responses detected by ELISPOT ("ELISpot"), targets with responses detected by tetramer staining ("Tetramer"), and targets with responses detected by both assays ("Both"). Analysis was performed for eligible targets scored with the last version of the prediction tool. Boxes show the interquartile range (IQR; Q1–Q3), with the center line indicating the median. Whiskers extend to the most extreme observations within 1.5 × IQR of the lower and upper quartiles. Statistical significance was assessed using a two-sided Mann–Whitney U (Wilcoxon rank-sum) test. Asterisks denote significance levels: * $P < 0.05$, ** $P < 0.01$, *** $P < 0.001$. Source data are provided in the source data file.

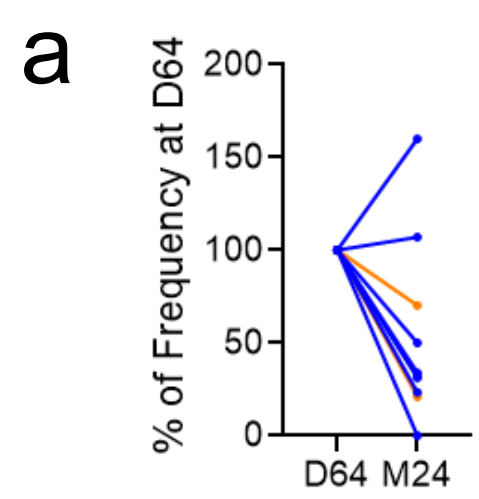

Supplementary Figure 6. Longitudinal analysis of neoantigen-specific CD8⁺ T cells. a, Change in frequency of tetramer⁺ cells between D64 and M24. Each line is one tetramer, blue lines are neoantigen-specific tetramers and orange lines MVA-specific tetramers. **b**, Kinetics and phenotype of individual tetramers. The pie charts indicate the proportion of Tn, Tcm, Tem and Temra within each tetramer⁺ cell population at D64, D211 and M24. Source data are provided in the source data file.

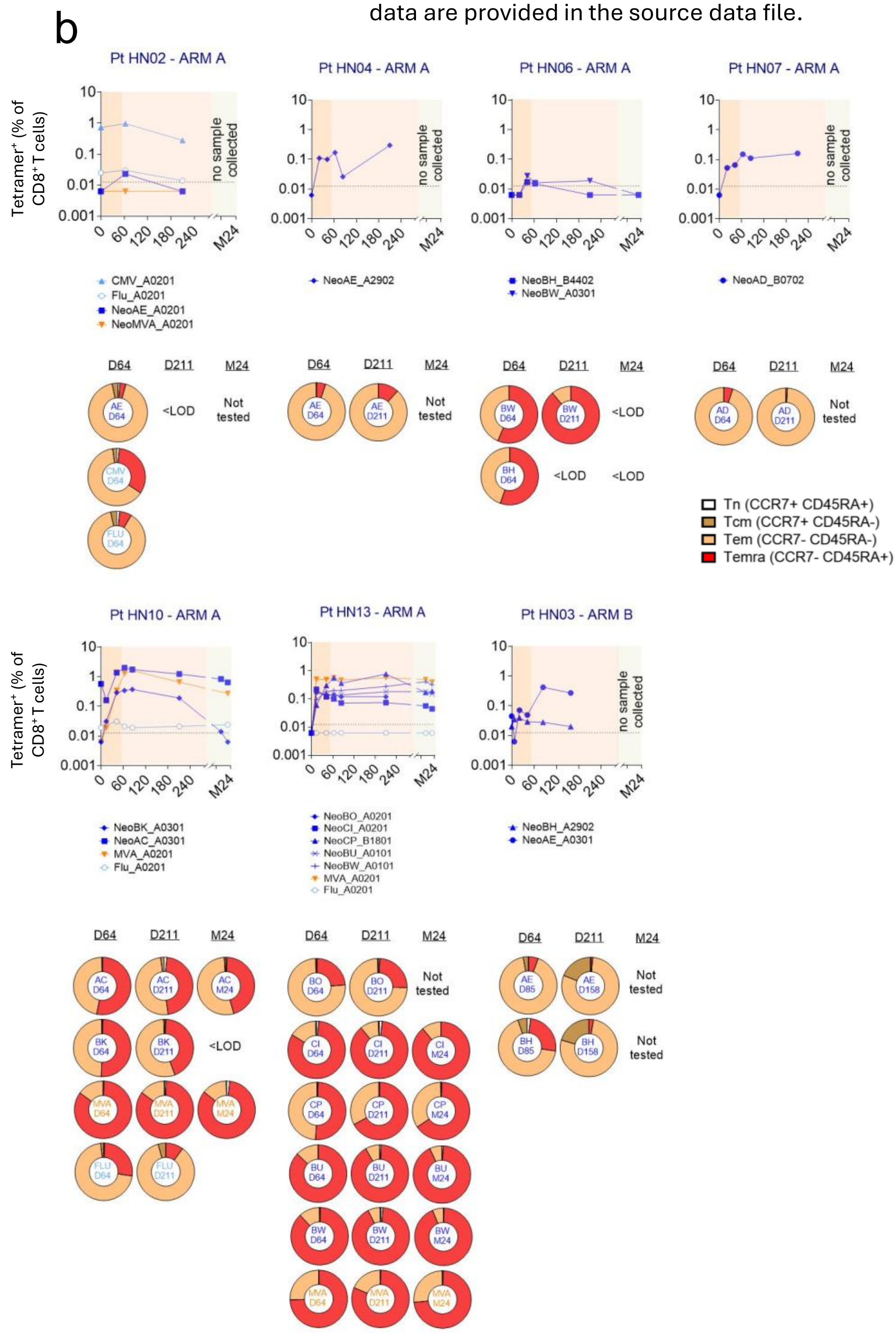

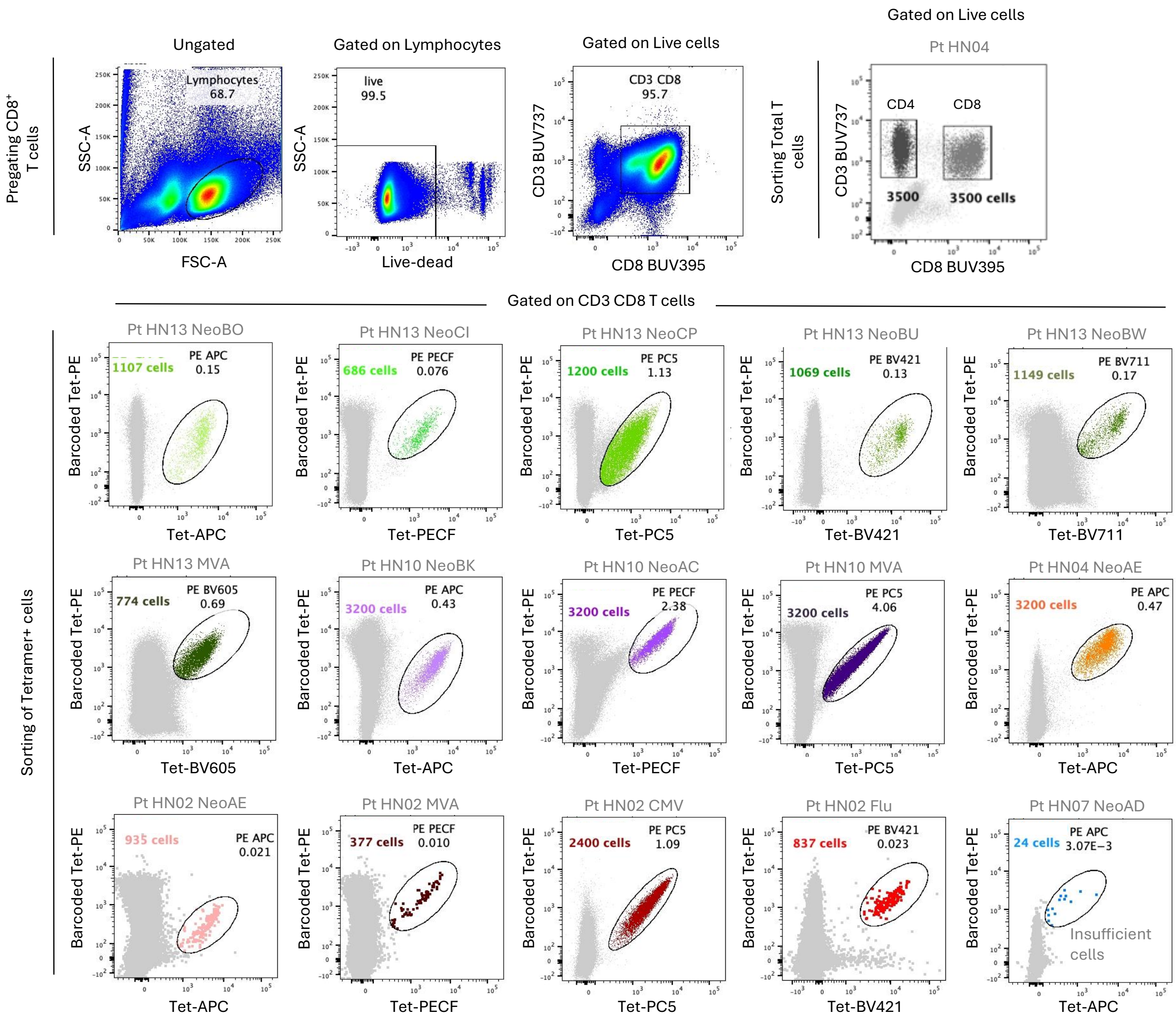

Supplementary Figure 7. Neoantigen-specific CD8⁺ T cell sorting strategy.

Tetramer⁺ cells were determined by pregating of CD8⁺ T cells followed by gating of cells double positive for PE-labeled barcoded tetramer and Tetramer labeled with the indicated fluorophore. The percentage of double positive Tetramer⁺ cells is indicated above the gate. The total number of sorted cells is on the top left of the graph. Total T cells were sorted from one patient as CD3⁺ CD8⁺ and CD3⁺ CD8⁻ fractions.

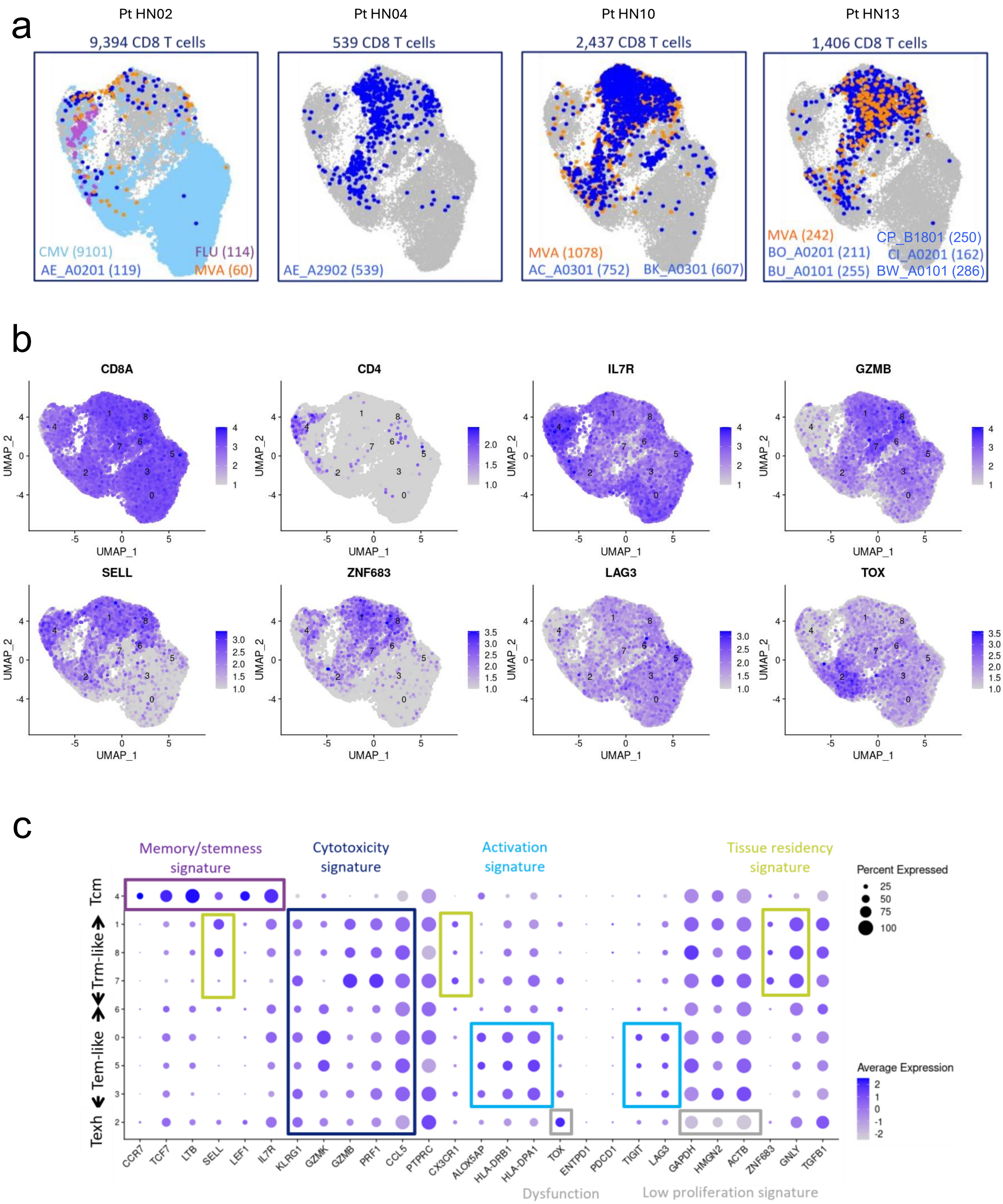

Supplementary Figure 8. scRNAseq analysis of tetramer⁺ T cells. 9 neoepitope-specific Tetramer⁺ populations from 4 patients as well as 5 virus-specific control Tetramer⁺ populations (3 MVA, 1 CMV and 1 Flu) were sorted at D64 and analyzed by scRNAseq. **a**, Overlay of cells sequenced from each patient on the global UMAP. For each patient, the total number of cells and the number of cells for each tetramer is indicated. Neoantigen tetramers are in dark blue. **b**, Expression of selected lineage markers on the UMAP. **c**, cluster annotation based on expression levels of selected lineage markers

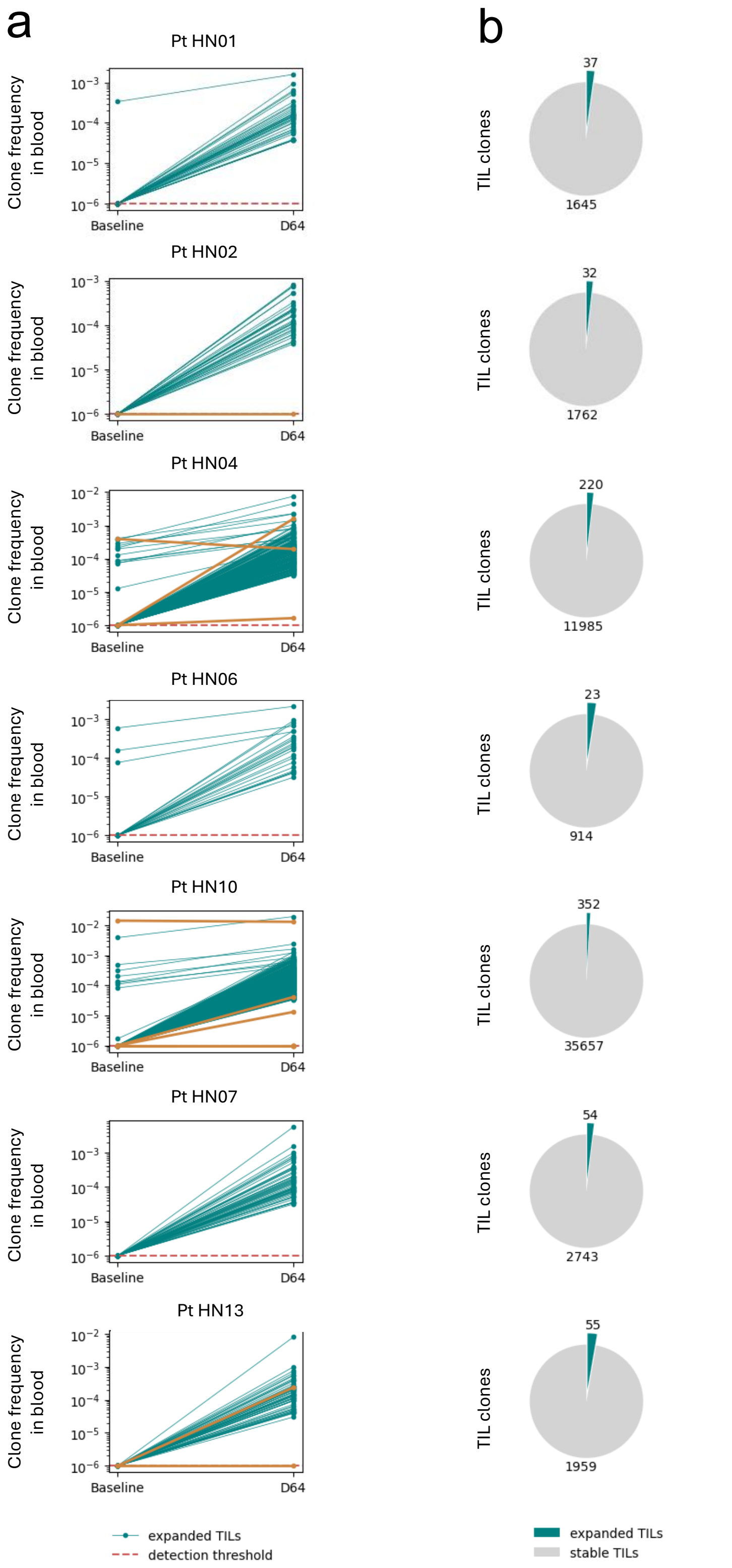

Supplementary Figure 9. Expansion of tumor-infiltrating T cell clones after TG4050 treatment. a, Frequency of tumor-infiltrating T cell clones expanded in the blood at D64 compared to baseline. One green line is one expanded TIL clone. Neoantigen-specific T cell clones validated by scRNAseq analysis of tetramer^{+} cells are indicated in orange. **b,** Proportion of TIL clones found expanded in the blood between baseline and D64 relative to all TIL clones. The number of expanded clones is indicated above the green slice, the number of TIL clones not expanded in the blood after treatment is indicated under the grey slice. Source data are provided in the source data file.

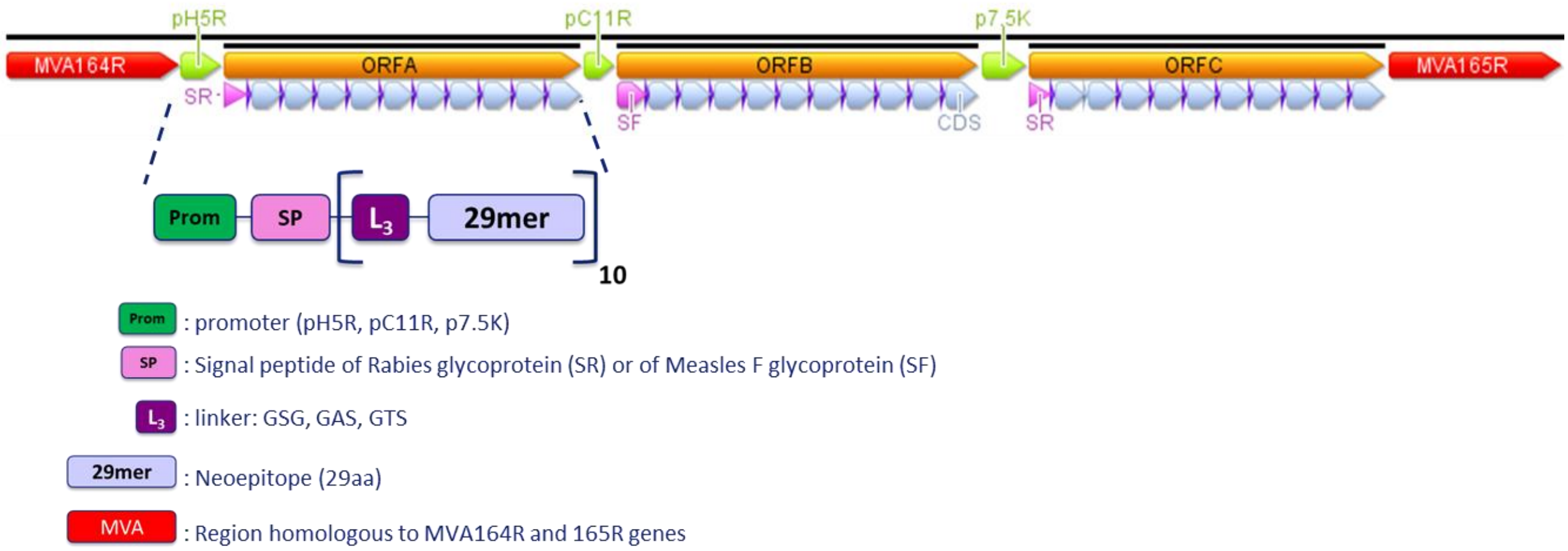

Supplementary Figure 10 . Schematic representation of TG4050 expression cassettes

Supplementary Table 1. Analysis sets

Population	Definition per protocol	Primary dataset	Total number of patients (Arm A/B)
**Full analysis set** (FAS): **Intent-to-treat** (ITT)	All randomized patients	Demographic and baseline characteristics	**N=33** (17/16)
**Safety population** (SAF)	All randomized patients who received at least one injection of TG4050	Safety analysis	**N=19** (17/2)
**Per protocol** (PP)	Minimum exposure before recurrence: Arm A: patients who completed their 6 weekly injections of TG4050<br>Arm B: patients who completed their 6 weeks follow-up	DFS	**N=32** (16/16)

Supplementary Table 2. Tumor Portrait™ analysis of tumor samples collected at surgery

Arm	Patient ID	Treated	PDL1	TMB	TME functional class	Active Immune infiltration	Tumor cell proliferation
Arm A	Pt HN01	Yes	38	2.30	Fibrotic	Moderate	Medium
Arm A	Pt HN16	Yes	23	1.37	Immune enriched, non-fibrotic	HIgh	Medium
Arm A	Pt HN17	Yes	29	1.99	Immune enriched, fibrotic	Moderate	Low
Arm A	Pt HN02	Yes	28	3.19	Immune Desert	Moderate	Medium
Arm A	Pt HN04	Yes	<1	1.99	Immune Desert	Moderate	Low
Arm A	Pt HN05	Yes	24	3.42	Immune Desert	Moderate	Medium
Arm A	Pt HN06	Yes	<1	4.20	Immune Desert	Low	Medium
Arm A	Pt HN10	Yes	80	7.70	Immune enriched, fibrotic	Moderate	Medium
Arm A	Pt HN14	Yes	48	1.46	Immune Desert	Moderate	Low
Arm A	Pt HN15	Yes	43	1.68	Immune enriched, non-fibrotic	Moderate	Medium
Arm A	Pt HN07	Yes	81	4.34	Immune enriched, non-fibrotic	Moderate	Medium
Arm A	Pt HN08	Yes	27	3.28	Immune Desert	Low	Medium
Arm A	Pt HN09	Yes	24	1.90	Immune enriched, non-fibrotic	Moderate	Low
Arm A	Pt HN18	Yes	19	3.16	Fibrotic	Moderate	Medium
Arm A	Pt HN11	Yes	26	4.00	Immune enriched, non-fibrotic	Moderate	Low
Arm A	Pt HN12	Yes	<1	3.05	Immune Desert	Low	Medium
Arm A	Pt HN13	Yes	48	2.41	Immune Desert	Low	High
Arm B	Pt HN20	No	0	2.10	Immune Desert	Low	Medium
Arm B	Pt HN19	Yes	44	2.91	Immune enriched, non-fibrotic	Moderate	Low
Arm B	Pt HN21	No	90	3.28	Immune enriched, non-fibrotic	HIgh	High
Arm B	Pt HN22	No	<1	1.90	Immune Desert	Moderate	Medium
Arm B	Pt HN23	No	<1	0.03	Immune enriched, non-fibrotic	Moderate	Medium
Arm B	Pt HN03	Yes	<1	3.56	Immune Desert	Moderate	Low
Arm B	Pt HN24	No	<1	4.26	Immune Desert	Low	Medium
Arm B	Pt HN25	No	<1	1.60	Immune Desert	Moderate	Medium
Arm B	Pt HN26	No	15	3.02	Immune Desert	Moderate	Medium
Arm B	Pt HN27	No	17	3.64	Immune Desert	Low	Medium
Arm B	Pt HN28	No	<1	2.77	Immune Desert	Moderate	Medium
Arm B	Pt HN29	No	<1	3.02	Immune Desert	Moderate	Medium
Arm B	Pt HN30	No	1	3.36	Immune Desert	Moderate	Medium
Arm B	Pt HN31	No	72	5.24	Immune Desert	Moderate	Medium
Arm B	Pt HN32	No	48	7.95	Fibrotic	Moderate	Medium
Arm B	Pt HN33	No	50	0.34	Immune Desert	Moderate	Low

Supplementary Table 3. Overview of T cell responses detected for each patient by ex vivo IFN-γ ELISpot and pMHC-I tetramer

			Neoantigens				MVA	
Arm	Patient ID	Vaccine neoantigens	*Ex vivo* IFN-γ ELISpot	pMHC-I Tetramer	Responding neoantigens	Overall response	*Ex vivo* IFN-γ ELISpot	pMHC-I Tetramer
A	HN01	26	Not tested	1/23	1	De novo	Not tested	De novo
	HN16	30	Not tested	1/11	1	De novo	Not tested	Not tested
	HN17	30	0/30	0/20	0	No response	Amplified	Amplified
	HN02	27	3/27	1/12	4	De novo	No response	Not tested
	HN04	30	1/30	1/9	1	De novo	Amplified	Not tested
	HN05	30	Not tested	Not tested	N/A	N/A	Not tested	Not tested
	HN06	30	1/30	2/12	3	De novo	De novo	Not tested
	HN10	30	1/30	2/12	3	De novo	De novo	De novo
	HN14	30	1/30	0/5	1	Amplified	De novo	Not tested
	HN15	17	0/17	0/4	0	No response	De novo	Not tested
	HN07	30	2/18	2/19	3	De novo	De novo	Not tested
	HN08	30	0/30	1/18	1	De novo	De novo	Not tested
	HN09	30	0/30	0/16	0	No response	De novo	Not tested
	HN18	30	Not tested	Not tested	N/A	N/A	Not tested	Not tested
	HN11	30	7/30	0/29	7	De novo	Amplified	Not tested
	HN12	30	0/30	Not tested	0	No response	Stable	Not tested
	HN13	30	16/30	5/18	16	De novo	De novo	De novo
B	HN19	30	Not tested	0/10	0	No response	Not tested	Not tested
	HN03	30	Not tested	2/33	2	De novo	Not tested	Not tested
Total Arm A			8 / 13 (61.5%)	9 / 14 (64.3%)	3 [1-16]	11 / 15 (73.3%)	11 / 13 (84.6%)	4 / 4 (100%)
Total all treated			8 / 13 (61.5%)	10 / 16 (62.5%)	2 [1-16]	12 / 17 (70.6%)	11 / 13 (84.6%)	4 / 4 (100%)

Supplementary Table 4. Sample ID correspondance for scRNAseq data in GSE327189

GEO ID	MANUSCRIPT ID	PATIENT ID	ANTIGEN	HLA	SEQUENCE	COMMENTS
02-YV-9	Pt HN02 NeoAE-Tet	HN02	NeoAE	A0201	YLTDWVMTV	
02-MVA	Pt HN02 MVA-Tet	HN02	MVA	A0201	KVDDTFYYV	
02-CMV	Pt HN02 CMV-Tet	HN02	CMV	A0201	NLVPMVATV	
02-FLU	Pt HN02 Flu-Tet	HN02	Flu	A0201	GILGFVFTL	
11-AY-9	Pt HN04 NeoAE-Tet	HN04	NeoAE	A2902	ATQNQLLFY	
01-CL-9	Pt HN07 NeoAD-Tet	HN07	NeoAD	B0702		Not enough cells for analysis
33-AK-9	Pt HN10 NeoBK-Tet	HN10	NeoBK	A0301	ACYDTVNNK	
33-TK-9	Pt HN10 NeoAC-Tet	HN10	NeoAC	A0301	TVFEYGTRK	
33-MVA	Pt HN10 MVA-Tet	HN10	MVA	A0201	KVDDTFYYV	
21-GA-9	Pt HN13 NeoBO-Tet	HN13	NeoBO	A0201	GLCHQLTTA	
21-WV-9	Pt HN13 NeoCI-Tet	HN13	NeoCI	A0201	WLDGDLYHV	
21-CY-9	Pt HN13 NeoCP-Tet	HN13	NeoCP	B1801	CEDMLLCSY	
21-FY-10	Pt HN13 NeoBU-Tet	HN13	NeoBU	A0101	FTSDEVSTSY	
21-RY-9	Pt HN13 NeoBW-Tet	HN13	NeoBW	A0101	RTSNLNYQY	
21-MVA	Pt HN13 MVA-Tet	HN13	MVA	A0201	KVDDTFYYV	

Clinical Study Protocol

CONFIDENTIAL

**A randomized phase I/II trial in patients with newly-diagnosed, locoregionally advanced, HPV-negative, squamous cell carcinoma of the head and neck (SCCHN) evaluating a mutanome-directed immunotherapy initiated at completion of primary treatment or at time of recurrence**

Study phase: I/II

PROTOCOL N° TG4050.02
Final Version 9.0 / 12 February 2025

EU CT N°: 2023-508561-33
EUDRACT N°: 2018-003267-58

COORDINATING INVESTIGATORS:

Prof Christian OTTENSMEIER
Head and Neck Center
Institute of Systems, Molecular and Integrative Biology
University of Liverpool
6 West Derby Street
Liverpool L7 8TX, UK
Phone: [redacted]
Email: [redacted]

Prof Christophe LE TOURNEAU
Department of Drug Development and Innovation (D3i)
Paris-Saclay University
Institut Curie
26, rue d'Ulm
75248 Paris Cedex 05 France
Phone: [redacted]
Email: [redacted]

SPONSOR:

Transgene
Boulevard Gonthier d'Andernach
Parc d'Innovation - CS80166
67405 Illkirch Graffenstaden Cedex - FRANCE

PERSON TO CONTACT:

[redacted]
Clinical Development
Transgene S.A.
[redacted]

The confidential information contained in this document is the property of Transgene. It is provided to you in confidence, for review by you, your staff, regulatory authorities, and members of ethics committees or institutional review boards. It is understood that this information will not be disclosed to any other third party, in any form, without prior authorization from Transgene, except to the extent necessary to obtain the written informed consent from the persons to whom the Investigational Medicinal Product may be administered.

INVESTIGATORS / STUDY ADMINISTRATIVE STRUCTURE

NAMES AND CONTACT DETAILS

Coordinating Investigators

Prof Christian Ottensmeier
Head and Neck Center
Institute of Systems, Molecular and Integrative Biology
University of Liverpool
6 West Derby Street
Liverpool L7 8TX, UK

Prof Christophe Le Tourneau
Department of Drug Development and Innovation (D3i)
Paris-Saclay University
Institut Curie
26, rue d'Ulm
Paris, France

Centers / Investigators: The complete Investigator list is available in the Trial Master File at Transgene.

Transgene's Staff

<table>
<tr><th>Chief Medical Officer</th><th>Pharmacovigilance Physician</th><th>Clinical Project Manager</th></tr>
<tr><td>[redacted]</td><td>[redacted]</td><td>[redacted]</td></tr>
<tr><td colspan="3">Clinical Development
Transgene
Boulevard Gonthier d'Andernach
Parc d'Innovation - CS80166
67405 Illkirch Graffenstaden Cedex - FRANCE
[redacted]</td></tr>
</table>

Statistician	Clinical Trial Manager	Clinical Supplies Manager
[redacted]	[redacted]	[redacted]

A complete list of details for the Contract Research Organization (CRO), the central laboratory/ies and the independent review committee(s) is available in the Investigator Site File and in Transgene files.

During the study, if applicable, the administrative structure will be updated in the Investigator Site File and in Transgene files.

DOCUMENT APPROVAL

SPONSOR'S OFFICER(S)

[redacted]
Chief Medical Officer

[redacted]

[redacted]
Clinical Project Manager

[redacted]

STATISTICIAN

[redacted]
Statistician

[redacted]

COORDINATING INVESTIGATORS

Prof Christian OTTENSMEIER, MD, PhD

[redacted]

Prof Christophe LE TOURNEAU, MD, PhD

[redacted]
Signature

SYNOPSIS

Sponsor: Transgene
IMP: TG4050, a Modified Virus Ankara (MVA) based mutanome-directed individualized immunotherapy

Study Title
A randomized phase I/II trial in patients with newly diagnosed, locoregionally advanced, HPV negative, squamous cell carcinoma of the head and neck (SCCHN) evaluating a mutanome-directed immunotherapy initiated at completion of primary treatment or at time of recurrence

Study N°: TG4050.02

EU CT N°: 2023-508561-33**; EUDRACT N°:** 2018-003267-58

Coordinating / Principal Investigators:
Prof Christian OTTENSMEIER, Prof Christophe LE TOURNEAU

Investigational Centers / Countries: approximately 15 centers in United Kingdom, France, Spain, and in the US

**Study Period:** Q3 2019 - Q4 2031	**Clinical Phase:** I/II

Objectives

Primary objective:
Phase I part: To evaluate the safety and tolerability of multiple subcutaneous injections of a mutanome-directed active immunotherapy (TG4050) in patients with newly diagnosed, locoregionally advanced, HPV-negative SCCHN initiated at completion of primary treatment (Arm A) or at the time of recurrence (Arm B).
Phase I/II: To compare the efficacy of TG4050 (experimental arm) and watchful waiting (control arm) based on two-year Disease-Free Survival (DFS) rate in patients with newly diagnosed, locoregionally advanced, HPV-negative, SCCHN after completion of primary treatment.

Secondary objectives:
Phase I part: To evaluate:

- Failure to provide TG4050
- Failure to treat with TG4050
- Disease-Free Survival (DFS)
- Tumor response by using RECIST 1.1 in patients with recurrent cancer
- Progression Free Survival (PFS) in patients with recurrent cancer

Phase I/II:

- To compare the arms with respect to
  - Distant Metastases-Free Survival (DMFS)
  - Loco-Regional Relapse-Free Survival (LRFS)
  - Safety profiles
- To evaluate the Overall Response Rate (ORR) by using RECIST 1.1 in patients with recurrent disease (patients randomized to control arm and treated with TG4050 in combination with SOC)
- To evaluate the success rate in providing TG4050 for patients eligible for randomization

Translational research:
Phase I part: To evaluate the following endpoints:

- Tumor mutational burden (TMB) and neoantigen frequency at Baseline and at recurrence
- Changes in circulating immune cells

- Induction of tumor specific T cells
- T cell changes in tumor immune infiltrate and environment at baseline and at recurrence
- Quantification of tumor and immune-related biomarkers

Phase I/II: To evaluate the following endpoints:

- Quantification of tumor and immune-related biomarkers including but not limited to PD-L1 expression, tumor mutational burden (TMB)
- Changes in circulating immune cells
- Induction of antigen specific T cells against neoantigens
- Changes in tumor immune infiltrate and microenvironment at baseline and at recurrence
- Circulating tumor DNA prior and during treatment / observation period

For all objectives of phase I/II, data from phase I part and phase II part will be pooled.

Methodology

This is a multicenter, open-label, two arms, randomized, phase I/II study in patients with newly diagnosed locoregionally advanced, HPV-negative SCCHN. Locoregionally advanced SCCHN is defined as stage III or IV disease, amenable to surgery (AJCC 8th edition). The study is evaluating TG4050, a mutanome-directed active immunotherapy based on a MVA specifically designed to target tumor specific antigens identified in each patient.
Phase I part: Patients will be randomized to Arm A (treatment start at completion of primary treatment) or to Arm B (treatment start at the time of recurrence). Patients will be stratified at randomization based on a dynamic method (minimization) according to prior cisplatin treatment (no versus yes), disease stage (III versus IV, based on pathological staging) and center.
Phase II part: Patients will be randomized to the experimental arm (treatment start at completion of primary treatment) or to the control arm (watchful waiting). Patients will be stratified at randomization based on a dynamic method (minimization) according to disease stage (III versus IVA versus IVB, based on pathological staging), prior cisplatin treatment (no versus yes), localization of primary tumor (oropharynx versus oral cavity versus larynx versus hypopharynx), PD-L1 status defined by combined positivity score (CPS <1 versus CPS ≥1 <20 versus CPS ≥20) and center. The study will consist of two periods:

- A screening period starting at surgery in newly diagnosed patients with locoregionally advanced, HPV-negative SCCHN. Locoregionally advanced SCCHN is defined as stage III or IV disease, amenable to surgery (AJCC 8th edition). **During the screening period, patients will undergo standard adjuvant therapy.**

- A treatment period:
  - Phase I part:
    Patients will be randomized within 6 weeks after complete response (CR) documentation in a 1:1 ratio to receive TG4050 either at completion of primary treatment (Arm A) or at time of recurrence (Arm B).

    Arm A: Within 1 week after randomization (period may be prolonged during safety run-in phase) patients will start treatment with TG4050 as a single agent for up to a total of 20 injections or until recurrence documented clinically and by imaging or until unacceptable toxicity including occurrence of a dose-limiting toxicity or patient withdrawal for any reason, whichever occurs first. At recurrence patients will then be offered TG4050 in combination with standard of care (SOC) for up to a total of 20 injections, or until documented disease progression according to RECIST 1.1, or until unacceptable toxicity including occurrence of a dose-limiting toxicity or patient withdrawal for any reason

whichever occurs first. If unacceptable toxicity was leading to treatment discontinuation before recurrence patient will be treated with SOC only after recurrence.
Arm B: At recurrence documented clinically and by imaging, patients will receive TG4050 in combination with SOC for up to a total of 20 injections, or until documented disease progression according to RECIST 1.1, or until unacceptable toxicity including occurrence of a dose-limiting toxicity or patient withdrawal for any reason whichever occurs first.

- Phase II part:
  Patients will be randomized within 6 weeks after complete response (CR) documentation in a 1:1 ratio to either receive TG4050 (experimental arm) or to watchful wait (control arm).

  Experimental arm: Within 1 week after randomization, patients will start treatment with TG4050 as a single agent for up to a total of 20 injections or until recurrence documented clinically and by imaging or until unacceptable toxicity including occurrence of a dose-limiting toxicity or patient withdrawal for any reason, whichever occurs first.
  Control arm: Patients will undergo watchful waiting until recurrence documented clinically and by imaging. At recurrence, patients will start TG4050 in combination with SOC for up to a total of 20 injections or until documented disease progression according to RECIST 1.1, or until unacceptable toxicity including occurrence of a dose-limiting toxicity or patient withdrawal for any reason whichever occurs first.

Graphical synopsis Phase I part

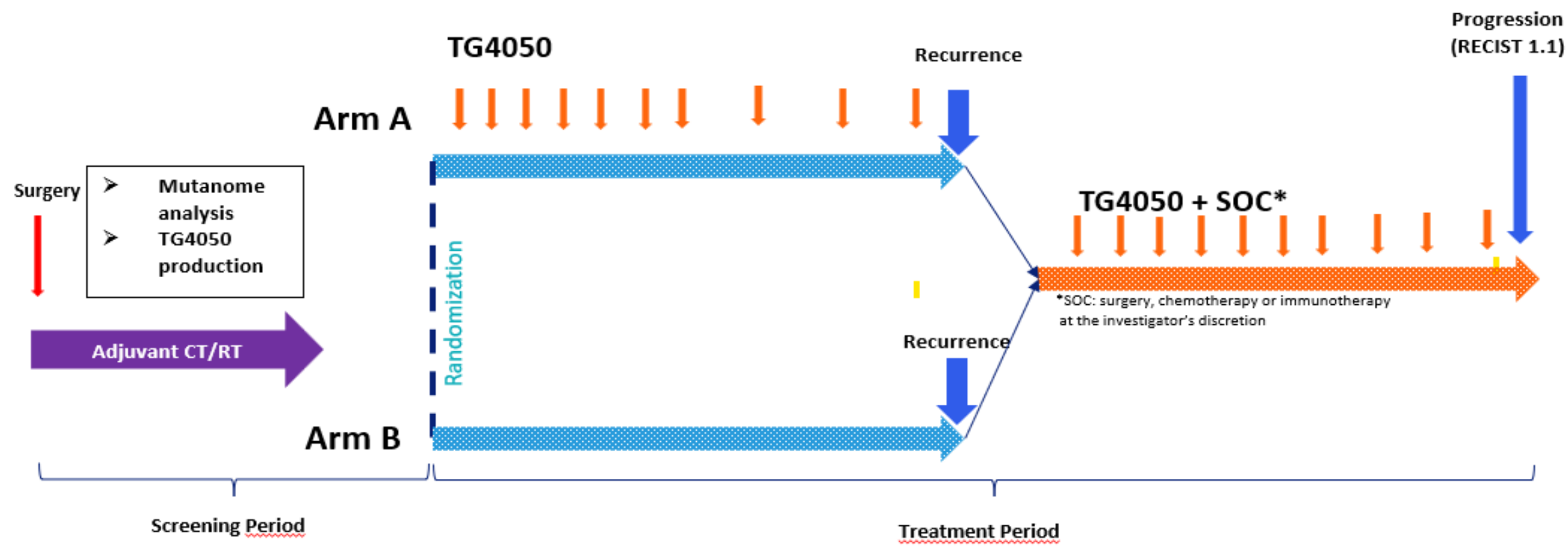

Graphical synopsis Phase II part

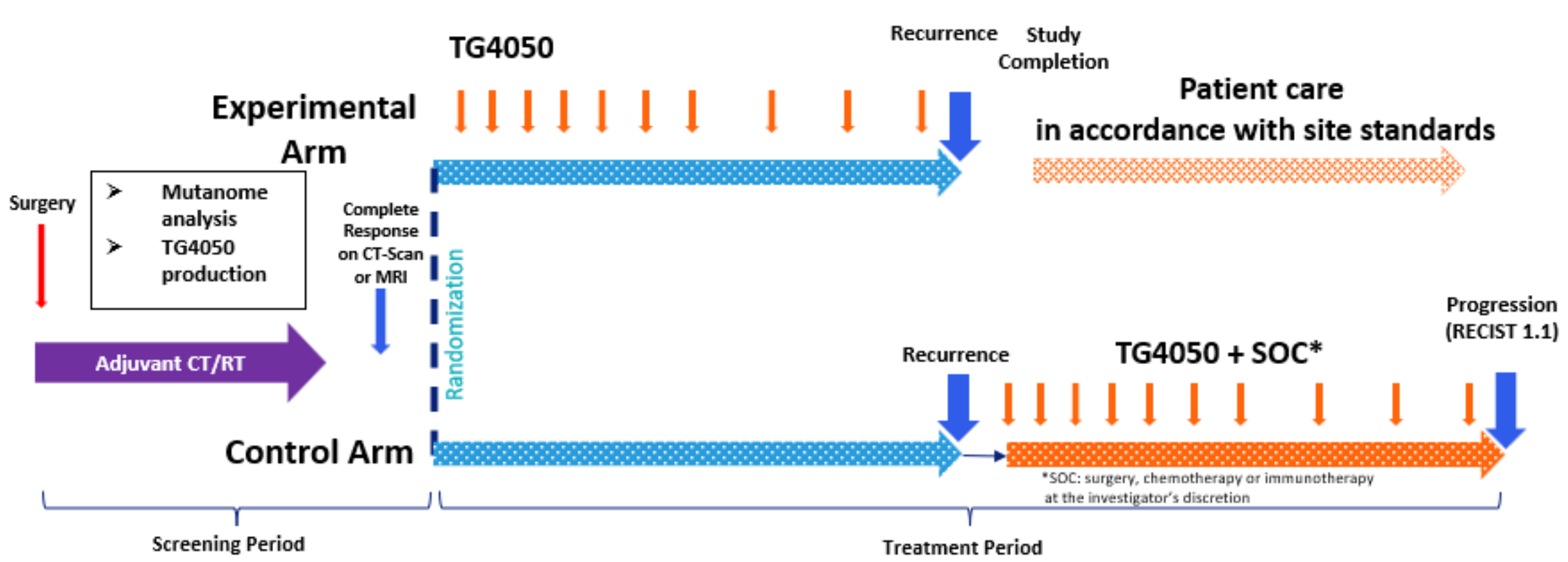

In the phase I part, a safety run-in phase will be conducted for the first 3 treated patients in each arm who will be observed during 6 weeks after the first administration of TG4050. During this phase and per arm, each patient will be monitored for 2 weeks after the first injection of TG4050 (and having received 2 injections of TG4050) before the next patient can be enrolled in the same arm.
Enrollment of the next patients will be allowed if none of the patients has experienced during the six weeks run-in phase a dose-limiting toxicity defined as:

- any treatment-emergent AE grade ≥3 toxicity that is at least possibly related to TG4050, with the following exception: pre-existing grade 2 fatigue at baseline that would worsen up to grade 3,
- any death.

The occurrence of any of those events will trigger a study hold. An evaluation of the cause of toxicity will be performed and corrective measures, if appropriate, will be proposed before recruitment in the study can be reinitiated.

Beyond the safety run-in phase, a dose-limiting toxicity is defined, in the phase I part and in the phase II part, as any treatment-emergent AE ≥3 toxicity that is at least possibly related to TG4050., with the following exceptions: pre-existing grade 2 fatigue at baseline that would worsen up to grade 3.

In any case, patients experiencing a dose-limiting toxicity will discontinue TG4050 treatment permanently.

An Independent Data Monitoring Committee (IDMC) will be set up for the purpose of reviewing patient safety data and the conduct of the study.

For the phase I part, the IDMC will review the safety of the first 3 treated patients in arm A and of the first 3 treated patients in Arm B who have completed their first 6 weeks of TG4050 treatment or have discontinued the study treatment due to treatment-related toxicity. The IDMC will assess/recommend whether it is possible to pursue patient enrolment as per study protocol.
In addition to the reviews of the first 3 patients treated in Arm A and Arm B, respectively the IDMC will review safety data of all patients included every 3 months during the first year and every 6 months thereafter. With the initiation of the phase II part, safety data of patients in the phase I part will be continued to be reviewed at the schedule for the phase II part.
Ad hoc meetings may be held upon necessity.

For the phase II part, the IDMC will review safety data of all patients included every 12 months and at final analysis.
Ad hoc meetings may be held upon necessity.

Number of Patients
Phase I part:

- Screening period: assuming a drop-out rate of at least 50 %, an approximate number of 80 patients will be screened.
- Treatment period: up to 40 patients will be randomized between arm A and arm B.

Phase II part:

- Screening period: assuming a drop-out rate of at least 50 %, an approximate number of 90 to 100 patients will be screened.
- Treatment period: a total of at least 46 patients will be randomized between the experimental arm and the control arm (23 patients per arm).

Inclusion criteria
Screening period

1. Signed written informed consent in accordance to ICH-GCP and national/local regulation before undertaking any protocol-specific procedures

2. Newly diagnosed stage III or IV, amenable to surgery (AJCC 8th edition), squamous-cell carcinoma of the oral cavity, oropharynx, hypopharynx or larynx excluding HPV-positive primaries
3. Patients must have undergone gross total resection of the primary tumor
4. Patients must be eligible for adjuvant therapy
5. a) Tumor tissue from primary tumor obtained during surgery and peripheral blood sample must be available for exome and transcriptome sequencing
   b) For phase II part tumor tissue from surgery must be available for centralized testing of PD-L1 using the 22C3 clone
6. Female or male patients, aged at least 18 years

<u>Treatment period</u>:

7. Patient must have completed adjuvant radiotherapy +/- chemotherapy and have a post-treatment CT or MRI of the head and neck and CT-scan of the chest documenting CR. Imaging has to be performed 3 months after completion of adjuvant radiotherapy +/- chemotherapy and within 6 weeks prior to randomization
8. Phase I part: Patients must have recovered from the toxicities of adjuvant therapy
   Phase II part: Patients must have recovered from toxicities of adjuvant radiotherapy +/- chemotherapy to ≤ Grade 1. Patients with ≤ Grade 2 neuropathy, dysgeusia, dysphagia, and xerostomia are eligible
9. Eastern Cooperative Oncology Group (ECOG) performance status (PS) of 0 or 1
10. Adequate hematological, hepatic and renal functions:
    - Hemoglobin ≥ 9.0 g/dL (without packed red blood cell transfusion within the prior 3 weeks)
    - Neutrophils count ≥ 1.5 x10^9/L
    - Total lymphocytes count ≥ 0.5 x10^9/L
    - Platelets count ≥ 100 x10^9/L
    - Total bilirubin ≤ 1.5 x ULN (except for patients with Gilbert's syndrome who may be included if total bilirubin ≤ 2.5 x ULN)
    - Serum transaminases: alanine aminotransferase [ALT] and aspartate aminotransferase [AST]) ≤ 2.5 x ULN
    - Calculated creatinine clearance ≥ 40 mL/min using the Cockroft & Gault formula
    - Serum albumin ≥ 30 g/L
11. No locoregional relapse or occurrence of distant metastases within 6 weeks prior to randomization
12. No active infection. For phase II part, this includes infection with mpox and SARS-CoV-2 (COVID-19) PCR positive testing within 30 days prior to randomization
13. Highly Effective contraception (i.e. methods with a failure rate of less than 1% per year) during the study period and for 3 months after the last study treatment administration for female patients of childbearing potential (WOCBP) and for male patients who are sexually active with WOCBP (See Appendix II for WOCBP definition and details on highly effective contraception methods). Highly effective contraception methods are defined as:
    • Hormonal methods of contraception including combined oral contraceptive pills, vaginal ring, injectables, implants, intrauterine devices (IUDs) such as Mirena and Nonhormonal IUDs such as ParaGard for WOCBP patient or male patient's WOCBP partner
    • Tubal ligation
    • Vasectomy
    In addition to highly effective contraception, participating male patients must use a condom during the study period and for 3 months after the last study treatment administration
14. Negative blood pregnancy test for female patients of childbearing potential (Appendix II)

Exclusion criteria

Screening period

1. a) Patients with carcinoma of the nasopharynx, squamous cell-carcinoma of unknown primary, squamous cell carcinoma that originates from the skin and salivary gland or paranasal sinus, non-squamous histologies
   b) Patients with stage I or II based on pathological staging
2. a) Prior exposure to anti-cancer vaccines, and any antibody targeting T-cell co-regulatory proteins such as anti-PD1, anti-PDL1 or anti-CTLA4 antibodies
   b) Prior radiotherapy or systemic anti-cancer therapy including investigational agents for the SCCHN under study (for phase II part)
3. Other active malignancy requiring concurrent systemic intervention
4. Patients with previous malignancies other than the target malignancy to be investigated in this trial (exceptions: non-melanoma skin cancers, early stage resected papillary thyroid cancer, and the following in situ cancers: bladder, gastric, colon, endometrial, cervical/dysplasia, melanoma, or breast) are excluded unless a complete remission was achieved at least 2 years prior to study entry. Additional therapy must not be planned or required during the study period
5. Patients with any organ transplantation, including allogeneic stem cell or bone marrow transplantation
6. Any known allergy or reaction to eggs or attributed to compounds of similar chemical or biological composition to therapeutic vaccines/immunotherapeutic products
7. Known history of positive testing for Human Immunodeficiency Virus (HIV) or known Acquired Immune Deficiency Syndrome (AIDS)
8. Clinical or laboratory history or evidence of Hepatitis C Virus (HCV) or Hepatitis B Virus (HBV) indicating acute or chronic infection
9. Any psychological, familiar, sociological or geographical condition potentially hampering compliance with the study protocol and follow-up schedule; those conditions should be discussed with the patient before registration in the trial
10. Pregnant or nursing (lactating) women, where pregnancy is defined as the state of a female after conception and until the termination of gestation, confirmed by a positive hCG laboratory test (> 10 mIU/mL)

Treatment period

11. Patients under chronic treatment with systemic corticosteroids or other immunosuppressive drugs for a period of at least 4 weeks and whose treatment was not stopped 2 weeks prior to randomization, with the exception of patients with adrenal insufficiency who may continue corticosteroids at physiological replacement dose, equivalent to ≤ 10 mg prednisone daily. Steroids with no or minimal systemic effect (topical, inhalation) are allowed
12. Phase I part: Vaccination with a live vaccine for the prevention of infectious diseases in the four-week period prior to randomization
    Phase II part: Vaccination for the prevention of infectious diseases in the four-week period prior to randomization with a live vaccine or any COVID-19-vaccine
13. Treatment with another investigational agent since the beginning of the screening period
14. Uncontrolled intercurrent illness including but not limited to ongoing or active infection, symptomatic congestive heart failure, unstable angina pectoris, cardiac arrhythmia or psychiatric illness/social circumstances that could limit compliance with study requirements

IMP: Dose, Mode of Administration

In this study, the Investigational Medicinal Product (IMP) is TG4050.

- The IMP is an individualized MVA construct, mutanome-directed immunotherapy tailored to each patient and derived from a replication deficient strain of vaccinia virus (Modified Vaccinia Virus Ankara, MVA).

- The individualized TG4050 construct will be assembled for each patient during the screening period.
- Patients will receive subcutaneous (SC) injections of TG4050 at a dose of $1x10^8$ PFU weekly for the first 6 weeks and then every 3 weeks.

  Phase I part:
  - Arm A: Within 1 week after randomization (period may be prolonged during safety run-in phase) patients will start treatment with TG4050 as a single agent for up to a total of 20 injections or until recurrence documented clinically and by imaging or until unacceptable toxicity including occurrence of a dose-limiting toxicity or patient withdrawal for any reason whichever occurs first. At recurrence patients will then be offered TG4050 in combination with standard of care (SOC) for up to a total of 20 injections, or until documented disease progression according to RECIST 1.1, or until unacceptable toxicity including occurrence of a dose-limiting toxicity or patient withdrawal for any reason whichever occurs first. If unacceptable toxicity was leading to treatment discontinuation before recurrence patient will be treated with SOC only after recurrence.
  - Arm B: At recurrence documented clinically and by imaging patients will receive TG4050 in combination with SOC for up to a total of 20 injections, or until documented disease progression according to RECIST 1.1, or until unacceptable toxicity including occurrence of a dose-limiting toxicity or patient withdrawal for any reason whichever occurs first.

  Phase II part:
  - Experimental arm: Within 1 week after randomization, patients will start treatment with TG4050 as a single agent for up to a total of 20 injections or until recurrence documented clinically and by imaging or until unacceptable toxicity including occurrence of a dose-limiting toxicity or patient withdrawal for any reason, whichever occurs first.
  - Control arm: Patients will undergo watchful waiting until recurrence documented clinically and by imaging. At recurrence, patients will start TG4050 in combination with SOC for up to a total of 20 injections or until documented disease progression according to RECIST 1.1 or until unacceptable toxicity including occurrence of a dose-limiting toxicity or patient withdrawal for any reason whichever occurs first.

Reference / Associated Therapy

Standard of care can be surgery, chemotherapy or immunotherapy at the investigator's discretion according to the applicable SmPC or product label (when applicable).

Permitted medicines and therapies:

- Any medications that are considered necessary for the patients' welfare and will not interfere with the trial medication may be given at investigator's discretion.
- The use of the following drugs will not be restricted during the course of the study: G-CSF, erythropoietin, antiemetics, and analgesics.
- The use of systemic corticosteroids during the study is restricted as follows: for short term treatment (≤7 days) of allergic reactions, or for replacement for adrenal insufficiency at doses equivalent to ≤ 10 mg prednisone daily.
- Topical, ocular, intra-articular, intranasal and inhalational corticosteroids (with minimal systemic absorption.

Non-permitted medicines and therapies during the study treatment:

- Immunosuppressive agents.
- Immunosuppressive doses of systemic corticosteroids per restrictions described above.
- Any live vaccine for the prevention of infectious diseases.
- Any other investigational agent.

Duration of Study Treatment

Phase I part:

Arm A: At completion of adjuvant therapy TG4050 will be administered for up to a total of 20 injections or until recurrence documented clinically and by imaging or until unacceptable toxicity including occurrence of a dose-limiting toxicity or patient withdrawal for any reason whichever occurs first. At recurrence patients will then be offered TG4050 in combination with standard of care (SOC) for up to a total of 20 injections, or until documented disease progression according to RECIST 1.1, or until unacceptable toxicity including occurrence of a dose-limiting toxicity or patient withdrawal for any reason whichever occurs first.

Arm B: At recurrence documented clinically and by imaging TG4050 will be administered in combination with standard of care (SOC) for up to a total of 20 injections, or until documented disease progression according to RECIST 1.1, or until unacceptable toxicity including occurrence of a dose-limiting toxicity or patient withdrawal for any reason whichever occurs first.

For both arms: at the 1st radiologic evidence of progressive disease (PD), clinically stable patients may continue TG4050 treatment at the Investigator's discretion while awaiting a confirmatory scan and / or initiation of a further antineoplastic treatment.

Phase II part:

Experimental arm: At completion of adjuvant therapy TG4050 will be administered for up to a total of 20 injections or until recurrence documented clinically and by imaging or until unacceptable toxicity including occurrence of a dose-limiting toxicity or patient withdrawal for any reason whichever occurs first.

Control arm: Patients will undergo watchful waiting until recurrence documented clinically and by imaging. At recurrence, patients will start TG4050 in combination with SOC for up to a total of 20 injections or until documented disease progression according to RECIST 1.1, or until unacceptable toxicity including occurrence of a dose-limiting toxicity or patient withdrawal for any reason whichever occurs first.

Criteria for Evaluation

Failure to provide TG4050 (phase I part)

The proportion of patients for whom TG4050 cannot be provided and the reason for not being able to provide the vaccine will be recorded.

Success rate in providing TG4050 for patients eligible for randomization (phase I/II)

The proportion of eligible patients for whom TG4050 has been provided will be evaluated.

Failure to treat rate

The proportion of patients who cannot receive TG4050 and the reason that prevents the patient from being treated will be recorded.

Safety assessment during treatment

The following evaluations will be performed:

- Physical examination including vital signs, body weight and performance status every week for the first 6 weeks and then every 3 weeks.
- AEs and Serious Adverse Events (SAEs) will be reported and graded according to NCI CTCAE, version 5 at each visit.
- Laboratory investigations will be undertaken:
  - Complete blood count (CBC) including Red Blood cells (RBC), White Blood cells (WBC) and differential, Hemoglobin (Hb) and platelets at baseline, every week up to week 6, then every 3 weeks up to Month 6 (Day 190), thereafter every 12 weeks, at the end of treatment visit and at recurrence (at recurrence: for all patients in phase I part, and for patients in the control arm in phase II part).
  - Biochemistry analyses including: AST, ALT, alkaline phosphatases, LDH, total bilirubin, serum protein, serum albumin, glucose, electrolytes (sodium, potassium, calcium, magnesium

  and bicarbonate) creatinine and creatinine clearance (calculated), at baseline, every week up to week 6, then every 3 weeks up to Month 6 (Day 190), thereafter every 12 weeks, at the end of treatment visit and at recurrence (at recurrence: for all patients in phase I part, and for patients in the control arm in phase II part).
  - Serum pregnancy test (for women of childbearing potential, Appendix II) at screening, baseline and then every 3 weeks, at the end of treatment visit, the safety follow-up visit, and at recurrence (at recurrence: for all patients in phase I part, and for patients in the control arm in phase II part).
- ECG at baseline, every 3 weeks for the first 6 weeks and then every 6 weeks up to Month 6 (Day 190), thereafter every 12 weeks, at the end of treatment visit and at recurrence (at recurrence: for all patients in phase I part, and for patients in the control arm in phase II part).

Long-Term Follow-Up after completion or stop of TG4050 treatment
- Assessment including clinical history (any relevant clinical events [e.g., immune disease]), a physical examination, and blood sampling for immune response to the vaccine 6 months after end of treatment, 12 months after end of treatment and then yearly until 5 years after end of treatment

Efficacy assessment
<u>Phase I part</u>:
  - Before recurrence: On Day 43 and then every 12 weeks during the first 24 months after randomization and every 24 weeks thereafter: physical examination of the head and neck including panendoscopy if appropriate, MRI or CT of the head and neck in the event of clinical signs or symptoms of recurrence as per standard medical practice. A histological/cytological confirmation of recurrence will be required.
  - After recurrence for patients who have started treatment with TG4050 in combination with SOC: every 6 weeks imaging.

<u>Phase II part</u>:
  - Until recurrence for the experimental arm and the control arm:
    On Day 43 and then every 12 weeks during the first 24 months after randomization and every 24 weeks thereafter: physical examination of the head and neck including panendoscopy if appropriate, MRI or CT of the head and neck in the event of clinical signs or symptoms of recurrence as per standard medical practice. A histological/cytological confirmation of recurrence required.
  - After recurrence for patients in the control arm who have started treatment with TG4050 in combination with SOC: every 6 weeks imaging until documented disease progression according to RECIST 1.1.

Immune responses
<u>Tumor, blood, skin samples collection in phase I part:</u>
- Apheresis for collection of PBMC will be performed at Baseline and 9 weeks (D64 for Arm A and R-Day 64 for Arm B) after treatment initiation. If not clinically feasible, the apheresis procedure can be replaced by a 150 ml blood draw
- Blood samples:
    - Arm A and Arm B: before surgery, at baseline, at recurrence, at progressive disease according to RECIST 1.1, at the end of treatment visit, and during Long-Term Follow-Up as specified above (6 and 12 months after end of treatment, and then yearly up to 5 years after end of treatment).
    - Arm A: on Day 15, Day 43, Day 64, Day 85, and Day 211 before recurrence and on R-Day 1, R-Day 8, R-Day 15, R-Day 43, starting from R-Day 64 every 12 weeks until progressive disease according to RECIST 1.1. Between completion of first series of 20 TG4050 administrations and recurrence every twelve weeks during the first 24 months after randomization and every 24 weeks thereafter.

- Arm B: Between randomization and recurrence every 12 weeks during the first 24 months after randomization and every 24 weeks thereafter. After recurrence on R-Day 1, R-Day 8, R-Day 15, R-Day 43, starting from R-Day 64 every 12 weeks until progressive disease according to RECIST 1.1.

- Tumor biopsies at baseline (surgery), at recurrence where this recurs and from metastatic sites where clinically possible and at progression. Tissue will be stored in FFPE, fresh frozen and for selected sites slow-frozen in DMSO. For each biopsy at least two samples of fresh tumor will be collected
- Skin biopsies from Delayed Type Hypersensitivity (DTH) sites

<u>Tumor and blood collection in phase II part</u>:

- In experimental arm and control arm: in selected centers, apheresis for collection of PBMC will be performed at Baseline and after 9 weeks of treatment, corresponding to Day 64 in the experimental arm and to R-Day 64 in the control arm, respectively. If clinically not feasible, the apheresis procedure can be replaced by a 150 ml blood draw. In centers not selected for apheresis procedure, a 150 ml blood draw is mandatory at Baseline and after 9 weeks of treatment.
- Blood samples:
    - In experimental arm and control arm before surgery, before start of adjuvant therapy, at visit completion of adjuvant therapy, at baseline, at recurrence, at the end of treatment visit, and during Long-Term Follow-Up as specified above (6 and 12 months after end of treatment, and then yearly up to 5 years after end of treatment).
    - Experimental arm: On Day 15, Day 43, starting from Day 64 every 12 weeks during the first 24 months after randomization and every 24 weeks thereafter until recurrence.
    - Control arm: On Day 15, on Day 43, starting from Day 64 every 12 weeks during the first 24 months after randomization and every 24 weeks thereafter until recurrence. After recurrence on R-Day 1, R-Day 8, R-Day 15, R-Day 43, starting from R-Day 64 every 12 weeks until progressive disease according to RECIST 1.1, and at progressive disease.
- Tumor material
    - from diagnostic biopsy, if available,
    - from surgery,
    - at recurrence where this recurs, and biopsy is feasible,
    - at recurrence from metastatic sites where biopsy is feasible.

  Tissue stored in FFPE, fresh frozen and for selected sites slow-frozen in DMSO. For each biopsy at least two samples of fresh tumor collected (except for biopsy at diagnosis).

<u>Tumor sample(s) will be collected to assess:</u>

- Density of tumor infiltrating lymphocytes (TILs) and phenotyping of the infiltrated cells (cell type and activation markers) (Immunoscoring)
- Evaluation of the expression of tumor related markers using IHC, including PD-L1, Ki-67 (Ki-67 only Phase I part)
- Transcriptomic analysis of whole tumor and purified immune cell populations
- Evaluation of tumor mutational and neoantigen load

<u>Blood samples will be collected to assess the following parameters:</u>

- Immune cell phenotype by multiparametric flow cytometry (FACS). Proposed staining of memory and naïve T cells, regulatory T cells, CD8 T cells, B cells, NK cells, innate lymphoid cells; phenotyping will include relevant activation markers and in selected patients, transcriptome analysis including T cell receptor rescue
- Quantification of immune related soluble factors, cytokines, antibody responses to neoantigens and virus
- Specific cellular immune responses against tumor antigens in Peripheral Blood Mononuclear Cells(PBMC)

- Circulating cancer and immune markers including circulating tumor DNA (ctDNA)

Skin sample (only in Phase I part)

- Skin biopsy at the site of injection for the detection of delayed type hypersensitivity (Day 8, Day 43) in patients with injection site reaction

Statistical Methods:

Populations of analyses and statistical analyses

For phase I part:

Initial Safety Sets (ISSA and ISSB): the first 3 patients randomized in arm A and in Arm B, respectively who have completed their first 6 weeks of the TG4050 treatment or have discontinued the study treatment due to treatment-related toxicity. These are the primary datasets for first IDMC reviews.

Full Analysis Set (FAS): all randomized patients will be included in the FAS. Following the intent to treat principle, patients will be analyzed according to the treatment modality and stratum that they were assigned to at randomization. The FAS is the primary population for summaries of demography and baseline characteristics and protocol deviations.

Safety Analysis Set (SAF): all randomized patients who received at least one dose of TG4050 will be included in the SAF. This is the primary dataset for analyses of safety.

Per-Protocol Sets (PP):

- Evaluable for DFS – DFS-EPP: all randomized patients who completed their first six weeks of TG4050 treatment without recurrence of disease (Arm A) or who had standard medical follow-up according to protocol during the first 6 weeks without recurrence of disease (Arm B). Patients are analyzed according to the actual treatment modality and strata.
- Evaluable for PFS – PFS-EPP: all randomized patients who completed their first six weeks of TG4050 treatment after recurrence (both arms) and have CT scan or MRI at recurrence and at least one CT scan or MRI after treatment start post-recurrence with an overall response assessment different from “Unknown” according to RECIST 1.1. Patients are analyzed according to the actual treatment modality and strata.

For phase I/II:

Full Analysis Set (FAS): all randomized patients are included in the FAS. Following the intent to treat principle, patients are analyzed according to the treatment modality and stratum that they were assigned to at randomization. The FAS is the primary population for summaries of demography and baseline characteristics and protocol deviations.

Safety Analysis Set (SAF): All patients randomized and who received one dose of TG4050 will be included in the SAF. This is the primary dataset for analyses of safety.

Per-Protocol Sets (PP):

- Evaluable for DFS – DFS-EPP: all randomized patients who completed their first six weeks of TG4050 treatment without recurrence of disease (experimental arm) or who had standard medical follow-up according to protocol during the first 6 weeks without recurrence of disease (control arm). The DFS-EPP is the primary population for DFS, DMFS and LRFS analyses. Patients are analyzed according to the actual treatment modality and strata.
- Evaluable for ORR – ORR-EPP: all patients randomized who completed after recurrence their first six weeks of TG4050 treatment in combination with SOC and have CT scan or MRI at recurrence and at least one CT scan or MRI after treatment start post-recurrence with an overall

response assessment different from “Unknown” according to RECIST 1.1. The ORR-EPP is the primary population for ORR analysis. Patients are analyzed according to the actual treatment modality and strata.

Sample size

There is no formal sample size calculation for the phase I part of this study. With the inclusion of additional patients, the purpose of this phase I/II is to evaluate efficacy in terms of Disease-Free survival, with the decision rule being based on the proportion of patients who are still free of disease 2 years after randomization. A sample size of 74 evaluable patients would result in 80% power to detect the difference between a 2Y-DFS of 85% in the experimental arm and 60% in the control arm. The sample size calculation is based on one-sided α=0.05 and testing the difference of proportions using un-pooled estimate of variance.

Therefore, it is planned to randomize in the phase II part at least 46 patients (23 in each arm) in addition to the 33 patients randomized in the phase I part to obtain a total of at least 74 evaluable patients in this phase I/II trial.

TABLE OF CONTENTS

INVESTIGATORS / STUDY ADMINISTRATIVE STRUCTURE **2**
DOCUMENT APPROVAL **3**
SYNOPSIS **4**
TABLE OF CONTENTS **16**
1. INTRODUCTION **21**
1.1 Disease background 21
1.2 Study population rationale 21
1.3 TG4050 background 23
1.4 Translational research rationale 23
1.5 Summary of the overall benefits and risks 24
1.5.1 Expected benefits *24*
1.5.2 Potential risks *24*
1.5.3 Measures taken to mitigate the risks *25*
2. OBJECTIVES **25**
2.1 Primary Objective 25
2.2 Secondary objectives 25
2.3 Translational research 26
3. STUDY DESIGN **26**
3.1 Overall study design and plan description 26
3.2 Number of centers and patients 31
3.3 Discussion of Study Design 32
3.3.1 Phase I part *32*
3.3.1.1 Phase I part - Safety 32
3.3.1.2 Phase I part - Evaluation of immune responses to TG4050 treatment 32
3.3.2 Extension to Phase I/II *33*
3.4 Dose and schedule selection 33
4. STUDY POPULATION **36**
4.1 Inclusion criteria 36
4.2 Exclusion criteria 37
4.3 Criteria for patient premature withdrawal 38
4.3.1 Premature withdrawal from the trial *38*
4.3.2 Withdrawal from study treatment *39*
4.3.3 Replacement policy *39*
4.4 Definition of end of study 39
5. REGISTRATION PROCEDURES **40**
5.1 Screening period 40
5.2 Treatment period 40
5.2.1 Baseline assessment *40*
5.2.2 Randomization *40*
6. STUDY VISITS AND PROCEDURES **41**
6.1 Screening period 41
6.1.1 Evaluation description *41*
6.1.2 Before surgery *42*
6.1.3 Visit at completion of surgery *42*
6.1.4 Visit before start of adjuvant therapy (phase II part only) *42*

6.1.5 Visit post adjuvant therapy (3 months after adjuvant therapy completion) 42
6.2 Treatment period....43
6.2.1 Evaluation description 43
6.2.2 Baseline visit 44
6.2.3 Study visits during the treatment period in the Phase I part 45
6.2.4 End of treatment visit in the Phase I part....48
6.2.5 Follow-up observation for both treatment arms (+/- 7 days) after completed End of Treatment Visit in the Phase I part....49
6.2.6 Long-Term Follow-Up after completion or stop of TG4050 treatment in the Phase I part 49
6.2.7 Study visits during the treatment period in the Phase II part....50
6.2.8 End of treatment visit in the Phase II part 53
6.2.9 Follow-up for both treatment arms (+/- 7 days) after completed End of Treatment Visit in the Phase II part....54
6.2.10 Long-Term Follow-Up after completion or stop of TG4050 treatment in the Phase II part....54
6.2.11 Safety follow-up visit (+ 7 days)....54
6.2.12 Medical care of patients after end of study 55

7. TREATMENT PLAN FOR TREATMENT PERIOD....55

7.1 Investigational Medicinal Product (IMP) characteristics 55
7.1.1 Manufacturing....55
7.1.2 Packaging, labeling and shipping 55
7.1.3 Conditions of storage and use....56
7.1.4 Preparation and administration procedure....57
7.1.5 Disposal and destruction or return 58
7.1.6 Supplier 58
7.2 IMP treatment duration....58
7.3 IMP treatment modification 59
7.4 IMP treatment compliance 59
7.4.1 Dispensing and accountability 59
7.4.2 Assessment of compliance 59
7.5 Standard of Care (SOC) 60
7.6 Concomitant medications....60
7.6.1 Prohibited and/or restricted medications during study treatment 60
7.6.2 Other restrictions and precautions....60
7.6.3 Permitted medications/therapies....60

8. EVALUATION 61

8.1 Evaluation during the screening period....61
8.2 Evaluation during the treatment period....61
8.2.1 Efficacy evaluation....61
8.2.2 Safety evaluation 61
8.2.2.1 Definitions.... 62
8.2.2.2 Time period for collection 64
8.2.2.3 Adverse event / Serious adverse event management.... 65
8.2.3 Serious Adverse Event / Serious Adverse Reaction / Suspected Unexpected Serious Adverse Reaction notification 67
8.2.4 Special situations 69
8.2.5 Laboratory values, vital signs, physical findings and other safety data....70
8.2.6 Translational research and exploratory evaluations 70

9. STATISTICAL METHODS PLANNED AND SAMPLE SIZE 71

9.1 Data sets analyzed 71
9.1.1 Screening period 71
9.1.2 Treatment period....72
9.2 Determination of sample size 73
9.3 Study endpoint....73
9.3.1 Primary endpoint 73

9.3.2 Secondary endpoints ... 74
9.3.3 Translational research ... 75
9.4 Statistical and analytical plans ... 75
9.4.1 Safety analyses for the treatment period ... 75
9.4.2 Failure to provide / to treat ... 77
9.4.3 Efficacy analyses for the treatment period ... 77
9.4.4 Translational research ... 78
9.5 Methods of analysis ... 78
9.5.1 General considerations ... 78
9.5.2 Disposition of patients ... 78
9.5.3 Demographic and baseline characteristics ... 78
9.5.4 Treatments ... 79
9.5.4.1 Primary treatment for studied disease ... 79
9.5.4.2 Study treatment ... 79
9.5.4.3 Concomitant medications ... 79
9.6 Protocol deviations ... 79
10. CHANGES IN THE CONDUCT OF THE STUDY ... 80
10.1 Protocol amendments ... 80
10.2 Premature termination or suspension of the study ... 80
11. ETHICAL CONSIDERATIONS ... 81
11.1 Independent Ethics Committee/Institutional Review Board ... 81
11.2 Informed consent ... 81
11.3 Confidentiality of patient data ... 82
11.4 Independent Data Monitoring Committee ... 82
11.4.1 Responsibilities of the Independent Data Monitoring Committee ... 82
11.4.2 Notification of the safety reviews to investigators ... 82
12. REGULATORY CONSIDERATIONS ... 83
12.1 Regulatory approval / authorization ... 83
12.2 Investigators' obligations ... 83
12.3 Insurance ... 83
13. QUALITY CONTROL AND QUALITY ASSURANCE ... 84
13.1 On site monitoring ... 84
13.2 Audit and inspection ... 84
14. DATA HANDLING AND RECORD KEEPING ... 84
14.1 Source data and documents ... 84
14.2 Case report forms ... 85
15. CLINICAL STUDY REPORT AND PUBLICATION ... 86
15.1 Clinical study report ... 86
15.2 Confidentiality of study data ... 86
15.3 Publication policy ... 86
16. ARCHIVING ... 86
16.1 Investigator site file ... 86
16.2 Trial master file ... 87
17. APPENDICES ... 88
18. REFERENCES ... 89

ABBREVIATIONS / DEFINITION OF TERMS

ABBREVIATIONS	MEANING OF ABBREVIATIONS IN DOCUMENT
AE	Adverse Event
AIDS	Acquired Immune Deficiency Syndrome
AJCC	American Joint Committee on Cancer
ALT	Alanine amino-transferase (= SGOT)
APCs	Antigen Presenting Cells
AR	Adverse reaction
AST	Aspartate amino-transferase (= SGPT)
ATC	Anatomical Therapeutic Chemical
CBC	Complete Blood Count
CI	Confidence Interval
CPS	Combined Positivity Score
CR	Complete Response
CRO	Contract Research Organization
CT	Computed Tomography
ctDNA	Circulating tumor DNA
CTL	Cytotoxic T Lymphocytes
CTFG	Clinical Trials Facilitation Group
D	Day
DFS	Disease-Free Survival
DMFS	Distant Metastases-Free Survival
DMSO	Dimethyl sulfoxide
DNA	Deoxyribonucleic acid
DTH	Delayed Type Hypersensitivity
ECG	Electrocardiogram
ECOG	Eastern Cooperative Oncology Group
eCRF	Electronic Case Report Form
EDC	Electronic Data Capture
EVCTM	EudraVigilance Clinical Trial Module
FACS	Fluorescence-activated cell sorter
FAS	Full Analysis Set
FFPE	Formalin-Fixed Paraffin-Embedded
FSH	Follicle Stimulating Hormone
GCP	Good Clinical Practice
GMO	Genetically Modified Organism
GPP2	Good Publication Practices
Hb	Hemoglobin
HBV	Hepatitis B Virus
hCG	Human Chorionic Gonadotropin
HCV	Hepatitis C Virus
HIV	Human Immunodeficiency Virus
HLA	Histocompatibility Leukocyte Antigen
HPV	Human Papilloma Virus
HRT	Hormone Replacement Therapy
IB	Investigator’s brochure
ICF	Informed Consent Form
ICH	International Conference on Harmonization
ICMJE	International Committee of medicinal Journal Editors
IDMC	Independent Data Monitoring Committee
IEC	Independent Ethics Committee
IHC	Immunohistochemistry
IMP	Investigational Medicinal Product
IRB	Institutional Review Board
ISS	Initial Safety Set
IU	International Unit
IUD	Intrauterine Devices

IUS	Intrauterine hormone-releasing System
LDH	Lactate Dehydrogenase
LPLV	Last Patient Last Visit
LRFS	Loco-regional Relapse-Free Survival
M	Month
MDSC	Myeloid-Derived Suppressor Cells
MedRA	Medical Dictionary for Regulatory Activities
mL	Milliliters
MP	Medicinal Product
MRD	Minimal Residual Disease
MRI	Magnetic Resonance Imaging
MVA	Modified Virus of Ankara
NCI-CTCAE	National Cancer Institute's Common Terminology Criteria for Adverse Events. Also referred as CTCAE in the text
NK	Natural Killer
NSCLC	Non-small Cell Lung Cancer
ORR	Overall Response Rate
PBMC	Peripheral Blood Mononuclear Cells
PD	Progressive Disease
PD-1	Programmed Death-1
PD-L1	Programmed Death Ligand-1
PFS	Progression Free Survival
PFU	Plaque Forming Unit
PP	Per-Protocol
PS	Performance Status
PT	Preferred Term
RBC	Red Blood Cells
RECIST	Response Evaluation Criteria In Solid Tumors
RNA	Ribonucleic Acid
SAE	Serious Adverse Event
SAF	Safety Analysis Set
SAR	Serious Adverse Reaction
SC	Subcutaneous
SCCHN	Squamous Cell Carcinoma of Head and neck
Sd	Standard deviation
SmPC	Summary of Product Characteristics
SOC	Standard of Care
SUSAR	Suspected Unexpected Serious Adverse Reaction
TCR	T Cell Receptor
TEAE	Treatment-Emergent Adverse Event
TSA	Tumor Specific Antigen
TIL	Tumor Infiltrating Lymphocytes
TMB	Tumor Mutational Burden
TME	Tumor Microenvironment
TMF	Trial Master File
ULN	Upper Limit of Normal
WBC	White Blood Cells
WOCBP	Women Of Childbearing Potential

1. INTRODUCTION

1.1 Disease background

Patients with locoregionally advanced, operable squamous cell carcinoma of the head and neck (SCCHN) are known to be at high risk of treatment failure, ranging from local regrowth to lymphatic spread and systemic dissemination.
One of the mainstays of treatment of locoregionally advanced SCCHN remains surgery. However, surgery alone is insufficient and improving patients' outcome requires adjuvant treatment after surgery. The standard of care is adjuvant radiotherapy or chemoradiotherapy with cisplatin which was established to be superior to radiotherapy alone in 2 independent studies conducted in Europe (Bernier J. *et al.*, 2004) and in the US (Cooper J.S. *et al.*, 2004) in case of extracapsular nodal spread or positive margins. Up to half of patients will recur despite multimodal therapy. Importantly, HPV-negative SCCHN has a much poorer prognosis than HPV-positive SCCHN (mostly originating from the oropharynx) (Ang K.K. *et al.*, 2010).

A number of pathologic poor-risk prognostic factors have been proposed in patients with resected, locally advanced SCCHN (Bernier J. *et al.*, 2006). These include positive margins of resection, extracapsular extension of disease from a lymph node, involvement of lymph nodes at level 4 or 5 from carcinomas arising in the oral cavity or oropharynx, perineural extension, vascular embolisms and/or involvement of 2 or more lymph nodes. The most significant prognostic factors for poor outcome are positive resection margins and/or extracapsular spread (Bernier J. *et al.*, 2005). Extracapsular spread was shown to double the incidence of local recurrences and distant metastases and to triple regional failure (Shaw R.J. *et al.*, 2010).

Hence, there is a clear unmet medical need for preventing treatment failures in locoregionally advanced, HPV-negative SCCHN after surgery and adjuvant therapy. One potentially attractive approach is tumor mutanome-directed immunotherapy. Recently T-cell density evaluated in primary SCCHN at diagnosis was shown to predict for outcome in HPV-positive (Ward M.J. *et al.*, 2014), as well as in HPV-negative (Nguyen N. *et al.*, 2016) patients.

1.2 Study population rationale

Patients with locoregionally advanced, operable SCCHN are known to be at high risk of treatment failure ranging from local regrowth to distant metastases. Even though adjuvant radiotherapy which can be associated with cisplatin has improved patient outcome, a significant proportion of patients will still experience treatment failure. In a large series of 2,062 patients treated with curative intent, treatment failures were recorded in 28 % of patients (Pagh A. *et al.*, 2016). Recurrence rates were higher after primary treatment of stage III-IV tumors, corresponding to 70 % of all recorded failures. The vast majority of first recurrences (62 %) occurred within the first year after primary treatment. Besides disease stage, primary site and patient characteristics such as age, performance status and pre-existent comorbidities have also prognostic implications (Brana I. and Siu L.L., 2012). For oropharyngeal tumors, HPV-positive tumors have a better outcome than HPV-negative tumors (Kimple R.J. and Harari P.M., 2015)
Patients with SCCHN in the context of minimal residual disease will be enrolled. These patients have achieved a complete response at completion of their primary treatment which will be documented on post treatment imaging performed 3 months after the end of adjuvant therapy

as per standard medical practice. The patients must be able to be given and tolerate adjuvant therapy and must recover from its toxicities.

The patients will run in two study periods:

- During a screening period for patients with locoregionally advanced, HPV-negative SCCHN who have surgery followed by standard adjuvant therapy and for whom tumor tissue and peripheral blood sample are available, an individualized neoantigen directed immunotherapy will be assembled. A 12-week period is needed to manufacture the individualized TG4050 product. During this period, patients will receive adjuvant therapy. Therefore, participating patients are not deprived of their standard of care treatment.

- A treatment period during which patients who achieved a complete response on the post treatment CT-scan or MRI receive the individualized TG4050 product.

As the vaccine will be administered after the adjuvant therapy, it may be questioned whether adjuvant therapy can impact the mechanism of action of the vaccine.
In the proposed design, samples will be collected at the time of surgery for the identification of mutations to be targeted by the vaccine. Hence, it is required that the residual cancer cells and the recurring tumor share a similar mutagenic pattern. Both chemotherapy and radiotherapy are known to induce stochastic somatic mutations and therefore it may be questioned whether this may affect the relevance of a vaccine based on the primary tumor. While the impact of chemotherapy and radiotherapy on neoantigen load is not yet fully understood, it was recently reported that mutations identified by exome sequencing in synchronous lymph node metastasis and metasynchronous recurrent lesions are in large part shared with the primary tumor with more than 90% of mutations detected in the primary tumor being passed through to the nodal metastasis or the recurrent tumor. Therefore, targets identified in the primary tumor are most likely also relevant to residual cancer cells and recurring disease. However, the same authors report that the nodal metastases and recurrent tumors also harbour newly arisen mutations; one limitation of the approach we are proposing will be that we will not be able to take in account these targets. (Hedberg M.L. et al., 2016)
Similarly, data were reported in anal squamous cell carcinoma showing that the mutational profile was minimally different in tumors pre- and post- chemoradiotherapy. (Mouw K.W. et al., 2017)
Additionally, it should be noted that radiotherapy is known to increase the presentation of neoantigens and therefore would improve efficacy of T cells induced by the vaccine. (Reits E.A. et al., 2006)

The present study will explore the feasibility, safety and immunogenicity of TG4050, a mutanome-directed active immunotherapy in HPV-negative, locoregionally advanced, SCCHN patients who underwent surgery followed by adjuvant therapy. TG4050 will be given at completion of primary treatment or at the time of recurrence in 2 groups of up to 20 patients each.

The extension of the present study into a phase I/II will allow to further accumulate safety and immunological data. Furthermore, with an adequate sample size this phase I/II trial will allow to evaluate the efficacy of TG4050 versus watchful waiting based on 2-years DFS.

1.3 TG4050 background

TG4050 is an immunotherapeutic product manufactured specifically for a given patient based on identification of tumor specific somatic mutations. Briefly, tumor specific mutations expressed in the tumor tissue are detected by comparing the tumor and healthy tissue exomes by massively parallel sequencing; evidence of expression of identified mutation is obtained through sequencing of tumor cells transcriptome (RNAseq). Subconsequently, mutated sequences are used to design a synthetic gene and cloned in a modified vaccinia Ankara viral vector (Bendjama K. and Quemeneur E., 2017).

The Modified Virus Ankara (MVA) has been generated through serial passages in chicken embryo fibroblasts. It has been used safely and effectively for small pox vaccination in more than a hundred thousand individuals. The MVA has lost the pathogenicity of its parental virus, the Chorioallantois Vaccinia virus Ankara, through alterations of its genome, i.e. 15% of genome loss. The virus is replication defective in human cells, ensuring its safety and tolerability for clinical use but replicative in avian cells. This feature enables the use of an egg-based manufacturing process. It has served as an effective vector in previous attempts to develop viral-based immunotherapies, in particular, the vector has been used for the production of several Transgene's IMPs tested in phase 1 and phase 2 clinical studies. The long track record of clinical use of the MVA demonstrate an excellent safety profile and is not associated with cardiac safety issues associated with replicative vaccinia virus (Elizaga M.L. *et al.*, 2013), (Zitzmann-Roth E.M. *et al.*, 2015).

The use of cancer specific immunogenicity has been validated through a wealth of evidence preclinically and clinically. Tumor infiltration with cytotoxic T cells has been long known as a factor of positive prognostic. More recently, molecular characterization of tumor tissues demonstrated that antigens arising from tumor specific mutations are the main targets of cytotoxic T cells (Ton N. Schumacher, Robert D. Schreiber, 2015) (Hu et al., 2018). Therapeutics stimulating the immune system has leveraged successfully activity of these cells by bypassing the propensity of tumors to escape immune control by inhibiting effector cells. Therapies such as immune checkpoint inhibitors have yielded high clinical efficacy by inhibiting pathways used by the tumor to downregulate the immune response (e.g., PD-1/PD-L1). One downside in such approach is that it remains limited to a relatively reduced fraction of patients whose immune profile is compatible with such intervention. Indeed, ICI therapy implies that there is a pre-existing immune response in patients, yet, tumors are also known to be able to inhibit priming of a cellular response by various mechanisms.

The strategy behind TG4050 is to use the natural sensitivity of the immune system to trigger a response against tumor specific antigens (TSAs), by priming the immune system outside of the tumor environment. This response may then lead to a cytotoxic activity of T cells and improvement of patient outcome. Potentially, such approach may be used synergistically with other immune modulators.

1.4 Translational research rationale

Besides the assessment of safety and tolerability, the current study is intended to provide critical data on the mechanism of action of TG4050. We aim at generating data that may help validating TG4050 as an effective tool for the development of a tumor specific immune response and obtain preliminary data on the antitumor efficacy of this individualized therapy.

Material banked after surgery will be used to identify tumor specific mutations and evaluate level of expression of tumor specific mutations. Sequencing data will be used to describe mutational load and impact of clinical covariates on the frequency of mutations.

Prior to first administration, patients will undergo apheresis in order to collect peripheral immune cells. For modalities of apheresis, please refer to section 6.2.1 (Blood sampling and apheresis). Profiling of immune response prior to treatment initiation will serve to document basal level of immune response against tumor specific neoantigens. Another collection will be performed 9 weeks (D64) after initiation of treatment to measure changes in immune response triggered by the treatment. Additionally, blood samples will be collected throughout the study to evaluate the frequency of immune cell subpopulations (T cells, NK cells, etc.) and evaluate the impact of TG4050 vaccination.

Additionally, emerging biomarkers that may be linked to disease progression will be used to monitor the effect of the product such as microRNA, circulating tumor DNA.

Immune status will also be documented by measuring cytokines known to be related to immune activation (e.g., IFNγ, IL2, IP-10, etc.)

Last, at the time of recurrence and disease progression (at disease progression for phase I part only), a tumor sample will be obtained to document the presence or absence of mutation used in the vaccine design and immune status of the disease (level of infiltration, phenotyping of infiltrates and expression of immune related factors: PD-L1, CD73, etc.), as well as alterations associated with immune escape (e.g., inactivating mutations of IFNγ signaling pathway, mutations of β2-microglobulin).

1.5 Summary of the overall benefits and risks

1.5.1 Expected benefits

TG4050 is an individualized mutanome-directed active immunotherapy designed to trigger a specific immune response against tumor specific antigens. It is expected that this specific response will induce a cytotoxic activity of T cells towards tumor cells potentially leading to an improved disease outcome.

1.5.2 Potential risks

Based on the clinical experience collected for several MVA vector-based immunotherapy products investigated by Transgene, the following related adverse events were the most common events in clinical trials:

- Injection site reactions of grade 1 and 2 based on the NCI-CTCAE version 5.0 including erythema, inflammation, pain, hemorrhage, pruritus, induration, bruising
- Lymphadenopathy
- Headache
- Pyrexia
- Fatigue
- Nausea and vomiting
- Influenza like illness

1.5.3 Measures taken to mitigate the risks

The risks-benefits relationship has been carefully considered in the planning of the study. Based on the clinical data available to date on other MVA vector-based immunotherapy products, the conduct of this study is considered justifiable using the dose and dosage regimen of TG4050 specified in the study protocol. An Independent Data Monitoring Committee (IDMC) is planned for the ongoing assessment of the risk-benefit ratio. The trial shall be discontinued in the event of any new finding that indicates a deterioration of the risk-benefit ratio and would render continuation of the trial unjustifiable.
This clinical trial will be conducted in compliance with the clinical study protocol, GCP (ICH E6 R2) and the applicable national regulatory requirements.

2. OBJECTIVES

2.1 Primary Objective

Phase I part: To evaluate the safety and tolerability of multiple subcutaneous (SC) injections of a mutanome-directed active immunotherapy (TG4050) in patients with newly diagnosed, locoregionally advanced, HPV-negative, SCCHN initiated at completion of primary treatment (arm A) or at the time of recurrence (arm B).
Phase I/II: To compare the efficacy of TG4050 (experimental arm) and watchful waiting (control arm) based on two-year Disease-Free Survival (DFS) rate in patients with newly diagnosed, locoregionally advanced, HPV-negative, SCCHN after completion of primary treatment.

2.2 Secondary objectives

Phase I part:

- To evaluate:
  - Failure to provide TG4050
  - Failure to treat with TG4050
  - Disease-Free Survival (DFS)
  - Tumor response by using RECIST 1.1 in patients with recurrent cancer
  - Progression Free Survival (PFS) in patients with recurrent cancer

Phase I/II:

- To compare the arms with respect to
  - Distant Metastases-Free Survival (DMFS)
  - Loco-Regional Relapse-Free Survival (LRFS)
  - Safety profiles
- To evaluate the Overall Response Rate (ORR) by using RECIST 1.1 in patients with recurrent disease (patients randomized to control arm and treated with TG4050 in combination with SOC)
- To evaluate the success rate in providing TG4050 for patients eligible for randomization

2.3 Translational research

Phase I part: To evaluate the following endpoints:
- Tumor mutational burden (TMB) and neoantigen frequency at Baseline and at recurrence
- Changes in circulating immune cells
- Induction of tumor specific T cells
- T cell changes in tumor immune infiltrate and environment at baseline and at recurrence
- Quantification of tumor and immune-related biomarkers

Phase I/II: To evaluate the following endpoints:
- Quantification of tumor and immune-related biomarkers including but not limited to PD-L1 expression, tumor mutational burden (TMB)
- Changes in circulating immune cells
- Induction of antigen specific T cells against neoantigens
- Changes in tumor immune infiltrate and microenvironment at baseline and at recurrence
- Circulating tumor DNA prior and during treatment / observation period

For all objectives of phase I/II, data from phase I part and phase II part will be pooled.

3. STUDY DESIGN

3.1 Overall study design and plan description

This is a multicenter open-label, 2 arms, randomized, phase I/II study evaluating TG4050, a mutanome-directed active immunotherapy based on a MVA specifically designed to target tumor specific antigens identified in each patient.
Phase I part: Patients with locoregionally advanced, HPV-negative, SCCHN who achieved a Complete Response (CR) after surgery and adjuvant therapy will be randomized in a 1:1 ratio to Arm A (treatment start at completion of primary treatment) or to Arm B (treatment start at the time of recurrence). Randomization has to be performed within 6 weeks after CR documentation. Patients will be stratified at randomization based on a dynamic method (minimization) according to prior cisplatin treatment (no versus yes), disease stage (III versus IV, based on pathological staging) and center.
Patients will receive TG4050 at $1x10^8$ PFU by subcutaneous (SC) injections weekly for the first 6 weeks and then every 3 weeks.
Arm A: Within 1 week after randomization (period may be prolonged during safety run-in phase) patients will start treatment with TG4050 as a single agent for up to a total of 20 injections or until recurrence documented clinically and by imaging or until unacceptable toxicity including occurrence of a dose-limiting toxicity or patient withdrawal for any reason, whichever occurs first. At recurrence patients will then be offered TG4050 in combination with standard of care (SOC) for up to a total of 20 injections, or until documented disease progression according to RECIST 1.1, or until unacceptable toxicity including occurrence of a dose-limiting

toxicity or patient withdrawal for any reason whichever occurs first. If unacceptable toxicity was leading to treatment discontinuation before recurrence patient will be treated with SOC only after recurrence.
Arm B: At recurrence documented clinically and by imaging, patients will receive TG4050 in combination with SOC for up to a total of 20 injections, or until documented disease progression according to RECIST 1.1, or until unacceptable toxicity including occurrence of a dose-limiting toxicity or patient withdrawal for any reason whichever occurs first.
SOC can be surgery, chemotherapy or immunotherapy at the investigator's discretion.
For both arms: at the 1st radiologic evidence of progressive disease (PD), clinically stable patients may continue TG4050 treatment at the Investigator's discretion while awaiting a confirmatory scan and / or initiation of a further antineoplastic treatment.

Phase II part: Patients with locoregionally advanced, HPV-negative, SCCHN who achieved a Complete Response (CR) after surgery and adjuvant therapy will be randomized in a 1:1 ratio to the experimental arm (treatment start at completion of primary treatment) or to the control arm (watchful waiting). Randomization has to be performed within 6 weeks after CR documentation. Patients will be stratified at randomization based on a dynamic method (minimization) according to disease stage (III versus IVA versus IVB, based on pathological staging), prior cisplatin treatment (no versus yes), localization of primary tumor (oropharynx versus oral cavity versus larynx versus hypopharynx), PD-L1 status defined by combined positivity score (CPS <1 versus CPS ≥ 1 <20 versus CPS ≥20) and center.
Experimental arm: Within 1 week after randomization patients will start treatment with TG4050 as a single agent for up to a total of 20 injections or until recurrence documented clinically and by imaging or until unacceptable toxicity including occurrence of a dose-limiting toxicity or patient withdrawal for any reason, whichever occurs first.
Control arm: Patients will undergo watchful waiting until recurrence documented clinically and by imaging. At recurrence, patients will start TG4050 in combination with SOC for up to a total of 20 injections or until documented disease progression according to RECIST 1.1, or until unacceptable toxicity including occurrence of a dose-limiting toxicity or patient withdrawal for any reason whichever occurs first. SOC can be surgery, chemotherapy or immunotherapy at the investigator's discretion.

Patients will receive TG4050 at $1x10^8$ PFU by subcutaneous (SC) injections weekly for the first 6 weeks and then every 3 weeks.

The study will consist of 2 periods:

<u>A screening period</u> starting at surgery and upon fulfilling screening period eligibility criteria in newly diagnosed patients with locoregionally advanced HPV-negative SCCHN. Locoregionally advanced SCCHN is defined as stage III or IV disease, amenable to surgery (AJCC 8th edition). During the screening period, patients will undergo standard adjuvant therapy and TG4050 manufacturing will be initiated.

Tumor tissue from primary tumor which has been banked at the time of surgery and blood samples will be tested for TSAs identification. TSAs will be used to manufacture an

individualized mutanome-directed active immunotherapy. Manufacturing of the vaccine in accordance with GMP procedures will take approximately 12 weeks.

<u>A treatment period</u> starting at randomization.
Patients will be randomized upon fulfilling treatment period eligibility criteria within 6 weeks after CR documentation.
All patients under treatment will have:

- Safety assessments performed on a weekly basis for the first 6 weeks and every 3 weeks thereafter.
- Immune responses evaluated in the blood:
  Phase I part:
    - Arm A and Arm B: before surgery, at baseline, at recurrence, at progressive disease according to RECIST 1.1, at the end of treatment visit, and during Long-Term Follow-Up as specified below (6 and 12 months after end of treatment, and then yearly up to 5 years after end of treatment)
    - Arm A: on Day 15, Day 43, Day 64, Day 85, and Day 211 before recurrence on R-Day 1, R-Day 8, R-Day 15, R-Day 43, starting from R-Day 64 every 12 weeks until progressive disease according to RECIST 1.1. Between completion of first series of 20 TG4050 administrations and recurrence every 12 weeks during the first 24 months after randomization and every 24 weeks thereafter.
    - Arm B: Between randomization and recurrence every 12 weeks during the first 24 months after randomization and every 24 weeks thereafter. After recurrence on R-Day 1, R-Day 8, R-Day 15, R-Day 43, starting from R-Day 64 every 12 weeks until progressive disease according to RECIST 1.1.

  Phase II part:
    - In experimental arm and control arm before surgery, before start of adjuvant therapy, at visit completion of adjuvant therapy, at baseline, at recurrence, at the end of treatment visit, and during Long-Term Follow-Up as specified below (6 and 12 months after end of treatment, and then yearly up to 5 years after end of treatment).
    - Experimental arm: On Day 15, Day 43, starting from Day 64 every 12 weeks during the first 24 months after randomization and every 24 weeks thereafter.
    - Control arm: On Day 15, on Day 43, starting from Day 64 every 12 weeks during the first 24 months after randomization and every 24 weeks thereafter. After recurrence on R-Day 1, R-Day 8, R-Day 15, R-Day 43, starting from R-Day 64 every 12 weeks until progressive disease according to RECIST 1.1, and at progressive disease.

- Immune responses evaluated in the tumor:
    - All patients will have immune responses evaluated in the tumor at the time of recurrence and disease progression evaluated according to RECIST 1.1 (at disease progression only in phase I part).

- Long-Term Follow-Up after completion or stop of TG4050 treatment:
    - Assessment including Clinical history (any relevant clinical events [e.g., immune disease]), a physical examination, and blood sampling for immune

response to the vaccine 6 months after end of treatment, 12 months after end of treatment and then yearly until 5 years after end of treatment

- Efficacy assessments

Phase I part:

All patients will have physical examination of the head and neck on Day 43 and then every 12 weeks during the first 24 months after randomization and every 24 weeks thereafter to detect early signs of recurrence and MRI or CT-scan of the head and neck and CT-scan of the chest in the event of clinical signs or symptoms of recurrence as per standard medical practice. After relapse, imaging procedures will be performed every 6 weeks until documented disease progression according to RECIST 1.1.

Phase II part:

All patients will have physical examination of the head and neck on Day 43 and then every 12 weeks during the first 24 months after randomization and every 24 weeks thereafter to detect early signs of recurrence and MRI or CT-scan of the head and neck and CT-scan of the chest in the event of clinical signs or symptoms of recurrence as per standard medical practice. After relapse, for patients in the control arm who have started treatment with TG4050 in combination with SOC: every 6 weeks imaging until documented disease progression according to RECIST 1.1.

A schematic illustration of the trial design is shown below.

Phase I part

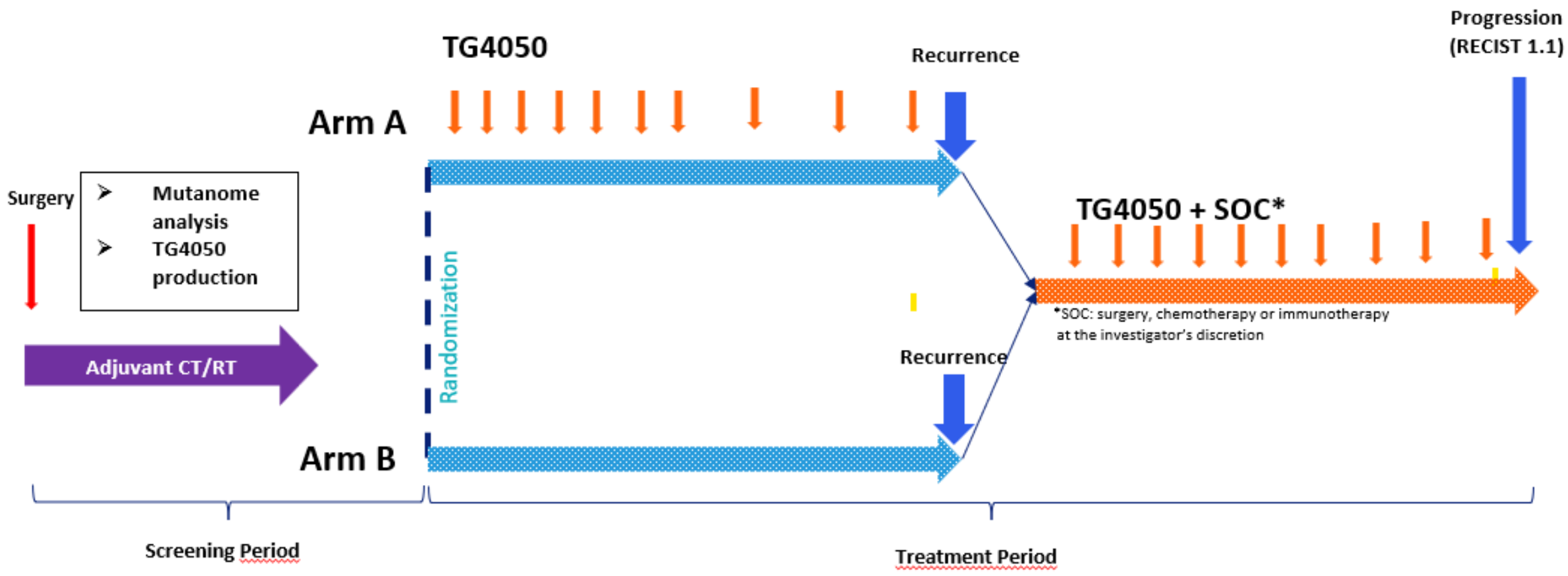

Phase II part

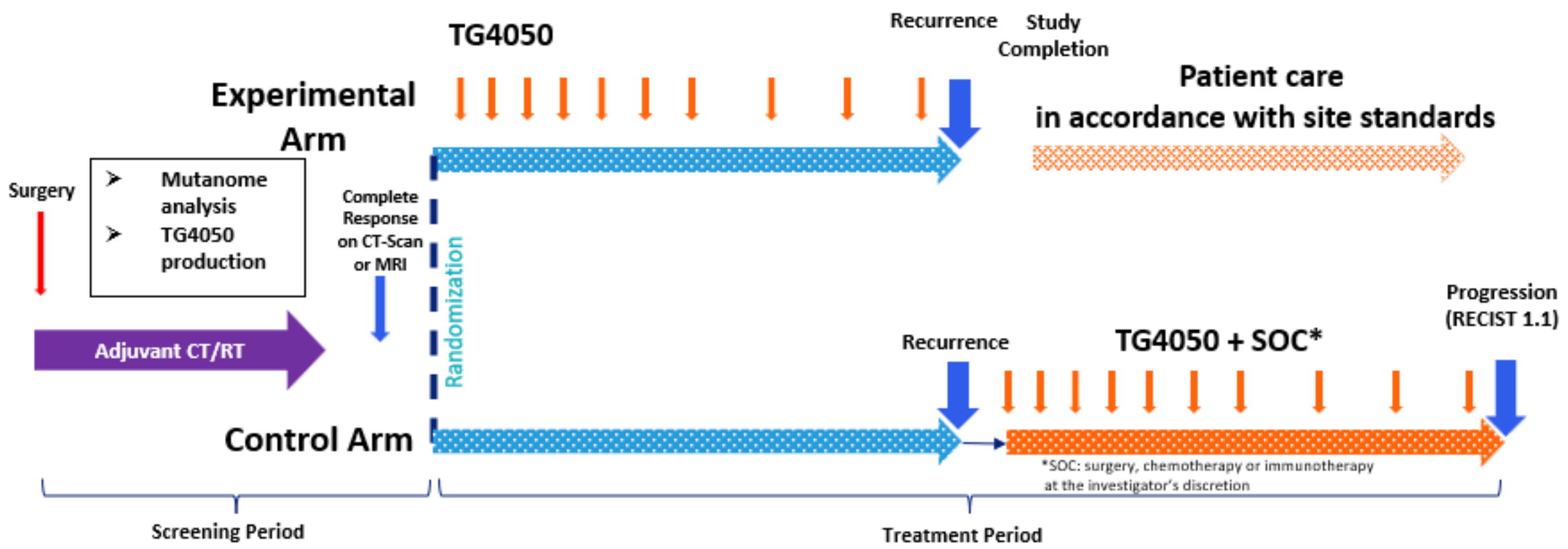

Figure 1: Overall study design

In the phase I part, a safety run-in phase will be conducted for the first 3 treated patients in each arm who will be observed during 6 weeks after the first administration of TG4050. During this phase and per arm, each patient will be monitored for 2 weeks after the first injection of TG4050 (and having received 2 injections of TG4050) before the next patient can be enrolled in the same arm.
Enrollment of the next patients will be allowed if none of the patients has experienced during the six weeks run-in phase:
- any treatment-emergent AE grade ≥3 toxicity that is at least possibly related to TG4050, with the following exception: pre-existing grade 2 fatigue at baseline that would worsen up to grade 3,
- any death.
The occurrence of any of those events will trigger a study hold. An evaluation of the cause of toxicity will be performed and corrective measures, if appropriate, will be proposed before recruitment in the study can be reinitiated.

Beyond the safety run-in phase, a dose-limiting toxicity is defined, in the phase I part and in the phase II part, as any treatment-emergent AE ≥3 toxicity that is at least possibly related to TG4050, with the following exceptions: pre-existing grade 2 fatigue at baseline that would worsen up to grade 3.

In any case, patients experiencing a dose-limiting toxicity will discontinue TG4050 treatment permanently.

An Independent Data Monitoring Committee (IDMC) will be set up for the purpose of reviewing patient safety data and the conduct of the study.

For the phase I part, the IDMC will review the safety of the first 3 treated patients in Arm A and of the first 3 treated patients in Arm B who have completed their first 6 weeks of TG4050 treatment or have discontinued the study treatment due to treatment-related toxicity. The IDMC will assess/recommend whether it is possible to pursue patient enrolment as per study protocol. In addition to the reviews of the first 3 patients treated in Arm A and Arm B, respectively the IDMC will review safety data of all patients included every 3 months during the first year and every 6 months thereafter. With the initiation of the phase II part, safety data of patients in the phase I part will be continued to be reviewed at the schedule for phase II part.
Ad hoc meetings may be held upon necessity.

For the phase II part, the IDMC will review safety data of all patients included every 12 months and at final analysis.
Ad hoc meetings may be held upon necessity.

3.2 Number of centers and patients

The study will be a multicenter trial with approximately 15 clinical sites in France, UK, Spain and US.

Phase I part: An approximate number of 80 patients will enter the screening period assuming that at least 50 % of patients will be unfit for receiving adjuvant therapy or will not achieve a CR.
A total of up to 40 patients will be randomized into treatment period, up to 20 in each study arm.

Phase II part: An approximate number of 90 to 100 patients will enter the screening period assuming that at least 50 % of patients will be unfit for receiving adjuvant therapy or will not achieve a CR.
A total of at least 46 patients will be randomized to obtain at least 42 evaluable patients.

3.3 Discussion of Study Design

3.3.1 Phase I part

The first-in-man study of TG4050 is designed in its phase I part to primarily evaluate the safety of TG4050 in stage III or IV, HPV negative SCCHN patients who are in complete response after completion of primary treatment consisting of surgery and adjuvant therapy and who start treatment with TG4050 alone immediately (Arm A) or in combination with SOC at the time of recurrence (Arm B). Immunological analyses will be performed to evaluate the induction of immune stimulation by TG4050.

Patients from both arms will be regularly followed by physical examination of the head and neck in order to detect early signs of recurrence and by MRI or CT-scan in the event of clinical signs or symptoms. DFS will be determined in arm A and arm B. Once recurrence has been documented, tumor response by using RECIST 1.1 will be assessed every 6 weeks in both arms.

3.3.1.1 Phase I part - Safety

Between December 2019 and August 2022, a total of 80 patients have been consented for participation into the phase I part of the present study. Thirty-three (33) patients have been randomized, 17 patients to Arm A with immediate start of TG4050 treatment, and 16 patients to Arm B with treatment start at recurrence, respectively.

Main reasons for screen failure were early relapse (15 patients, 31.9%) and withdrawal of consent (14 patients, 29.8%). Only 3 patients (6.4%) could not be randomized due to failure in providing individualized IMP.

As of 10 May 2023, cut-off date for a communication at ASCO 2023 (Ottensmeier C. *et al.*, 2023) a total of 18 patients have initiated treatment with TG4050, all patients in Arm A (N=17) and 1 patient in Arm B with a median number of 17 administrations.
Safety data from these 18 patients show that TG4050 is well tolerated. A total of 77 Adverse Reactions (ARs) have been reported in 17 patients (94%). All ARs were of mild or moderate intensity with no grade ≥ 3. The most frequently reported ARs were reactions at the injection site in 15 patients (83%, 57 events) with injection site reactions not otherwise specified (35%, 18 events in 6 patients), injection site inflammation (28%, 16 events in 5 patients), injection site oedema (28%, 5 events in 5 patients), injection site pain (28%, 5 events in 5 patients), and injection site bruising (22%, 4 events in 4 patients).

During the conduct of the phase I part the IDMC has reviewed at intervals outlined in the present protocol the overall study progress as well as all patient’s safety data. No safety concern was raised during any of these reviews.

3.3.1.2 Phase I part - Evaluation of immune responses to TG4050 treatment

Immune responses were evaluated by assessing antigen-specific T cell responses and tumor infiltrates as well as changes in phenotype of immune or tumor cells in 8 patients treated with TG4050 and analyzed at cut-off date.

- Phenotyping of circulating immune cells

PBMCs were analyzed from samples collected at baseline and 9 weeks after TG4050 treatment initiation (i.e., Day 64 and following 7 weekly administrations of TG4050).
There were no striking changes in the absolute counts of cell populations but an increase in the percentage of effector cells between baseline and Day 64.

- Adaptive T-cells responses against targeted neoepitopes assessment

T-cell responses for each targeted mutation were assessed 9 weeks after TG4050 treatment initiation meaning (i.e., Day 64 and following 7 weekly administrations of TG4050) and compared to baseline for evaluable patients.
Neoepitope immunoreactive T-cells were quantified in PBMCs against each of the targeted epitopes using an *ex vivo* ELISPOT (without pre-expansion). All tested patients developed a polyepitopic T cell response against vaccine targets with a mean of 9 positive responses for each patient. These results confirm the ability of TG4050 to effectively prime the patient immune system and develop robust T cell responses against tumor specific antigens.

- Characteristics of Tumor Microenvironment (TME) and tumor immunoprofiling

Tumor samples collected at the time of surgery were analyzed for tumor immune infiltrates and expression of factors related to response to immunotherapy such as TMB or PD-L1 expression.
All patients developed a polyepitopic response regardless of HLA and TME immune features.
Safety observed in the phase I part as well as first signals of immune stimulation warrant investigation for clinical efficacy of TG4050 in a larger number of patients.

3.3.2 Extension to Phase I/II

The extension into a phase I/II will allow to further accumulate safety data and immunological data. With an adequate sample size, this phase I/II trial will allow to compare the efficacy of TG4050 and watchful waiting based on two-year Disease-Free Survival (DFS) rate.
Patients with locoregionally advanced, HPV-negative, SCCHN who achieved a Complete Response (CR) after surgery and adjuvant therapy will be randomized in a 1:1 ratio to the experimental arm (treatment start at completion of primary treatment) or to the control arm (watchful waiting).
In the experimental arm patients will start treatment with TG4050 as a single agent for up to a total of 20 injections or until recurrence documented clinically and by imaging or until unacceptable toxicity including occurrence of a dose-limiting toxicity or patient withdrawal for any reason, whichever occurs first. In the control arm patients will undergo watchful waiting until recurrence documented clinically and by imaging. At recurrence, patients will start TG4050 in combination with SOC for up to a total of 20 injections or until documented disease progression according to RECIST 1.1, or until unacceptable toxicity including occurrence of a dose-limiting toxicity or patient withdrawal for any reason whichever occurs first.

3.4 Dose and schedule selection

Dose and dosing schedule for TG4050 administration have been selected on the basis on the broad clinical experience gathered on TG4010, an MVA carrying coding sequences for the human MUC1 antigen and human interleukin 2 also developed by the sponsor. As of end 2018 a total of 354 cancer patients, mostly non-small cell lung cancer (NSCLC) patients, received SC injections of TG4010 at a dose of 1 x 10^8 PFU weekly for 6 weeks followed by every 3 weeks administrations.

• Rationale for the route of administration:

In pre-clinical experiments, SC and IM administrations of research grade TG4010 resulted in similar anti-tumoral activity as measured by survival in the RMA-MUC1 prophylactic mouse model. This finding was repeated with the other Transgene MVA-based gene therapy products. The SC route has been chosen from phase II studies of TG4010 and other MVA-based products as it appears a more effective route of administration to initiate an immune reaction which is the expected Mechanism of Action for Transgene MVA-based products. It has been demonstrated as a safe and immunogenic route in the clinic with MVA-based products.

• Rationale for the dose and regimen:

In TG4010 development, phase II clinical trials were conducted testing different schedules of administration of the study drug.
The dose of $1x10^8$ PFU was selected based on its good tolerance and immunogenicity. With its design targeting patient specific neoantigens, TG4050 products should target more immunogenic antigens than the ones targeted by MVA-based products already tested in clinic by Transgene. It is envisaged that a critical dose threshold needs to be reached to achieve an immunogenic response disregarding the nature and immunogenicity of the patient specific antigens carried by the MVA vector. This was consolidated by a pre-clinical experiment in HLA-A2 mice who received different doses of MVA constructs representative of TG4050 and showed response with no dose-response effect.
Sticking with a dose of $1x10^8$ PFU which allowed immunogenic response with an MVA-based product carrying a weak antigen sequence (i.e.: MUC1 antigen for TG4010) should then be relevant for TG4050. The high specificity of TG4050 antigens to the human tumor should prevent from autoimmune reaction.
In prostate cancer patients the change in PSA doubling time from before treatment to during treatment was statistically significant in patients who received TG4010 weekly for 6 weeks then every 3 weeks while those change was not significant in those who received TG4010 every 3 weeks. Therefore, this treatment regimen was used in all subsequent studies.

• Rationale for repeated administrations scheme:

Therapeutic vaccines do not directly target the tumor but they rather aim at activating and amplifying the immune reactions against the tumor. Immune responses often take time to develop and can potentially be enhanced by continued booster vaccinations. Then a repeated schedule of administration was selected for TG4010 as well as the other Transgene MVA-based products and is also applied to TG4050 in the clinic. This repeated dose study regimen was shown to be well tolerated, grade 1 or 2 injection site reactions being the most frequent Adverse Events (AEs). Importantly this regimen of TG4010 in combination with standard chemotherapy was shown to improve patient outcome in a randomized placebo-controlled, double-blind phase 2 study comparing the combination with chemotherapy alone in 222 first-line NSCLC patients (Quoix E. *et al.,* 2016). Consistent with repeated injections of TG4010, development of CD8+ T cell response to MVA-specific epitopes were observed at different timepoints and more frequently in the TG4010 arm in comparison to the placebo arm in this phase 2 clinical trial (Tosch C. *et al.,* 2017). Interest of repeated administrations was also evidenced with MVA-based products not developed by Transgene. For instance, CD4+ and CD8+ T-cell responses to

MVA or Dryvax were reported in subjects vaccinated with TBC-MVA and responses were augmented after three doses of MVA compared to one dose. (Parrino J. et al., 2007).
From an immunologic perspective, there is a theoretical possibility of T cell cross-reactivity with repeated administrations of TG4050. This potential risk was previously monitored in TG4010 clinical trials assessing repeated administrations of $1x10^8$ PFU through auto-immunity parameters assessment (e.g. anti-DNA antibodies, anti-nuclear antibodies, thyroid stimulating hormone, anti-thyroperoxidase antibodies). While some of these antibodies increased slightly during study compared to pre-treatment level, it was never considered clinically significant by the investigators. There was also no clinical evidence of auto-immunity to glandular mucins reported (Sjogren's syndrome). No further autoimmunity clinical issue is expected with TG4050 as it targets mutations of patient specific tumor antigen and it has a different mechanism of action from checkpoint blockade.

In Arm A in the phase I part and in the experimental arm in the phase II part, TG4050 will be administered upon completion of primary treatment at $1x10^8$ PFU by subcutaneous (SC) injections weekly for the first 6 weeks and then every 3 weeks for up to a total of 20 injections or until recurrence documented clinically and by imaging or until unacceptable toxicity including occurrence of a dose-limiting toxicity or patient withdrawal for any reason whichever occurs first . The same schedule of administrations is followed after recurrence in Arm A and Arm B in the phase I part and in the control arm in the phase II part, i.e. for up to a total of 20 injections or until documented disease progression according to RECIST 1.1 or until unacceptable toxicity including occurrence of a dose-limiting toxicity or patient withdrawal for any reason whichever occurs first. Overall, the good tolerance and the efficacy of the proposed TG4050 regimen have been established for an MVA-based product developed by the sponsor.

• Rationale for a second course of TG4050 treatment in Arm A in the phase I part:

TG4050 treatment is initiated after documentation of complete response either at completion of primary treatment (Arm A) or at the time of recurrence (Arm B). The TG4050 treatment is a vaccine monotherapy in the context of a minimal residual disease (MRD) in the former case, and part of a combination therapy with standard of care (surgery, chemotherapy or immunotherapy) in the latter.
In an MRD context, the disease is undetectable by imaging and is mainly characterized by scattered tumor cells. As such these cells are ideal target for immunotherapy: as isolated cells or small size aggregates, tumor cells are readily accessible to the immune system and subject to cytotoxic effect from T cells or innate immunity cells.
On the contrary, at the time of recurrence, tumor growth is associated with the onset of tumor defense mechanisms against the immune system by the promotion of an immunosuppressive tumor microenvironment. There is growing evidence that agents used for the treatment of relapsing SCCHN (platinum salts potentially completed with anti-PD1 immunotherapy, taxanes, fluorouracil) are likely to counter tumor induced immunosuppression (Weir G.M. *et al.,* 2011). Mechanisms are complex and may involve destruction of tumor induced immune suppressive cells such as myeloid-derived suppressor cells (MDSC), upregulation of MHC expression by the tumor and or direct stimulation of effector response by T cells (Zitvogel L. *et al.,* 2008).
By proposing vaccination to the patients in Arm A both alone and in combination with other agents, we intend to obtain data on:

- the relative sensitivity to vaccination of cancer initiating cells and established recurrence

- whether early treatment with the vaccine will affect biological activity of the vaccine at recurrence
- the relative importance of a chemotherapy or anti-PD1 to be given together with the vaccine

At this stage, we have not obtained data from the phase I part that support the administration of a second cycle of vaccination after recurrence. Therefore, at this stage we do not intend to implement this second cycle of vaccination in the phase II part until we can ascertain that recurrence is not associated with immunoediting of vaccine targeted clones.

4. STUDY POPULATION

4.1 Inclusion criteria

<u>Screening period</u>

1. Signed written informed consent in accordance to ICH-GCP and national/local regulation before undertaking any protocol-specific procedures
2. Newly diagnosed stage III or IV amenable to surgery (AJCC 8th edition), squamous-cell carcinoma of the oral cavity, the oropharynx, hypopharynx or larynx excluding HPV-positive primaries
3. Patients must have undergone gross total resection of the primary tumor
4. Patients must be eligible for adjuvant therapy
5. a) Tumor tissue from primary tumor obtained during surgery and peripheral blood sample must be available for exome and transcriptome sequencing
   b) For phase II part tumor tissue from surgery must be available for centralized testing of PD-L1 using the 22C3 clone
6. Female or male patients, aged at least 18 years

<u>Treatment period</u>:

7. Patients must have completed adjuvant radiotherapy +/- chemotherapy and have a post-treatment CT or MRI of the head and neck and CT-scan of the chest documenting CR. Imaging has to be performed 3 months after completion of adjuvant radiotherapy +/- chemotherapy and within 6 weeks prior to randomization
8. Phase I part: Patients must have recovered from the toxicities of adjuvant therapy
   Phase II part: Patients must have recovered from toxicities of adjuvant radiotherapy +/- chemotherapy to ≤ Grade 1. Patients with ≤ Grade 2 neuropathy, dysgeusia, dysphagia, and xerostomia are eligible
9. Eastern Cooperative Oncology Group (ECOG) performance status (PS) of 0 or 1
10. Adequate hematological, hepatic and renal functions:
    - Hemoglobin ≥ 9.0 g/dL (without packed red blood cell transfusion within the prior 3 weeks)
    - Neutrophils count ≥ 1.5 x10^9/L
    - Total lymphocytes count ≥ 0.5 x10^9/L
    - Platelets count ≥ 100 x10^9/L

- Total bilirubin ≤ 1.5 x ULN (except for patients with Gilbert's syndrome who may be included if total bilirubin ≤ 2.5 x ULN)
- Serum transaminases: alanine aminotransferase [ALT] and aspartate aminotransferase [AST]) ≤ 2.5 x ULN
- Calculated creatinine clearance ≥ 40 mL/min using the Cockroft & Gault formula
- Serum albumin ≥ 30 g/L

11. No locoregional relapse or occurrence of distant metastases within 6 weeks prior to randomization
12. No active infection. For phase II part, this includes infection with mpox and SARS-CoV-2 (COVID-19) PCR positive testing within 30 days prior to randomization
13. Highly Effective contraception (i.e. methods with a failure rate of less than 1% per year) during the study period and for 3 months after the last study treatment administration for female patients of childbearing potential (WOCBP) and for male patients who are sexually active with WOCBP (See Appendix II for WOCBP definition and details on highly effective contraception methods). Highly effective contraception methods are defined as:
    • Hormonal methods of contraception including combined oral contraceptive pills, vaginal ring, injectables, implants, intrauterine devices (IUDs) such as Mirena and Nonhormonal IUDs such as ParaGard for WOCBP patient or male patient's WOCBP partner
    • Tubal ligation
    • Vasectomy
    In addition to highly effective contraception, participating male patients must use a condom during the study period and for 3 months after the last study treatment administration.
14. Negative blood pregnancy test for female patients of childbearing potential (Appendix II)

4.2 Exclusion criteria

<u>Screening period</u>

1. a) Patients with carcinoma of the nasopharynx, squamous cell-carcinoma of unknown primary, squamous cell carcinoma that originates from the skin and salivary gland or paranasal sinus, non-squamous histologies
   b) Patients with stage I or II based on pathological staging
2. a) Prior exposure to anti-cancer vaccines, and any antibody targeting T-cell co-regulatory proteins such as anti-PD1, anti-PDL1 or anti-CTLA4 antibodies
   b) Prior radiotherapy or systemic anti-cancer therapy including investigational agents for the SCCHN under study (for phase II part)
3. Other active malignancy requiring concurrent systemic intervention
4. Patients with previous malignancies other than the target malignancy to be investigated in this trial (except non-melanoma skin cancers, early stage resected papillary thyroid cancer, and the following in situ cancers: bladder, gastric, colon, endometrial, cervical/dysplasia, melanoma, or breast) are excluded unless a complete

remission was achieved at least 2 years prior to study entry. Additional therapy must not be planned or required during the study period

5. Patients with any organ transplantation, including allogeneic stem cell or bone marrow transplantation
6. Any known allergy or reaction to eggs or attributed to compounds of similar chemical or biological composition to therapeutic vaccines/immunotherapeutic products
7. Known history of positive testing for Human Immunodeficiency Virus (HIV) or known Acquired Immune Deficiency Syndrome (AIDS)
8. Clinical or laboratory history or evidence of Hepatitis C Virus (HCV) or Hepatitis B Virus (HBV) indicating acute or chronic infection
9. Any psychological, familiar, sociological or geographical condition potentially hampering compliance with the study protocol and follow-up schedule; those conditions should be discussed with the patient before registration in the trial
10. Pregnant or nursing (lactating) women, where pregnancy is defined as the state of a female after conception and until the termination of gestation, confirmed by a positive hCG laboratory test (> 10 mIU/mL)

Treatment period

11. Patients under chronic treatment with systemic corticosteroids or other immunosuppressive drugs for a period of at least 4 weeks and whose treatment was not stopped 2 weeks prior to randomization, with the exception of patients with adrenal insufficiency who may continue corticosteroids at physiological replacement dose, equivalent to ≤ 10 mg prednisone daily. Steroids with no or minimal systemic effect (topical, inhalation) are allowed
12. Phase I part: Vaccination with a live vaccine for the prevention of infectious diseases in the four-week period prior to randomization
    Phase II part: Vaccination for the prevention of infectious diseases in the four-week period prior to randomization with a live vaccine or any COVID-19-vaccine
13. Treatment with another investigational agent since the beginning of the screening period
14. Uncontrolled intercurrent illness including but not limited to ongoing or active infection, symptomatic congestive heart failure, unstable angina pectoris, cardiac arrhythmia or psychiatric illness/social circumstances that could limit compliance with study requirements

4.3 Criteria for patient premature withdrawal

4.3.1 Premature withdrawal from the trial

Patients are free to discontinue the trial at any time without giving reasons. Withdrawal of consent will be considered withdrawal from the trial unless the patient agrees to be followed until documentation of recurrence or progressive disease which may include verification of medical records.
A patient must be withdrawn in the event of any of the following:

- Withdrawal of the patient’s consent
- Participation in any other trial during the duration of this trial
- Lost to follow up

4.3.2 Withdrawal from study treatment

Patients must discontinue the study treatment if any of the following occurs:

- Significant clinical deterioration (clinical progression), defined as new symptoms that are deemed by the investigator to be clinically significant. However, every effort should be made to document objective progression even after discontinuation of treatment
- Occurrence of a dose-limiting toxicity i.e any treatment-emergent AE grade ≥3 toxicity that is at least possibly related to TG4050, with the following exception: pre-existing grade 2 fatigue at baseline that would worsen up to grade 3
- If a patient becomes pregnant at any time after she has received her first TG4050 injection.
- Physician’s decision that further participation in the study is not in the patient’s best interest
- Patient’s request at any time for any reason
- TG4050 expiry date is reached
- Protocol violation
- Lost to follow up

For any discontinuation, the Investigator will obtain all the required details and document the date and the reason for the discontinuation in the eCRF. For any treatment discontinuation not related to relapse (Arm A in the phase I part and experimental arm in the phase II part) or to disease progression (both arms in the phase I part and control arm in the phase II part) the patient will be followed per protocol until documentation of relapse or disease progression, respectively.
The shelf life of TG4050 is 60 months. Considering the planned duration of treatment being around 11 months, patients need to start treatment around 11 months before expiry date, applicable after relapse for all patients in phase I and the control arm in phase II. If the reason of stopping the treatment is an AE, the specific event will be recorded in the eCRF. The Investigator will make thorough efforts to document the outcome.
As far as possible, no patient should leave the study without having undergone the end of treatment visit and the safety follow-up visit.

4.3.3 Replacement policy

In the safety run in phase part of the trial (3 first patients per arm), only patients who do not complete the observation period consisting of the first 6 weeks of study treatment for reasons other than treatment-related toxicities or who are not evaluable for safety analysis will be replaced. In the phase I/II, patients who are not evaluable for efficacy analyses will be replaced to have at least 74 evaluable patients. Evaluable patients’ populations are defined in section 9.1.

4.4 Definition of end of study

The end of study is defined as the date of the last patient last visit (LPLV).

5. REGISTRATION PROCEDURES

The study design will require two registration procedures, one for the screening period and the other for the treatment period.

5.1 Screening period

Before surgery, the patient will sign an informed consent form (ICF) and will be assigned a 15-digit identification number which is a combination of the study code (TG4050-02), the 4-digit center number and a 3-digit patient number. The center number is assigned by the sponsor to the investigational site. At each site, the first patient screened is assigned patient number 001 and subsequent patients are assigned consecutive numbers. Once assigned to a patient the patient number will not be reused.
Screening period starts at surgery. During the screening period, patients will undergo standard adjuvant therapy and TG4050 will be manufactured.

If the patient is not included for any reason, the reason for non-inclusion and a minimum of other parameters (date of ICF signature, demographics and history of SCCHN) will be entered in the eCRF.

5.2 Treatment period

5.2.1 Baseline assessment

Patients eligible at the time of post-adjuvant therapy imaging and for whom TG4050 is available will start baseline assessment. The patient will keep the same identification number for the treatment period. The baseline assessment will last at most 21 days. Tests performed per standard of care (e.g., cervical imaging) may not need to be repeated if collected within 21 days prior to randomization.
If the patient is not included for any reason, the reason for non-inclusion and a minimum of other parameters (results of the baseline assessments) will be entered in the eCRF.

5.2.2 Randomization

Phase I part: Patients fulfilling treatment period eligibility criteria will be randomized to receive TG4050 immediately (arm A) or at the time of recurrence (arm B) in a 1:1 ratio.
Randomization has to be performed within 6 weeks after CR documentation. Patients will be stratified at randomization based on a dynamic method (minimization) according to prior cisplatin treatment (no versus yes), disease stage (III versus IV, based on pathological staging) and center.
Phase II part: Patients fulfilling treatment period eligibility criteria will be randomized in a 1:1 ratio to the experimental arm (treatment start at completion of primary treatment) or to the control arm (watchful waiting).
Randomization has to be performed within 6 weeks after CR documentation. Patients will be stratified at randomization based on a dynamic method (minimization) according to disease stage (III versus IVA versus IVB, based on pathological staging), prior cisplatin treatment (no versus yes), localization of primary tumor (oropharynx versus oral cavity versus larynx versus hypopharynx), PD-L1 status defined by combined positivity score (CPS <1 versus CPS ≥1 <20 versus CPS ≥20) and center.

6. STUDY VISITS AND PROCEDURES

A flow chart in Appendix III summarizes the evaluations to be performed along with the timepoints for each period of the study and the data to be collected in the eCRF.

6.1 Screening period

6.1.1 Evaluation description

- **Inclusion/exclusion criteria**
  Patient eligibility is to be established by the investigator by confirming that all the screening period inclusion / exclusion criteria are met. Deviation from any eligibility criterion excludes a patient from registration into the screening period.

- **Demography**
  - Date of birth, gender
  - Childbearing potential status
  - Smoking habits and Alcohol consumption

- **History of SCCHN**
  - Date of diagnosis and surgery
  - Site of primary tumor
  - Stage at diagnosis
  - Histology type
  - Pathological staging on resected tumor
  - HPV status
  - PD-L1 CPS (phase II part only)

- **Relevant medical history**

- **Clinical examination**
  - Physical examination of Head and Neck including panendoscopy if appropriate, MRI or CT of the head and neck in the event of clinical signs or symptoms of recurrence as per standard medical practice
  - Physical examination of the major organ systems, including vital signs (body temperature, pulse rate and blood pressure), weight and height (only at visit at completion of surgery)

- **Collection of concomitant medications and significant non-drug therapies**

- **Blood sampling**
  - Blood sampling for ctDNA analysis before surgery, before start of adjuvant therapy, and at visit "Completion of adjuvant therapy" (phase II part only)
  - Blood sampling needed for generation of reference exome
  - HCV serology and detection of HBs antigen, if applicable
  - Serum pregnancy test (for women of childbearing potential [see definition in Appendix II])

- **Tumor tissue availability**

- Tumor tissue obtained at diagnostic biopsy, if available (phase II part only)
- Tumor tissue from primary tumor obtained at the time of surgery will be used for TSAs identification

- **Tumor imaging**
  - CT or MRI of the head and neck and CT-scan of the chest after surgery and 3 months after completion of adjuvant therapy

6.1.2 Before surgery

- Tumor material collection from diagnostic biopsy, if available (phase II part only)
- Blood sampling for gene sequencing and ctDNA analysis

6.1.3 Visit at completion of surgery

- After surgery: Inclusion / exclusion criteria check and recording of demography, history of SCCHN and relevant medical history
- Tumor tissue sample for sequencing and banking
- Physical examination of major organ systems including vital signs (body temperature, pulse rate and blood pressure), weight and height (if height is not done at this visit it can be measured at any other visit during screening period and reported under this visit without specification of date of measurement)
- HCV serology and detection of HBs antigen if applicable
- Pregnancy test, if applicable and if not already done before surgery
- Concomitant medications
- Tumor assessment by imaging procedures

6.1.4 Visit before start of adjuvant therapy (phase II part only)

- Blood sampling for ctDNA analysis

6.1.5 Visit post adjuvant therapy (3 months after adjuvant therapy completion)

- The following parameters will be recorded: Total radiation dose and fractionation regimen
- Number of cycles and cumulative dose of cisplatin, if applicable
- CT or MRI of the head and neck and CT-scan of the chest to confirm complete locoregional response and absence of distant metastases (3 months after completion of adjuvant therapy)
- Concomitant medications
- Physical examination of major organ systems including vital signs (body temperature, pulse rate and blood pressure) and weight
- Blood sampling for ctDNA analysis (phase II part only)

6.2 Treatment period

Eligible patients for whom the individualized TG4050 construct is available will be entered in the treatment period and randomized within 6 weeks after complete response documentation.
Patients not eligible or for whom the individualized TG4050 product is unavailable will continue to beneficiate from routine medical monitoring and will receive SOC in case of recurrence as per normal practice.

6.2.1 Evaluation description

The following parameters will be measured during this period:

- **Inclusion/exclusion criteria**
  Patient eligibility is to be established by the investigator by confirming that all the treatment period inclusion / exclusion criteria are met. Inclusion and exclusion criteria of the screening period must as well be re-verified and met. Deviation from any eligibility criterion excludes a patient from registration into the treatment period.

- **Current medical conditions**
  - Syndrome/pathology with established diagnosis present during the screening period either recovered or ongoing at Baseline visit
  - "Signs and symptoms" for symptoms with no associated syndrome (e.g., diarrhea) see section 8.2.2.2

- **Clinical examination**
  - Physical examination of Head and Neck including panendoscopy if appropriate, MRI or CT of the head and neck in the event of clinical signs or symptoms of recurrence as per standard medical practice
  - Physical examination of the major organ systems, including vital signs (body temperature, pulse rate and blood pressure), and weight
  - PS on ECOG scale (see APPENDIX I)

- **Concomitant medications and significant non-drug therapies**

- **Electrocardiogram (12 leads)**

- **Safety assessments**
  - AEs/SAEs collection see section 8.2.2.2
  - Follow-up of all ongoing AEs

- **Laboratory assessments**
  - CBC including Red Blood cells (RBC), White Blood cells (WBC) and differential, Hemoglobin (Hb), and platelets
  - Biochemistry including AST, ALT, alkaline phosphatases, LDH, total bilirubin, serum protein, serum albumin, glucose, electrolytes (sodium, potassium, calcium, magnesium and bicarbonate) creatinine and creatinine clearance (calculated)

  $$\text{Creatinine clearance for males (mL/min)} = \frac{(140 - \text{age}) \times (\text{weight in kg}) \times (1.23)}{(\text{serum creatinine in µmol/L})}$$

$$\text{Creatinine clearance for females (mL/min)} = \frac{(140 - \text{age}) \times (\text{weight in kg}) \times (1.04)}{(\text{serum creatinine in µmol/L})}$$

- Serum pregnancy test (for women of childbearing potential [see definition in Appendix II])

- **Blood sampling and apheresis** for translational research, if not clinically feasible, the apheresis procedure can be replaced by a 150 ml blood draw. Apheresis in phase II part only in selected centers, in centers not selected for apheresis procedure, a 150 ml blood draw is mandatory at Baseline and after 9 weeks of treatment, corresponding to Day 64 in Arm A (phase I part) and Experimental Arm (phase II part) and to R-Day 64 in Arm B (phase I part) and Control Arm (phase II part), respectively

- **Tumor sampling**

- **Skin biopsy at the site of injection in patients with injection site reaction (phase I part only)**

- **Tumor imaging:**
  - Before recurrence in the event of clinical signs or symptoms: CT or MRI of the head and neck and CT-scan of the chest are mandatory
  - After recurrence, tumor assessment will be performed every 6 weeks with a time window of +/- 7 days from start of treatment until documented disease progression according to RECIST 1.1 (both arms in the phase I part, control arm in the phase II part)

6.2.2 Baseline visit

The following procedures will be performed within 21 days prior to randomization:

- Inclusion/exclusion criteria check
- Physical examination of Head and Neck including panendoscopy, if appropriate
- Physical examination of the major organ systems, including vital signs (body temperature, pulse rate and blood pressure), and weight
- PS on ECOG scale
- Relevant medical conditions and signs/symptoms (for signs and symptoms: present within 21 days prior to first TG4050 administration), see section 8.2.2.2
- Concomitant medications
- Electrocardiogram
- AE/SAEs collection see section 8.2.2.2
- Tumor assessment by imaging procedures if the previous imaging has been performed more than 3 weeks prior to randomization

The following procedures must be performed within 7 days prior to randomization:

- Inclusion/exclusion criteria check
- AE/SAEs collection see section 8.2.2.2
- Blood analyses including:

- CBC
- Biochemistry
- Serum pregnancy test (for women of childbearing potential [see definition in Appendix II])
- Blood sample and apheresis for translational research, if not clinically feasible, the apheresis procedure can be replaced by a 150 ml blood draw. In the phase II part, apheresis will be done only in selected centers. In centers not selected for apheresis procedure, a 150 ml blood draw is mandatory

Eligible patients will be randomized by using the eCRF. The eCRF will randomly assign the patient to one of the study arms (arm A or arm B in the phase I part, experimental arm or control arm in the phase II part).

6.2.3 Study visits during the treatment period in the Phase I part

Arm A:

Eligible patients randomized to arm A will start treatment with TG4050 within 1 week after randomization (period may be prolonged during safety run-in period).

From Day 1 to Day 43

On a weekly basis:
- Physical examination of the major organ systems, including vital signs (body temperature, pulse rate and blood pressure), and weight
- PS on ECOG scale
- Adverse events reporting
- Concomitant medications
- CBC (starting on D8)
- Biochemistry (starting on D8)

On an every three-weeks basis:
- Electrocardiogram (Days 22 and 43)
- Serum pregnancy test (Days 22 and 43 for women of childbearing potential [see definition in Appendix II])

On Day 43 physical examination of Head and Neck including panendoscopy if appropriate, MRI or CT of the head and neck in the event of clinical signs or symptoms of recurrence as per standard medical practice.

For translational research:
- Blood samples on days 15 and 43
- Skin biopsy on days 8 and 43 at the site of previous injection if injection site reactions have been observed

Beyond D43

On an every three-weeks basis:

- Physical examination of the major organ systems including vital signs (body temperature, pulse rate and blood pressure), and weight
- PS on ECOG scale
- Adverse events reporting
- Concomitant medications
- Serum pregnancy test (for women of childbearing potential [see definition in Appendix II])
- CBC & Biochemistry every 3 weeks up to Month 6 (Day 190), thereafter every 12 weeks

ECG every 6 weeks up to Month 6 (Day 190), thereafter every 12 weeks

Physical examination of Head and Neck including panendoscopy if appropriate, MRI or CT of the head and neck in the event of clinical signs or symptoms of recurrence as per standard medical practice every 12 weeks during the first 24 months after randomization and every 24 weeks thereafter.

For translational research:

- Blood samples on days 64, 85 and 211
- Apheresis on day 64, if not clinically feasible, the apheresis procedure can be replaced by a 150 ml blood draw (apheresis only during first series of TG4050 treatment)
- Tumor sample at the time of recurrence
  A histological/cytological confirmation of recurrence is required

<u>Completion of first series of 20 TG4050 administrations</u> (30 days after last administration of first series of TG4050 and if the patient did not relapse, did not experience an unacceptable toxicity including dose-limiting toxicity or did not withdraw for any reason):

- Adverse events reporting
- Concomitant medications
- Serum pregnancy test (for women of childbearing potential [see definition in Appendix II])

The time windows allowed for the study visits **during treatment** are:

- +/- 1 day from Day 1 to Day 43
- +/- 7 days beyond Day 43

Between completion of first series of 20 TG4050 administrations and recurrence patients are followed as per standard medical practice (every 12 weeks with a physical examination of the head and neck including panendoscopy if appropriate, MRI or CT of the head and neck in the event of clinical signs or symptoms of recurrence during the first 24 months after randomization and every 24 weeks thereafter). Blood sampling for translational analysis is performed every 12 weeks during the first 24 months after randomization (two timepoints being 18 months and 24 months after start of TG4050 treatment) and every 24 weeks thereafter until recurrence.

The time windows allowed for physical examination of the head and neck **after treatment completion** are:

- +/-1 week

The time windows allowed for blood sampling after **treatment completion** are:

- +/- 4 weeks

<u>At recurrence</u>

- Physical examination of the major organ systems including vital signs (body temperature, pulse rate and blood pressure), and weight
- CBC
- Biochemistry
- Blood sampling for ctDNA analysis
- Adverse events
- Re-assessment of Medical History / Signs and Symptoms
- Concomitant medications
- ECG
- Serum pregnancy test (for women of childbearing potential [see definition in Appendix II])
- Tumor sample by biopsy. A histological/cytological confirmation of recurrence is required

<u>Arm B:</u>

Eligible patients randomized to arm B will be treated **at the time of recurrence**.
Between baseline visit and treatment initiation (at recurrence), patients in arm B are followed on Day* 43 and then as per standard medical practice every 12-weeks with a physical examination of the head and neck including panendoscopy if appropriate, MRI or CT of the head and neck in the event of clinical signs or symptoms of recurrence during the first 24 months after randomization and every 24 weeks thereafter). Blood sampling for translational analysis is performed every 12 weeks during the first 24 months after randomization (two timepoints being 18 months and 24 months after randomization) and every 24 weeks thereafter until recurrence.
*Day corresponding to day post-randomization

The time windows allowed for physical examination of the head and neck **before recurrence** are:

- +/-1 week

The time windows allowed for blood sampling **before recurrence** are:

- +/- 4 weeks

<u>At recurrence</u>

- Physical examination of the major organ systems including vital signs (body temperature, pulse rate and blood pressure), and weight
- CBC
- Biochemistry
- Blood sampling for ctDNA analysis
- Medical History /Signs and Symptoms
- Concomitant medications
- ECG
- Serum pregnancy test (for women of childbearing potential [see definition in Appendix II])
- Tumor sample by biopsy. A histological/cytological confirmation of recurrence is required

<u>Arm A (if second series of TG4050 administrations) and Arm B:</u>

The time windows allowed for the study visits **during treatment** are:

- +/- 1 day from R-Day 1 to R-Day 43
- +/- 7 days beyond R-Day 43

<u>Following recurrence from R-Day 1 to R-Day 43</u>

On a weekly basis:

- Physical examination of major organ systems including vital signs (body temperature, pulse rate and blood pressure), and weight
- PS on ECOG scale
- Adverse events reporting
- Concomitant medications
- CBC (starting on R-Day 8)
- Biochemistry (starting on R-Day 8)

On an every three-weeks basis:

- Electrocardiogram (R-Days 22 and 43)
- Serum pregnancy test (R-Days 22 and 43 for women of childbearing potential [see definition in Appendix II])

For translational research:

- Blood samples on R-Days 1, 8, 15 and 43
- Skin biopsy on R-Days 8 and 43 at the site of previous injection if injection site reactions have been observed

Beyond R-Day 43

On an every three-weeks basis:

- Physical examination of major organ systems including vital signs (body temperature, pulse rate and blood pressure), and weight
- PS on ECOG scale
- Adverse events reporting
- Concomitant medications
- Serum pregnancy test (for women of childbearing potential [see definition in Appendix II])
- CBC & Biochemistry every 3 weeks up to Month 6 (R-Day 190), thereafter every 12 weeks

ECG every 6 weeks up to Month 6 (R-Day 190), thereafter every 12 weeks

For tumor assessment, CT or MRI of the head and neck and CT-scan of the chest will be performed every 6 weeks until documented disease progression according to RECIST 1.1.

For translational research:

- Blood samples starting from R-Day 64 every 12 weeks until progressive disease according to RECIST 1.1, and at progressive disease
- Apheresis on R-Day 64, if not clinically feasible, the apheresis procedure can be replaced by a 150 ml blood draw (not applicable for patients in Arm A who had an apheresis or a 150 ml blood sampling performed during the first series of TG4050 administrations)

6.2.4 End of treatment visit in the Phase I part

An end of treatment visit will be performed as soon as study treatment is definitely stopped for whatever reason.

The following evaluations will be conducted:

- Physical examination of major organ system, including vital signs (body temperature, pulse rate and blood pressure), and weight
- PS on ECOG scale
- Adverse events reporting
- Concomitant medications
- CBC
- Biochemistry
- Serum pregnancy test (for women of childbearing potential [see definition in Appendix II])
- Electrocardiogram
- Reporting of subsequent cancer therapy, if applicable

For translational research:

- Blood samples
- Tumor sample will be collected at the time of disease progression. A histological/cytological confirmation is required

6.2.5 Follow-up observation for both treatment arms (+/- 7 days) after completed End of Treatment Visit in the Phase I part

For patients who completed or prematurely stopped (due to other reason than progressive disease) the study treatment before documentation of disease progression, tumor assessment should be pursued every 6 weeks until documented disease progression according to RECIST 1.1 or until the date of last contact if the patient is lost to follow-up or withdraws consent. Tumor assessments will be recorded in the eCRF. All subsequent antineoplastic therapies (if applicable) must be recorded in the eCRF with start and end dates. A blood sampling for translational analysis will be performed according to section 6.2.3 (From R-Day 1 to R-Day 43 and Beyond R-Day 43) until progressive disease according to RECIST 1.1 or until the date of last contact if the patient is lost to follow-up or withdraws consent.
Patients in Arm A who definitely stopped TG4050 during the first series of administrations will perform as well an End of Treatment visit (please refer to section 6.2.4). They will have a physical examination of head and neck including panendoscopy if appropriate, MRI or CT of the head and neck in the event of clinical signs or symptoms of recurrence every 12 weeks during the first 24 months after randomization and every 24 weeks thereafter until recurrence documented clinically and by imaging or until the date of last contact if the patient is lost to follow-up or withdraws consent. A blood sampling for translational analysis will be performed according to section 6.2.3 (From Day 1 to Day 43 and Beyond Day 43) until recurrence documented clinically and by imaging or until the date of last contact if the patient is lost to follow-up or withdraws consent.

6.2.6 Long-Term Follow-Up after completion or stop of TG4050 treatment in the Phase I part

As per the guideline on follow-up of patients administered with gene therapy medicinal products (reference EMEA/CHMP/GTWP/60436/2007), long-term follow-up observations have been implemented in this trial for participants having received TG4050. In the phase I part this applies to patients in Arm A having received the first series of TG4050 administrations

before disease recurrence and for both arms having received TG4050 in combination with SOC after disease recurrence. The observations will be performed after the last TG4050 administration for a duration of five years and will consist of a clinical history (any relevant clinical events [*e.g.*, immune disease]), a physical examination and a blood sampling for immune response to the vaccine at 6 months and 12 months after end of treatment and then yearly up to 5 years after end of treatment. In case of a detection/suspicion of an AE related to TG4050, further investigations will be performed as appropriate, adapted to the nature of the adverse reaction observed and according to the state-of-the-art analyses available.

6.2.7 Study visits during the treatment period in the Phase II part

Experimental Arm:

Eligible patients randomized to the experimental arm will start treatment with TG4050 within 1 week after randomization.

From Day 1 to Day 43

On a weekly basis:

- Physical examination of the major organ systems, including vital signs (body temperature, pulse rate and blood pressure), and weight
- PS on ECOG scale
- Adverse events reporting
- Concomitant medications
- CBC (starting on D8)
- Biochemistry (starting on D8)

On an every three-weeks basis:

- Electrocardiogram (Days 22 and 43)
- Serum pregnancy test (Days 22 and 43 for women of childbearing potential [see definition in Appendix II])

On Day 43 physical examination of Head and Neck including panendoscopy if appropriate, MRI or CT of the head and neck in the event of clinical signs or symptoms of recurrence as per standard medical practice.

For translational research:

- Blood samples on days 15 and 43

Beyond D43

On an every three-weeks basis:

- Physical examination of the major organ systems including vital signs (body temperature, pulse rate and blood pressure), and weight
- PS on ECOG scale
- Adverse events reporting
- Concomitant medications

- Serum pregnancy test (for women of childbearing potential [see definition in Appendix II])
- CBC & Biochemistry every 3 weeks up to Month 6 (Day 190), thereafter every 12 weeks

ECG every 6 weeks up to Month 6 (Day 190), thereafter every 12 weeks

Physical examination of Head and Neck including panendoscopy if appropriate, MRI or CT of the head and neck in the event of clinical signs or symptoms of recurrence as per standard medical practice every 12 weeks during the first 24 months after randomization and every 24 weeks thereafter.

For translational research:

- Blood samples starting from Day 64 every 12 weeks during the first 24 months after randomization and every 24 weeks thereafter and at recurrence
- In selected centers: apheresis on day 64, if not clinically feasible, the apheresis procedure can be replaced by a 150 ml blood draw. In centers not selected for apheresis procedure, a 150 ml blood draw is mandatory on Day 64
- Tumor sample at the time of recurrence.
  A histological/cytological confirmation of recurrence is required

The time windows allowed for the study visits **during treatment** are:
- +/- 1 day from Day 1 to Day 43
- +/- 7 days beyond Day 43

Between completion of 20 TG4050 administrations and recurrence patients are followed as per standard medical practice every 12 weeks with a physical examination including panendoscopy if appropriate, MRI or CT of the head and neck in the event of clinical signs or symptoms of recurrence during the first 24 months after randomization and every 24 weeks thereafter. Blood sampling for translational research is performed until recurrence every 12 weeks during the first 24 months after randomization (two timepoints being 18 months and 24 months after start of TG4050 treatment) and every 24 weeks thereafter.

The time windows allowed for physical examination of the head and neck **after treatment completion** are:
- +/-1 week

The time windows allowed for blood sampling after **treatment completion** are:
- +/- 4 weeks

<u>At recurrence</u>
- Tumor sample by biopsy. A histological/cytological confirmation of recurrence is required
- Blood sampling for translational research

After recurrence of disease documented clinically and by imaging, study participation is completed. Patients will receive medical care in accordance with site standards and generally accepted medical practice and depending on the patient's individual medical needs.

<u>Control arm:</u>

Eligible patients randomized to the control arm will undergo watchful waiting until recurrence documented clinically and by imaging.
Between baseline visit and recurrence, patients will be followed on Day* 43 and then as per standard medical practice every 12-weeks with a physical examination of the head and neck including panendoscopy if appropriate, MRI or CT of the head and neck in the event of clinical signs or symptoms of recurrence during the first 24 months after randomization and every 24 weeks thereafter. Blood sampling for translational research is performed on Days* 15, 43 and starting from Day* 64, every 12 weeks during the first 24 months after randomization (two timepoints being 18 months and 24 months after randomization) and every 24 weeks thereafter until recurrence.
*Day(s) corresponding to days post-randomization

The time windows allowed for physical examination of the head and neck **before recurrence** are:
- +/-1 week

The time windows allowed for blood sampling **before recurrence** are:
- +/- 4 weeks

<u>At recurrence</u>
- CBC
- Biochemistry
- Medical History /Signs and Symptoms
- Concomitant medications
- ECG
- Serum pregnancy test (for women of childbearing potential [see definition in Appendix II])
- Tumor sample by biopsy. A histological/cytological confirmation of recurrence is required
- Blood sampling for translational research

The time windows allowed for the study visits **during treatment** are:
- +/- 1 day from R-Day 1 to R-Day 43
- +/- 7 days beyond R-Day 43

<u>Following recurrence from R-Day 1 to R-Day 43</u>

On a weekly basis:
- Physical examination of major organ systems including vital signs (body temperature, pulse rate and blood pressure), and weight
- PS on ECOG scale
- Adverse events reporting
- Concomitant medications
- CBC (starting on R-Day 8)
- Biochemistry (starting on R-Day 8)

On an every three-weeks basis:
- Electrocardiogram (R-Days 22 and 43)
- Serum pregnancy test (R-Days 22 and 43 for women of childbearing potential [see definition in Appendix II])

For translational research:
- Blood samples on R-Days 1, 8, 15 and 43

<u>Beyond R-Day 43</u>

On an every three-weeks basis:

- Physical examination of major organ systems including vital signs (body temperature, pulse rate and blood pressure), and weight
- PS on ECOG scale
- Adverse events reporting
- Concomitant medications
- Serum pregnancy test (for women of childbearing potential [see definition in Appendix II])
- CBC & Biochemistry every 3 weeks up to Month 6 (R-Day 190), thereafter every 12 weeks

ECG every 6 weeks up to Month 6 (R-Day 190), thereafter every 12 weeks

For tumor assessment, CT or MRI of the head and neck and CT-scan of the chest will be performed every 6 weeks.

For translational research:

- Blood samples starting from R-Day 64 every 12 weeks until progressive disease according to RECIST 1.1, and at progressive disease
- In selected centers, apheresis on R-Day 64, if not clinically feasible, the apheresis procedure can be replaced by a 150 ml blood draw. In centers not selected for apheresis procedure, a 150 ml blood draw is mandatory

6.2.8 End of treatment visit in the Phase II part

An end of treatment visit will be performed as soon as study treatment is definitely stopped for whatever reason. In the experimental arm this corresponds to the completion of a total of 20 TG4050 injections or to time of recurrence documented clinically and by imaging or to time of experiencing unacceptable toxicity including occurrence of a dose-limiting toxicity or to time of patient withdrawal for any reason whichever occurs first.
In the control arm this corresponds to the completion of a total of 20 TG4050 injections or to time of disease progression according to RECIST 1.1 or to time of experiencing unacceptable toxicity including occurrence of a dose-limiting toxicity or to time of patient withdrawal for any reason whichever occurs first.

The following evaluations will be conducted:

- Physical examination of major organ system, including vital signs (body temperature, pulse rate and blood pressure), and weight
- PS on ECOG scale
- Adverse events reporting
- Concomitant medications
- CBC
- Biochemistry
- Serum pregnancy test (for women of childbearing potential [see definition in Appendix II])
- Electrocardiogram
- Reporting of subsequent cancer therapy, if applicable

For translational research:
- Blood samples

6.2.9 Follow-up for both treatment arms (+/- 7 days) after completed End of Treatment Visit in the Phase II part

Experimental arm: Patients who completed or prematurely stopped the study treatment before documentation of recurrence clinically and by imaging, will have a physical examination of the head and neck including panendoscopy if appropriate, MRI or CT of the head and neck in the event of clinical signs or symptoms of recurrence on Day 43, and then every 12 weeks during the first 24 months after randomization and every 24 weeks thereafter until recurrence documented clinically and by imaging or until the date of last contact if the patient is lost to follow-up or withdraws consent. A blood sampling for translational analyses will be performed according to section 6.2.7 (From Day 1 to Day 43 and Beyond Day 43) until recurrence documented clinically and by imaging or until the date of last contact if the patient is lost to follow-up or withdraws consent. All subsequent antineoplastic therapies (if applicable) must be recorded in the eCRF with start and end dates.
Control arm: Patients who completed or prematurely stopped (due to other reason than progressive disease) the study treatment before documented disease progression according to RECIST 1.1 should be pursued every 6 weeks until documented disease progression according to RECIST 1.1 or until the date of last contact if the patient is lost to follow-up or withdraws consent. A blood sampling for translational analyses will be performed according to section 6.2.7 (From R-Day 1 to R-Day 43 and Beyond R-Day 43) until progressive disease according to RECIST 1.1 or until the date of last contact if the patient is lost to follow-up or withdraws consent. All subsequent antineoplastic therapies (if applicable) must be recorded in the eCRF with start and end dates.

6.2.10 Long-Term Follow-Up after completion or stop of TG4050 treatment in the Phase II part

As per the guideline on follow-up of patients administered with gene therapy medicinal products (reference EMEA/CHMP/GTWP/60436/2007), long-term follow-up observations have been implemented in this trial for participants having received TG4050. In the phase II part this applies to patients in the experimental arm having received TG4050 before disease recurrence and to patients in the control arm having received TG4050 in combination with SOC after disease recurrence. The observations will be performed after the last TG4050 administration for a duration of five years and will consist of a clinical history (any relevant clinical events [e.g., immune disease]), a physical examination and a blood sampling for immune response to the vaccine at 6 months and 12 months after end of treatment and then yearly up to 5 years after end of treatment. In case of a detection/suspicion of an AE related to TG4050, further investigations will be performed as appropriate, adapted to the nature of the adverse reaction observed and according to the state-of-the-art analyses available.

6.2.11 Safety follow-up visit (+ 7 days)

A safety follow-up visit will be performed at least 30 days after having definitely stopped any study treatment.
The following evaluations will be conducted:

- Physical examination of major organ system, including vital signs (body temperature, pulse rate and blood pressure), and weight
- PS on ECOG scale
- Adverse events reporting
- Concomitant medication
- Serum pregnancy test (for women of childbearing potential [see definition in Appendix II])
- Reporting of subsequent cancer therapy if applicable

6.2.12 Medical care of patients after end of study

After a patient has completed the trial or has withdrawn early, usual treatment will be administered, if required, in accordance with the trial site's standard of care and generally accepted medical practice and depending on the patient's individual medical needs.

7. TREATMENT PLAN FOR TREATMENT PERIOD

7.1 Investigational Medicinal Product (IMP) characteristics

In this clinical trial, the Investigational Medicinal Product (IMP) is TG4050.

TG4050 is a suspension of a recombinant MVA strain, a significantly attenuated strain of Vaccinia virus, containing DNA sequences coding for several tumor specific neoepitopes identified for each patient.

PPD [redacted]

TG4050 will be supplied along with a Technical Sheet detailing its characteristics.

7.1.1 Manufacturing

For patients who meet screening period eligibility criteria, tumor tissue from primary tumor which have been collected at the time of surgery and blood samples will be tested for TSAs identification. TSAs will be used to manufacture an individualized TG4050 mutanome-directed immunotherapy. Manufacturing of the vaccine in accordance with GMP procedures will be done during the patient undergoes adjuvant therapy.

In case of TG4050 individualized construct unavailability in due time or no injection of TG4050 manufactured product for any reason, the reason will be documented as a Failure to provide or a Failure to treat and the patient will be withdrawn from the study. Patients will continue to benefit from routine medical monitoring and will receive SOC in case of recurrence as per normal practice.
In case of Failure to provide, another round of manufacturing attempt will not be considered.

7.1.2 Packaging, labeling and shipping

TG4050 is supplied as a frozen suspension filled in individual 4-mL glass vials (Type I glass) (primary packaging) and packed in cardboard boxes (secondary packaging). Each vial is

intended for single use (i.e., 1 injection to 1 patient). The recoverable volume of TG4050 in each vial is 0.5 mL with an infectious titer of $1x10^8$ PFU ($2x10^8$ PFU/mL). TG4050 is a colorless to whitish clear or slightly turbid liquid, with possible presence of product particles or filaments.

The primary label on the vial as well as the secondary label (on the secondary packaging) are in the language(s) of countries where the trial is conducted. Labels are compliant with local regulatory requirements.

Packaging for shipment of TG4050 is compliant with the IATA (International Air Transport Association) and ADR (International Carriage of Dangerous Goods by Road) regulations for air and road transport of infectious substances (UN 373 regulations). TG4050 is shipped in proper transport conditions with the official transport designation "Biological Substance, Category B" by a carrier specialized in Genetically Modified Organisms (GMO) transportation.

7.1.3 Conditions of storage and use

TG4050 must be stored at -20°C **+/-** 5°C (between -15°C and -25°C) in its original package, in an alarmed and monitored freezer in a secure place with restricted access until use under the supervision of the study Pharmacist / Investigator (or his/her delegate). The vials are dispensed only with the written authorization of the Investigator to staff that are specifically designated and trained for this study.
Any storage temperature excursion above -15°C is considered out of specifications and must be documented and reported upon discovery to Transgene at:

PPD [redacted]

Once a temperature excursion is noted, the IMP must be quarantined and not used until Transgene provides documentation with the final decision: authorization granted or not to use the affected IMP units.
It will not be considered a protocol deviation if Transgene approves the use of the IMP after the temperature excursion. Use of the IMP prior to Transgene approval will be considered a protocol deviation.

TG4050 is a Genetically Modified Organism (GMO). As such, and whenever relevant, it must be handled according to national regulatory requirements. The Investigator's Brochure, Technical Sheet and Product Handling Manual provided by Transgene detail instructions for TG4050 handling (biosafety requirements, instructions for storage, preparation, administration, cleaning, decontamination, disposal and return) and incident management.

During all TG4050 handlings, labcoat, goggles, gloves and mask must be worn. All transport of TG4050 (vial or syringe containing the dose to be injected) must be done using a leakproof container/bag.

7.1.4 Preparation and administration procedure

TG4050 will be administered at the dose of 1x 10^8 PFU. TG4050 doses must be prepared and handled according to the instructions provided in a Preparation Procedure provided by Transgene.

TG4050 must be prepared under aseptic conditions in compliance with requirements for every human injectable preparation. Aseptic conditions mean the standard hospital conditions for injectable preparations that ensure the sterility of the TG4050 solutions. Once prepared and if not immediately administered, TG4050 product must be kept at room temperature for a maximum of 2 hours following vial removal from freezer up to its administration. It must not be refrozen.
If administration is delayed for over 2 hours, a new dose must be prepared and the dose which was not injected within 2 hours must be disposed according to the waste management instructions. All transfers of the TG4050 must be done using a leakproof container/bag.

Each TG4050 dose will be administered by a single SC injection alternating between scapular areas and thighs to identify potential injection site reactions after each administration.
Four injection sites will be used (left and right scapular area, left and right thigh) according to a rotation schedule. It is preferable that the first injection is administered in either the right or left thigh. The second injection is to be given in the opposite side scapular area, the third in the same side thigh, and the fourth in the opposite side scapular area. For example, if the left thigh is the site of the first vaccination, the second vaccination would be in the right scapular area, the third in the right thigh, and the fourth in the left scapular area. This pattern is recommended and will be repeated for subsequent injections.

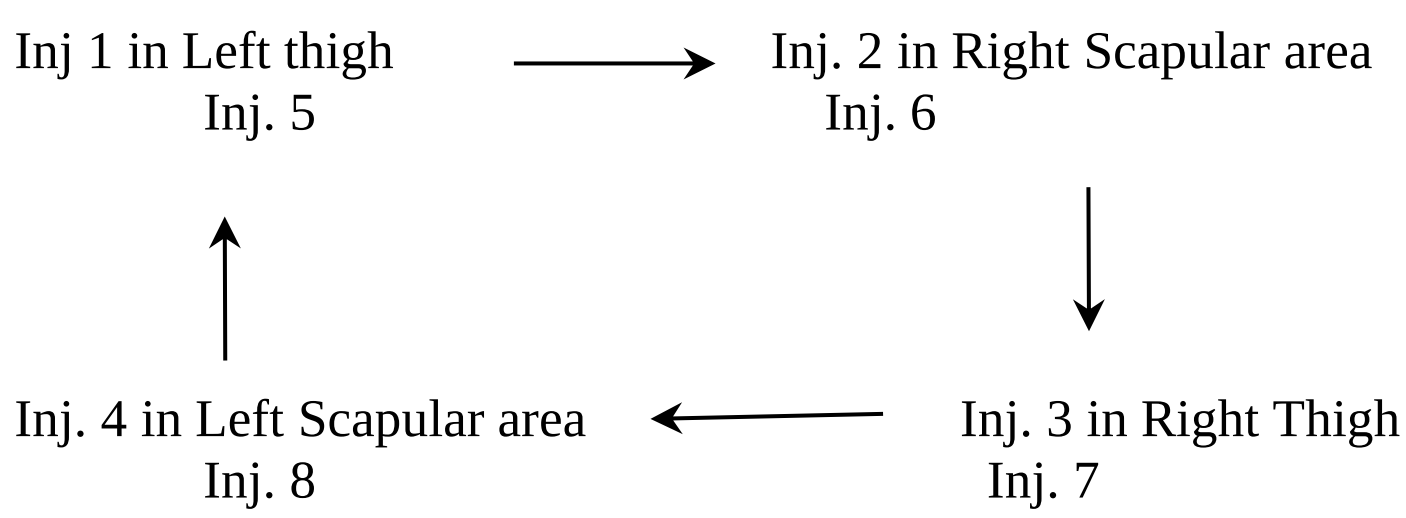

If the pattern cannot be followed exactly, it is more important to alternate between the scapular areas and thighs than side to side.

In case of incident while handling TG4050, the actions recommended are described in the Technical Sheet.

Any incident while handling TG4050 must be documented by a written report to be sent as soon as knowledge of the event to:

Transgene
Medical Affairs:

PPD [redacted]

Fax : (+33) (0)3 88 27 91 41

Emergency 24/7 telephone number (worldwide): **(+33) (0)3 68 33 28 80**

7.1.5 Disposal and destruction or return

During the course of the study based on Transgene's request and at termination of the study all unused TG4050 vials will be destroyed locally or returned to Transgene or its subcontractor.
For local destruction, the Investigator/Pharmacist or delegated person will ensure that destruction is performed according to written instructions for disposal of TG4050 and waste generated during the TG4050 preparation and administration (e.g. injected and non-injected syringes, needles, gloves, etc.) available in the TG4050 Investigator's Brochure and the Technical Sheet and will not expose humans to any risks from TG4050. A certificate of destruction will be completed and provided to Transgene (copy retained by the site).

Alternatively, unused vials of TG4050 may be returned to Transgene or its subcontractor with the appropriate documentation. Transgene will coordinate the return of unused vials of TG4050. A certificate of return will be completed and provided to Transgene (copy retained by the site).

All along the study, the monitor will verify that all used TG4050 vials and waste linked to TG4050 handling are disposed locally, all unused/expired vials are returned or destroyed locally, and no product remains on site upon completion or termination of the study.

Accurate records of TG4050 received at, dispensed from, returned to and disposal of by the study site should be recorded appropriately on the drug log.

7.1.6 Supplier

TG4050 is supplied by or on behalf of Transgene for this study at no cost to the study participant.

7.2 IMP treatment duration

Phase I part:
<u>In Arm A</u> patients will start treatment with TG4050 at completion of primary treatment as a single agent for up to a total of 20 injections or until recurrence documented clinically and by imaging or until unacceptable toxicity including dose-limiting toxicity or patient withdrawal for any reason, whichever occurs first. At recurrence patients will then be offered TG4050 in combination with standard of care (SOC) for up to a total of 20 injections, or until documented disease progression according to RECIST 1.1, or until unacceptable toxicity including dose-limiting toxicity or patient withdrawal for any reason whichever occurs first. If unacceptable toxicity was leading to treatment discontinuation before recurrence patient will be treated with SOC only after recurrence.
<u>In Arm B</u> patients will receive TG4050 in combination with SOC at recurrence documented clinically and by imaging for up to a total of 20 injections, or until documented disease progression according to RECIST 1.1, or until unacceptable toxicity including dose-limiting toxicity or patient withdrawal for any reason whichever occurs first.

For both arms: at the 1st radiologic evidence of PD, clinically stable patients may continue TG4050 treatment at the Investigator's discretion while awaiting a confirmatory scan and / or initiation of a further antineoplastic treatment.
Phase II part:
In the experimental arm patients will start treatment with TG4050 at completion of primary treatment as a single agent for up to a total of 20 injections or until recurrence documented clinically and by imaging or until unacceptable toxicity including occurrence of a dose-limiting toxicity or patient withdrawal for any reason, whichever occurs first.
In the control arm patients will undergo watchful waiting until recurrence documented clinically and by imaging. At recurrence, patients will start TG4050 in combination with SOC for up to a total of 20 injections or until documented disease progression according to RECIST 1.1 or until unacceptable toxicity including occurrence of a dose-limiting toxicity or patient withdrawal for any reason whichever occurs first.

7.3 IMP treatment modification

No dose reduction is allowed.
If a grade 1 AE occurs, TG4050 should be administered as planned. In case a grade ≥ 2 treatment-emergent AE occurs, that is at least possibly related to TG4050, the administration of TG4050 will be delayed until resolution to grade ≤1 with the following exceptions: fatigue and injection site reactions which are common vaccine-associated AEs. The decision to restart TG4050 or to permanently discontinue study treatment will be discussed between the investigator and the sponsor.

7.4 IMP treatment compliance

7.4.1 Dispensing and accountability

TG4050 is dispensed only with the written authorization of the Investigator to staff that are specifically designated and trained for this trial.

The Investigator / Pharmacist or delegated person will maintain IMP accountability records (paper or electronic format), detailing the dates and quantities dispensed for each patient along with IMP packaging batch numbers, box and vial numbers.

IMP accountability records will be verified by the monitor during site monitoring visits. All used and unused IMP will be accounted for. All unused/expired IMP will be destroyed locally or returned to Transgene or its subcontractor, providing destruction or return certificates.
Detailed information on the IMP destruction and return is provided in the Product Handling Manual (TG4050).

7.4.2 Assessment of compliance

The compliance for the IMP will be monitored through the IMP accountability log and information reported on the eCRF pages.

7.5 Standard of Care (SOC)

From disease recurrence documented clinically and by imaging procedures, all patients (in the phase I part) and patients in the control arm (in the phase II part) will receive Standard of care (SOC) treatment as part of study treatment. SOC can be surgery, chemotherapy or immunotherapy at the investigator's discretion according to the applicable SmPC or product label (when applicable) during study treatment period. All therapeutic adaptations and their reasons must be documented in the eCRF.
In case SOC is delayed, TG4050 administration should continue according to protocol.
For patients in the experimental arm (in the phase II part) study participation is completed at disease recurrence. From recurrence documented clinically and by imaging procedures, they will receive medical care in accordance with site standards and generally accepted medical practice and depending on the patient's individual medical needs.

7.6 Concomitant medications

In the four-week period prior to TG4050 treatment initiation any vaccination for the prevention of infectious diseases with a live vaccine or any COVID-19-vaccine is not permitted.

7.6.1 Prohibited and/or restricted medications during study treatment

The following medications are prohibited (unless utilized to treat a drug related adverse event):

- Immunosuppressive agents
- Immunosuppressive doses of systemic corticosteroids (except as stated in Section 7.6.3)
- Any live vaccine for the prevention of infectious diseases
- Any other investigational treatment

The following non-drug therapies must not be used during the study (and within 28 days before the start of trial treatment):

- Herbal remedies with immunostimulating properties (e.g., mistletoe extract) or known to potentially interfere with major organ function (e.g., hypericin)
- Major surgery

If the use of a non-permitted concomitant therapy becomes necessary during the trial, the patient should be withdrawn from trial treatment (the Sponsor may be contacted to discuss whether the trial treatment must be discontinued).

7.6.2 Other restrictions and precautions

Patients with a condition requiring systemic treatment with either corticosteroids (> 10 mg daily prednisone equivalent) or other immunosuppressive medications within 14 days prior TG4050 treatment initiation are excluded.

7.6.3 Permitted medications/therapies

- Patients are permitted the use of topical, ocular, intra-articular, intranasal, and inhalational corticosteroids (with minimal systemic absorption).

- The use of the following drugs will not be restricted during the course of the study: G-CSF, erythropoietin, antiemetics and analgesics.
- The use of systemic corticosteroids during the study is restricted as follows: for short term treatment (≤7 days) of allergic reactions, or for replacement for adrenal insufficiency at doses equivalent to ≤ 10 mg prednisone daily.
- Any medications (other than those excluded by the study protocol) that are considered necessary for the patients' welfare and will not interfere with the trial medication may be given at investigator's discretion.

8. EVALUATION

8.1 Evaluation during the screening period

The percentage of patients who cannot be enrolled in the treatment period and the reason that prevents patients from being treated will be evaluated.
The reason can be:

- Failure to provide the vaccine
- Lost to follow-up
- Non-eligibility of the patients for the treatment period.

8.2 Evaluation during the treatment period

8.2.1 Efficacy evaluation

<u>Before recurrence</u>
At Baseline and within 3 weeks prior to randomization imaging procedures to confirm complete locoregional response and absence of distant metastases.
During treatment period and before recurrence a comprehensive head and neck examination will be performed (please refer to section 6.2) In the event of clinical signs or symptoms.: CT or MRI of the head and neck and CT-scan of the chest are mandatory.

<u>After recurrence (both arms in the phase I part and control arm in the phase II part)</u>
Tumor assessment will be performed within 21 days (± 7 days) before treatment start after disease recurrence and then every 6 weeks until documented disease progression and will require CT or MRI of the head and neck and CT-scan of the chest. Tumor response will be evaluated according to RECIST 1.1. Throughout the study, the same assessment technique must be used.

8.2.2 Safety evaluation

Safety assessments will consist of monitoring and reporting of all adverse events (AEs) and serious adverse events (SAEs), physical examination findings including vital signs and laboratory tests (see APPENDIX III).
The safety assessments will be performed according to the schedule of assessments in APPENDIX III.

8.2.2.1 Definitions

Treatment-Emergent Adverse Event (TEAE)
A treatment-emergent adverse event (TEAE) is defined as a sign or symptom that emerges during treatment or within 30 days of the last dose of the study drug having been absent pre-treatment or that has worsened relative to the pre-treatment state. In the phase I part this corresponds to completion of first series of 20 TG4050 administrations in Arm A and to safety follow-up visit in both arms. In the phase II part this corresponds to safety follow-up visit in both arms.

An Adverse Event (AE***)*** is defined as any new untoward medical occurrence or worsening of a preexisting medical condition in a clinical investigation patient administered study treatment and that does not necessarily have a causal relationship with this treatment. An AE can therefore be any unfavorable and unintended sign (such as an abnormal laboratory finding), symptom, or disease temporally associated with the use of study treatment, whether or not considered related to the study treatment.

Adverse reaction (AR)
All noxious and unintended responses to a medicinal product related to any dose or to a study specific procedure.

Dose-limiting toxicity (DLT)
In the phase I part, a safety run-in phase will be conducted for the first 3 treated patients in each arm who will be observed during 6 weeks after the first administration of TG4050. During this phase and per arm, each patient will be monitored for 2 weeks after the first injection of TG4050 (and having received 2 injections of TG4050) before the next patient can be enrolled in the same arm.
Enrollment of the next patients will be allowed if none of the patients has experienced during the six weeks run-in phase a dose-limiting toxicity defined as:

- any treatment-emergent AE grade ≥3 toxicity that is at least possibly related to TG4050, with the following exception: pre-existing grade 2 fatigue at baseline that would worsen up to grade 3
- any death.

The occurrence of any of those events in the initial 3 treated patients of each arm will trigger a study hold within the safety run-in phase.

Beyond the safety run-in phase, a dose-limiting toxicity is defined, in the phase I part and in the phase II part, as any treatment-emergent AE grade ≥3 toxicity that is at least possibly related to TG4050, with the following exception: pre-existing grade 2 fatigue at baseline that would worsen up to grade 3.

In any case, patients experiencing a dose-limiting toxicity will discontinue TG4050 treatment permanently.

Serious adverse events
A ***Serious Adverse Event (SAE)*** is any untoward medical occurrence that at any dose:

- results in death

- is life-threatening (defined as an event in which the patient was at risk of death at the time of the event; it does not refer to an event which hypothetically might have caused death if it were more severe)
- requires inpatient hospitalization or causes prolongation of existing hospitalization (see NOTE below)
- results in persistent or significant disability/incapacity
- is a congenital anomaly/birth defect
- is an important medical event (defined as a medical event(s) that may not be immediately life-threatening or result in death or hospitalization but, based upon appropriate medical and scientific judgment, may jeopardize the patient or may require intervention [e.g., medical, surgical] to prevent one of the other serious outcomes listed in the definition above). Examples of such events include, but are not limited to, intensive treatment in an emergency room or at home for allergic bronchospasm; blood dyscrasias or convulsions that do not result in hospitalization. Potential drug induced liver injury (DILI) is also considered an important medical event.

Suspected transmission of an infectious agent (e.g., pathogenic or nonpathogenic) via the study drug is an SAE.
Although pregnancy and overdose are not always serious by regulatory definition, these events must be handled in the same way as SAEs (for specific forms for pregnancy and overdose see section 8.2.4).

<u>NOTE</u>
The following situations do not need to be reported as SAEs:

- Hospitalization for monitoring or elective treatment of pre-existing disease(s) or condition(s) present at baseline, unrelated or related to the indication under trial and that do not worsen
- Hospitalization for monitoring or routine treatment of the studied indication, not associated with any deterioration in condition
- Optional admission not associated with a precipitating clinical AE (e.g., for elective cosmetic surgery)
- Hospitalization for explorations or same-day surgeries (as outpatient/same day/ambulatory procedures)
- Hospitalization for treatments or surgical procedures which was planned before the patient consented for study participation and where admission did not take longer than anticipated.
- Hospitalization planned for protocol related treatment or protocol related procedure as per institutional standard timelines
- Medical or surgical procedure (e.g., appendectomy); the condition that leads to the procedure is an (S)AE
- Situations where an untoward medical occurrence did not occur (palliative care, rehabilitation, overdose without occurrence of an adverse event)
- Social and/or convenience admission to a hospital
- Administrative admission (e.g., for yearly physical examination)
- Admission exclusively for the administration of blood products

Suspected Unexpected Serious Adverse Reaction (SUSAR):

A SUSAR is any unexpected SAE considered as related to the study treatment (auxiliary MP or IMP) or to a study specific procedure.
Expected adverse events are those adverse reactions that are listed or characterized in the reference document (e.g., Package Insert or IB).
Unexpected adverse reactions are those not listed in the reference document. This includes adverse events for which the nature, specificity, severity or outcome are not consistent with the description in the reference document.

8.2.2.2 Time period for collection

From the date of signature of ICF and up to first TG4050 administration, only serious adverse reactions (SARs) caused by a protocol-required procedure will be collected and reported (refer to section 8.2.5. and 8.2.8). The only protocol-required procedure concerned is the specific blood sample taken during the screening period.

AEs, SAEs, or procedure related ARs that occur during this period from the date of signature and up to 21 days before first TG4050 administration and that are considered by the investigator as particularly relevant to the patient's condition, will be only collected as a current medical history.

If an event occurs during the period from 21 days before and up to first TG4050 administration, it will be recorded either on a "Medical history/current conditions" page for an identified syndrome (e.g., pneumonia) or on a "Signs and symptoms" page for symptoms with no associated syndrome (e.g., diarrhea).

From the first TG4050 administration and in case of worsening of a medical history/sign and symptom which has been present within the 21 days prior to first TG4050 administration an AE page will be completed using a verbatim starting by "worsening of..."

From the initiation of TG4050 treatment up to 30 days after the last administration of study treatment all AEs and SAEs should be collected and recorded on the eCRF. In the phase I part this corresponds to completion of first series of 20 TG4050 administrations in Arm A and to safety follow-up visit in both arms. In the phase II part this corresponds to safety follow-up visit in both arms.
During this period all SAEs should also be reported to Transgene.
All SARs related to the study specific procedure occurring during the screening period and all SAEs occurring during the treatment period will be recorded in the Sponsor's safety data base.

SAEs evaluated by the investigator as at least possibly related to TG4050 occurring more than 30 days after administration of the last dose of TG4050 must be collected and reported to the sponsor indefinitely even after study closure. These will however not be reported in the eCRF after Database lock. All (S)ARs related to protocol-required procedures must be collected and reported until completion of End of Study form.

8.2.2.3 Adverse event / Serious adverse event management

8.2.2.3.1 Data collection

Reporting in eCRF

At each visit, all AEs (as defined in 8.2.2.2) whether volunteered by the patient, discovered by study personnel during questioning, or detected through physical examination, laboratory test, or other means will be reported appropriately on the page "Adverse Events" of the eCRF (= AE page). The following items must be documented:

- nature of the event with self-explanatory and concise medical terminology (if possible indicate a diagnosis or syndrome instead of symptoms),
- date of onset and date of end (i.e. actual dates when the event starts and is resolved rather than dates when the Investigator is informed),
- intensity,
- evaluation of seriousness,
- relation to study treatment or to study procedures,
- action taken regarding the study treatment,
- action taken regarding the event,
- outcome.

AE / AR requiring therapy must be treated with recognized standards of medical care to protect the health and well-being of the patient. Any treatment given will be reported on the page "Concomitant medication" of the eCRF.
Appropriate resuscitation equipment and medicines must be available to ensure the best possible treatment of an emergency situation.

Intensity

The intensity of AE/SAE will be graded according to the NCI-CTCAE (version 5) which will be provided to the Investigators.

Should an event be missing in the CTCAE, the following 5-point scale is to be used:

- Mild: Discomfort noticed, but no disruption of normal daily activity
- Moderate: Discomfort sufficient to affect normal daily activity
- Severe: Inability to work or perform normal daily activity
- Life-threatening: Risk of death at the time of the event
- Fatal: The patient died

The correspondence between the two scales is as follows:

CTCAE	5-point scale
1	Mild
2	Moderate
3	Severe
4	Life-threatening
5	Fatal

Any increase in severity category during the course of an adverse event should be reported on the AE pages of the eCRF with corresponding dates.

Causality

The relationship to IMP and/or chemotherapy, and/or immunotherapy and/or surgery if received by the patient during treatment period after recurrence (separate evaluation for TG4050, chemotherapy, immunotherapy and surgery, if applicable) of each AE/SAE will be evaluated by the Investigator with" EudraVigilance Clinical Trial Module" (EVCTM) method using the following levels:

- **No reasonable possibility** i.e. no facts (evidence) or arguments to suggest a causal relationship: the temporal relationship of the clinical event to the administration of study treatments makes a causal relationship unlikely; and other drugs, therapeutic interventions or underlying conditions provide a sufficient explanation for the observed event.
- **Reasonable possibility** i.e. facts (evidence) or arguments to suggest a causal relationship: the temporal relationship of the clinical event to the administration of the study treatments makes a causal relationship possible; and other drugs, therapeutic interventions or underlying conditions do not provide a sufficient explanation for the observed event. A clear-cut temporal association with improvement on cessation of the study treatment or recurrence upon rechallenge may also be observed.

Outcome

The outcome is rated as follows:

- recovered
- not recovered
- recovered with sequelae
- fatal
- unknown
- worsening
- recovering

Note on "fatal": this outcome is to be used only for the event leading to death. The outcome of all other events at the time of the death must be reported. The outcome of ongoing ones is reported as "not recovered".

Action taken regarding the IMP

- None: The administration continues as planned in the protocol
- Delayed: The administration following the occurrence of the event is delayed
- Cancelled: The administration following the occurrence of the event is cancelled
- Stopped: The administration is definitively stopped
- Not applicable: The administration is already stopped, or not yet started

Action taken regarding the SOC

- None: The administration continues as planned in the protocol

- Delayed: The administration following the occurrence of the event is delayed
- Cancelled: The administration following the occurrence of the event is cancelled
- Stopped: The administration is definitively stopped
- Dose changed: For the next administration dose is changed
- Not applicable: The administration is already stopped, or not yet started

Action taken regarding the event:

- New treatment prescribed or change in concomitant medication
- Hospitalization or prolongation of hospitalization
- Non-drug therapy implemented
- None
- Other (to be specified)

8.2.2.3.2 Follow-up

Once an AE is detected, it must be proactively followed until its resolution or 30 days after the last study treatment administration (for definitions, please refer to section 8.2.2.2 Time period for collection).

However, the AEs listed below must be followed until they are resolved or stable or returned to baseline status, which may occur after the safety follow-up visit planned by the protocol:

- AE evaluated as related to the IMP
- SAEs
- Any other significant AE as recommended by Transgene

Transgene or its representative reserves the right to ask for further information on any AE/SAE that may be considered of interest or when the event is not previously documented in the IB (new occurrence) or Package Insert and is thought to be related to the IMP.

8.2.2.3.3 Documentation

All AEs/SAEs will be reported in the source document with at least the nature, the date of onset and end, the causality, and the treatment (if applicable).

Copies of SAE form will be filled in the Investigator Site File along with copies of any correspondence with the Independent Ethics Committee (IEC) / Institutional Review Board (IRB). The Investigator Site File will also include copies of notification letters and/or faxes with forms sent to Health Authorities and Gene Therapy Bodies if appropriate.

8.2.2.3.4 Notification to investigators

In case of grade ≥ 3 AE suspected to be at least possibly related to TG4050 occurs and as soon as they became aware of, the Sponsor or its representative will inform all investigators involved in the study that such an event has been reported.

8.2.3 Serious Adverse Event / Serious Adverse Reaction / Suspected Unexpected Serious Adverse Reaction notification

Reporting to Transgene

Any SAE occurring during the screening period (after ICF signature and before TG4050 treatment initiation) related to a study specific procedure (specific blood sample taken during this screening period) MUST be reported to Transgene.

Any SAE occurring from TG4050 treatment initiation and up to 30 days after the last administration of study treatment whether or not related to TG4050 MUST be reported to Transgene. In the phase I part this corresponds to completion of first series of 20 TG4050 administrations in Arm A and to safety follow-up visit in both arms. In the phase II part this corresponds to safety follow-up visit in both arms.

SAEs evaluated by the investigator as at least possibly related to TG4050 occurring more than 30 days after administration of the last dose of TG4050 MUST be reported to Transgene. All (S)ARs related to protocol-required procedures must be collected and reported until completion of End of Study form.

The Investigator must complete and send (preferably by email otherwise by fax) a "Serious Adverse Event Form" to Transgene within 24 HOURS of occurrence or knowledge of the event.

	**France**
	**Pharmacovigilance Physician**
**Name**	PPD [redacted]
**Phone number**	PPD [redacted]
**Fax number**	PPD [redacted]
**Email**	PPD [redacted]
**Emergency 24-hour telephone number**	PPD [redacted]

An Investigator's designee may complete the SAE form; however, the Investigator must sign it. The form can be sent to Transgene with the designee's signature if the Investigator's signature cannot be obtained within one working day. The Investigator's signature must be obtained as soon as possible, as well as his/her evaluation of the relationship to the study treatment. The signed form must be sent (preferably by email otherwise by fax) to Transgene immediately.

The SAE form must be completed in English.

Follow-up information (e.g., complications or progression of the initial SAE) must be notified to Transgene by the Investigator within the same time frame as the occurrence by using a new "SAE form" with the box "follow-up" ticked and sent to the Transgene.

The follow-up information should describe whether the event has resolved or continues, if and how it was treated and whether the patient continued or withdrew his/her consent.

Transgene or its representative may request further information as needed.

All SAEs will be followed to resolution or stabilization.

Notification to Regulatory Authorities / Gene Therapy Bodies / Ethics Committee

The Sponsor or its representative is responsible for ensuring that all AEs and SAEs that are observed or reported during the study are collected and reported to the FDA in accordance with CFR 312.32 (IND Safety Reports).

The Sponsor or its representative will be responsible for reporting of SUSAR and any other SAEs to Regulatory Authorities, IEC(s) / IRB(s) and Gene Therapy Bodies as per local regulation. In case of a SUSAR, the Sponsor or its representative will inform all investigators involved in the study that such an event has been reported.
The Investigator is responsible for informing local Ethics Committee of SUSARs, any other SAEs, and any follow-up information as per local regulations.

8.2.4 Special situations

Serious Adverse Reactions related to auxiliary MPs (chemotherapy, immunotherapy or surgery)

The Sponsor will report as SUSAR unexpected serious adverse reactions related to chemotherapy and/or immunotherapy received in combination with TG4050 by the patient during treatment period after recurrence (both arms in the phase I part and control arm in the phase II part).

Pregnancy
Study treatment must be discontinued immediately when a patient becomes pregnant.
The occurrence of a pregnancy must be handled in the same way as a SAE, i.e. reported to Transgene by the Investigator within 24 HOURS using a “Pregnancy form”:

- The first part of the form is used to collect information at the beginning of the pregnancy. It should be completed and reported as soon as possible and within 24 hours of knowledge
- The second part of the form is to collect information about the outcome of the pregnancy. It should be reported within the same time frame and even if the patient was withdrawn from the study

Pregnancies have to be followed up to the completion or termination of the pregnancy, collecting information about the pregnancy and knowledge of the new-born medical status. Pregnancy outcomes must be collected for the female partners of any males who took the IMP. Consent to report information regarding these pregnancy outcomes should be obtained from the mother.
Pregnancy itself is not considered as an AE. However, any problem met during the pregnancy should be reported as an AE or a SAE. Spontaneous or induced abortions as well as ectopic pregnancy should be considered as serious. Similarly, any congenital anomaly/birth defect in a child born to a female patient exposed to the IMP (or to the female partner of a male exposed patient) should be reported as an SAE.
The time period for collecting new pregnancies is from the first administration of the IMP up to 3 months after the last study treatment administration.

Overdose
An overdose is defined as the accidental or intentional administration of any dose of a product that is considered both excessive and medically important.
For this study, any dose of IMP greater than the assigned dose will be considered an overdose.

All occurrences of overdose must be reported in the same way as SAEs, i.e. reported to Transgene by the Investigator within 24 HOURS using an "Overdose form" (see Section 8.2.3). In the event of an overdose, the Investigator or treating physician should:

- Contact the Medical Monitor immediately
- Closely monitor the participant for AEs/SAEs and laboratory abnormalities
- Document the quantity of the excess dose as well as the duration of the overdosing in the eCRF

For all reports of overdose (whether accidental or intentional) with an associated AE, the AE term should reflect the clinical symptoms or abnormal test result. Additionally, the verbatim term should state "due to overdose".

An overdose without any associated clinical symptoms or abnormal laboratory results should be reported on an AE page in the eCRF using the terminology "accidental or intentional overdose without adverse effect."

Decisions regarding dose interruptions or modifications secondary to an overdose will be made by the Investigator in consultation with the Medical Monitor based on the clinical evaluation of the participant.

Other safety considerations

Any significant worsening noted during interim or final physical examinations, electrocardiogram, CT-scan, any other potential safety assessment required or not required by protocol should also be recorded as a non-serious or serious AE, as appropriate, and reported accordingly.

8.2.5 Laboratory values, vital signs, physical findings and other safety data

Laboratory tests results will be recorded on the laboratory results forms of the eCRF.

Clinically relevant abnormal laboratory or tests results, clinically relevant abnormal findings in vital signs measurements, physical examinations or ECGs will be reported by the Investigator and followed until normal, stabilization or back to baseline value or until the safety follow-up visit if they are not related to the IMP. If related to the IMP, they should be followed even after the safety follow-up visit (see Section 8.2.2.3.2).

Any laboratory result abnormality fulfilling the criteria for a serious adverse event (SAE) should be reported as such, in addition to being recorded as an AE on the eCRF.

Any treatment-emergent abnormal laboratory result which is clinically significant, i.e., meeting one or more of the following conditions, should be recorded as a single diagnosis on the adverse event form in the eCRF:

- accompanied by clinical symptoms
- leading to a change in study medications (e.g., interruption or permanent discontinuation)
- requiring corrective treatment or clinical management

8.2.6 Translational research and exploratory evaluations

Mutational load and impact of vaccination on tumor mutational profile; specifically, tumor exome and transcriptome sequencing prior to initiation of treatment will allow identification of

tumor specific mutations and their respective level of expression in tumor tissue. Similarly, tumor immune infiltrates will be characterized by immunohistochemistry, specifically, density of infiltrates of CD8, and levels of expression of activation markers (Ki-67 [only in the phase I part], HLA-DR) or immune related markers (PD-L1, lag3, and Tim3, HLA) will be performed as well as staining for regulatory T cells (CD4/CD25/FoxP3) and MDSC as these factors are known to be a prognostic value in patients receiving immunotherapies.

Frequency and level of activation of immune cell subpopulations in tumor and peripheral blood: viral vaccination is expected to stimulate the immune system and leads to change in circulating immune cells as well as in tumor immune infiltrate. Specifically, vaccination may lead to increased frequencies and levels of activation of cells related to innate and adaptive response, and on the contrary, in decreased frequencies of immunosuppressive cells. The phenotype of circulating immune cells will be performed by flow cytometry (or equivalent methods) and will include the characterization of CD8+, CD4+ compartments as well as regulatory T cells, NK cells, dendritic cells, plasmacytoid dendritic cells, neutrophils and myeloid-derived suppressor cells (MDSC).

Intensity of antigen specific response; vaccines are expected to elicit an immune mediated tumor rejection by priming the immune system and leading to the development of tumor antigen specific effector T cells. These T cells will have the capacity to specifically recognize tumor specific epitopes. To validate this mechanism, peripheral blood mononuclear cells will be isolated and tumor antigen specific cells frequency will be evaluated by ELISPOT, multimer staining.

In selected patients, breadth of immune response will be assessed by sequencing of T cell receptor (TCR). TCR sequencing will allow a precise evaluation of the development of tumor specific adaptive immune response. To do so, TCR sequences presents in PBMCs and in tumor material will be sequenced and prevalence of tumor specific clones (enriched in the tumor with respect to blood) will be compared before and after vaccination.

Longitudinal assessment of immune or cancer related soluble biomarkers will be performed in peripheral samples. Such biomarkers include cytokines related to immune status, tumor or drug specific makers such as circulating tumor DNA (ctDNA), as well as other soluble factor that will be described in the state-of-the-art at the time of analysis as a prognostic or predictive factor for patient response of clinical outcome.

9. STATISTICAL METHODS PLANNED AND SAMPLE SIZE

The statistical analysis plan for the study includes a more technical and detailed description of the statistical analyses that will apply to all interventions, described in this section. This section is a summary of the planned statistical analyses of the most important endpoints including primary and key secondary endpoints that are evaluated for all interventions in the study.

9.1 Data sets analyzed

9.1.1 Screening period

All patients included will be considered. The percentage of patients for whom TG4050 cannot be manufactured and the reason that prevents its manufacturing will be analyzed.

9.1.2 Treatment period

For the phase I part:

Initial Safety Sets (ISSA and ISSB): the first 3 patients randomized in arm A and in Arm B, respectively who have completed their first 6 weeks of the TG4050 treatment or have discontinued the study treatment due to treatment-related toxicity. These are the primary datasets for first IDMC reviews.

Full Analysis Set (FAS): all patients randomized will be included in the FAS. Following the intent-to-treat principle, patients will be analyzed according to the treatment modality and stratum that they were assigned to at randomization. The FAS will be the primary population for summaries of demography and baseline characteristics and protocol deviations.

Safety Analysis Set (SAF): all patients randomized and who received one dose of TG4050 will be included in the SAF. This is the primary dataset for analyses of safety.

Per-Protocol Sets (PP):

- Evaluable for DFS – DFS-EPP: all randomized patients who completed their first six weeks of TG4050 treatment without recurrence of disease (Arm A) or who had standard medical follow-up according to protocol during the first 6 weeks without recurrence of disease (Arm B). The DFS-EPP is the primary population for DFS analysis.
- Evaluable for PFS – PFS-EPP: all randomized patients who completed their first six weeks of TG4050 treatment after recurrence (both arms) and have CT scan or MRI at recurrence and at least one CT scan or MRI after treatment start post-recurrence with an overall response assessment different from "Unknown" according to RECIST 1.1. The PFS-EPP is the primary population for PFS analysis.

For PP Sets, patients with predefined major protocol deviations that may impact efficacy evaluation will be excluded. Patients are analyzed according to the actual treatment modality and strata.

For phase I/II:

Full Analysis Set (FAS): all patients randomized will be included in the FAS. Following the intent-to-treat principle, patients will be analyzed according to the treatment modality and stratum that they were assigned to at randomization. The FAS will be the primary population for summaries of demography and baseline characteristics and protocol deviations.

Safety Analysis Set (SAF): all patients randomized and who received one dose of TG4050 will be included in the SAF. This is the primary dataset for analyses of safety.

Per-Protocol Sets (PP):

- Evaluable for DFS – DFS-EPP: all randomized patients who completed their first six weeks of TG4050 treatment without recurrence of disease (experimental arm) or who had standard medical follow-up according to protocol during the first 6 weeks without

recurrence of disease (control arm). The DFS-EPP is the primary population for DFS, DMFS and LRFS analyses.

- Evaluable for ORR – ORR-EPP: all patients randomized who completed after recurrence their first six weeks of TG4050 treatment in combination with SOC and have CT scan or MRI at recurrence and at least one CT scan or MRI after treatment start post-recurrence with an overall response assessment different from "Unknown" according to RECIST 1.1. The ORR-EPP is the primary population for ORR analysis.

For PP Sets, patients with predefined major protocol deviations that may impact efficacy evaluation will be excluded. Patients are analyzed according to the actual treatment modality and strata.

9.2 Determination of sample size

Initially, no formal sample size calculation has been performed for the phase I part as First-in-human study.

With the inclusion of additional patients, the purpose of this phase I/II is to evaluate efficacy in terms of Disease-Free survival, with the decision rule being based on the proportion of patients who are still free of disease 2 years after randomization. A sample size of 74 evaluable patients would result in 80% power to detect the difference between a 2Y-DFS of 25% corresponding to 85% in the experimental arm and 60% in the control arm. The sample size calculation is based on one-sided α=0.05 and using un-pooled estimate of variance. Therefore, it is planned to randomize in the phase II part at least 46 patients (23 in each arm) in addition to the 33 patients randomized in the phase I part to obtain a total of at least 74 evaluable patients in this phase I/II trial.

9.3 Study endpoint

9.3.1 Primary endpoint

Phase I part: Adverse events will be assessed throughout the treatment period and graded according to NCI CTCAE version 5.

Phase I/II: Disease-Free Survival calculated from the date of randomization to occurrence of any of the following events, whichever occurs first:

- Locoregional recurrence, or occurrence of distant metastases, or second primary SCCHN, documented clinically and by imaging procedure (determined by the investigator)
- Death from any cause.

DFS will be censored at the date of last evaluable clinical assessment if no recurrence is observed at the cut-off date for analysis.
The proportion of patients free from event at 2 years will be calculated in the two arms.

As a sensitivity analysis, analysis of DFS will be repeated by calculating the time from the date of complete response following surgery and adjuvant therapy to any of the above listed events.

9.3.2 Secondary endpoints

<u>Phase I endpoints:</u>

- Failure to provide rate: percentage of patients for whom TG4050 cannot be provided and the reason for not being able to provide the vaccine will be recorded. Failure to treat rate: percentage of patients who cannot receive TG4050 and the reason that prevent patient from being treated.
- Disease-Free Survival calculated from the date of randomization to occurrence of any of the following events, whichever occurs first:
  - Locoregional recurrence, or occurrence of distant metastases, or second primary SCCHN, documented clinically and by imaging procedure (as determined by the investigator)
  - Death from any cause.

  DFS will be censored at the date of last evaluable clinical assessment if no recurrence is observed at the cut-off date for analysis.

As sensitive analysis, analysis of DFS will be repeated calculating time from the date of complete response following surgery and adjuvant therapy to any of the above listed events.

- Progression Free Survival (PFS) in patients with recurrent cancer calculated from the date of first study treatment administration after recurrence to the date of first documented tumor progression or death due to any cause, whichever occurs first. If a patient has not a PFS event at the cut-off date for analysis or at the date when further antineoplastic therapy (other than those planned as study treatment in the protocol) is started, PFS will be censored at the date of last evaluable tumor assessment before the cut-off or start of further antineoplastic therapy.

As sensitive analysis, analysis of PFS will be repeated calculating time from the date of recurrence documented clinically and by imaging to any of the above listed events.

<u>Phase I/II endpoints</u>

- Distant Metastases-Free Survival (DMFS) calculated from the date of randomization to first occurrence of distant metastasis or death from any cause. DMFS will be censored at the date of last evaluable clinical assessment if no distant metastasis or death is observed at the cut-off date for analysis. DFS-EPP will be the primary dataset for this endpoint.

- Loco-Regional Relapse-Free Survival (LRFS) calculated from the date of randomization to first occurrence of loco-regional recurrence or death from any cause. LRFS will be censored at the date of last evaluable clinical assessment if no loco-regional recurrence or death is observed at the cut-off date for analysis. DFS-EPP will be the primary dataset for this endpoint.

- Overall Response Rate (ORR) in patients with recurrent disease who have started treatment with TG4050 in combination with SOC) defined as the proportion of patients with a best overall response of complete response or partial response according to RECIST, version 1.1. ORR-EPP will be the primary dataset for this endpoint.

- Success rate in providing TG4050 for patients eligible for randomization defined as the proportion of certified individualized IMP among the total number of patients eligible for randomization.

9.3.3 Translational research

Endpoints for the translational research will be:

Phase I part

- Tumor mutational burden (TMB) and neoantigen frequency at Baseline and at recurrence
- Changes in circulating immune cells
- Induction of tumor specific T cells
- T cell changes in tumor immune infiltrate and environment at baseline and at recurrence
- Quantification of tumor and immune-related biomarkers

Phase I/II

- Quantification of tumor and immune-related biomarkers including but not limited to PD-L1 expression, tumor mutational burden (TMB)
- Changes in circulating immune cells
- Induction of antigen specific T cells against neoantigens
- Changes in tumor immune infiltrate and microenvironment at baseline and at recurrence
- Circulating tumor DNA prior and during treatment / observation period

9.4 Statistical and analytical plans

9.4.1 Safety analyses for the treatment period

All safety analyses will be based on the SAF.

Adverse events

The verbatim terms used in the CRF by investigators in the platform study to identify adverse events will be coded using the Medical Dictionary for Regulatory Activities (MedDRA).

AE summaries will include all TEAEs corresponding to AEs that have started on the day of or following the day of first study treatment administration or that have worsened on or after first study administration or have started within 30 days after the date of last study treatment administration. In the phase I part, this corresponds to completion of first series of 20 TG4050 administrations in Arm A and to safety follow-up visit in both arms. In the phase II part, this corresponds to safety follow-up visit in both arms.

For each adverse event preferred term, the number (percentage) of participants who experience at least 1 occurrence of the given event will be summarized by intervention arm. All AEs, significant AEs, AEs leading to discontinuation of study treatment, severe AEs (Grade 3 or 4)

and serious AEs (SAE) will be listed and summarized by System Organ Class (SOC) and Preferred Term (PT), intensity (based on the NCI CTCAE grades, relationship to TG4050).
Written narratives will be produced for all SAEs and unexpected or other significant AEs that are judged to be of special interest because of their clinical importance.
Adverse events/SAEs occurring after signing the Informed Consent Form (ICF) but before starting study treatment, including those observed in patients screened but not included will be listed separately from those occurring after treatment start. Adverse events occurring more than 30 days after last treatment administration will be listed with a flag, if reported.
SAEs evaluated by the investigator as at least possibly related to TG4050 occurring more than 30 days after last administration of TG4050 will be listed separately. (S)ARs related to protocol-required procedures reported until completion of End of Study form will be listed separately.

Fatal Events
All fatal events will be listed and summarized by System Organ Class (SOC) and Preferred Term (PT). The nature of the event leading to death should be recorded. Sudden/unexplained death will be coded as "death".
Fatal events will be summarized by presenting the number and percentage of patients who died. Data will be presented by SOC and PT using MedDRA coding.
Fatal events occurring after signing the Inform Consent Form (ICF) but before starting study treatment in patients screened but not included will be listed separately from those occurring after treatment start.

Laboratory abnormalities
The summaries will include all laboratory assessments collected from baseline assessment until 30 days after treatment discontinuation. In the phase I part, this corresponds to completion of first series of 20 TG4050 administrations in Arm A and to safety follow-up visit in both arms. In the phase II part, this corresponds to safety follow-up visit in both arms. All laboratory assessments will be listed and those collected beyond 30 days after study treatment discontinuation will be flagged in the listings.
All laboratory values will be converted into SI units and will have a severity grade calculated using appropriate common terminology criteria for AEs (NCI-CTCAE, version 5). A listing of laboratory values will be provided by laboratory parameter and patient. A separate listing will display notable laboratory abnormalities (i.e., newly occurring CTCAE Grade 3 or 4 laboratory toxicities). The frequency of these notable laboratory abnormalities will be displayed by parameter. The shift tables using CTCAE grades to compare baseline to the worst post-baseline value will be produced for all relevant safety measures as described in the statistical analysis plan. Note that for parameters with two directions abnormalities (hypo/hyper), two tables will be presented. Laboratory abnormalities occurring after signing the Informed Consent Form (ICF) but before starting study treatment but not included will be listed separately from those occurring after treatment start.

Other safety data

Other safety data (e.g., vital signs, electrocardiogram) will be listed, notable values will be flagged, and any other information collected will be listed as appropriate. Any statistical procedures performed in order to explore the data will be used only to highlight any interesting comparisons that may warrant further consideration.

9.4.2 Failure to provide / to treat

Phase I part

The Failure to provide rate is the number of patients for whom TG4050 cannot be provided over the total number of patients eligible. The reason for not being able to provide the vaccine will be summarized.

The failure to treat rate is the number of patients who cannot receive TG4050 over the total number of patients eligible. The reasons that prevent patient from being treated will be summarized.

Phase I/II

The Success Rate in providing TG4050 for patients eligible for randomization defined as the proportion of certified individualized IMP among the total number of patients eligible for randomization.

9.4.3 Efficacy analyses for the treatment period

The analysis of the efficacy endpoints will be performed on the evaluable population: DFS-EPP (Phase I/II), PFS-EPP (phase I part only), or ORR-EPP (phase I/II), as appropriate. Analyses will be repeated in the FAS (Phase I/II).

Phase I/II

In the phase I/II, the primary objective is to compare the efficacy of TG4050 (experimental arm) and watchful waiting (control arm) based on two-year Disease-Free Survival (DFS) rate.

DFS is defined as the time from randomization until recurrence or death, whichever occurs first. The 2-year DFS probability will be estimated using the Kaplan-Meier method. Patients will be censored at the time of last follow-up provided no event of interest has occurred so that as many patients as possible can be included in the analysis.

- If less than 5% of patients are censored before 2 years, the primary analysis will be conducted using the difference of proportions between the two treatment arms with a 90% confidence interval computed with the Wald method.
- If more than 5% of patients are censored before 2 years, the 2-year DFS rate will be estimated using the Kaplan Meier method, and confidence intervals will be derived using the Greenwood formula.

Hypotheses will be tested at 1-sided α level of 0.05 using z test for the difference of two proportions based on Kaplan-Meier estimates with standard error of each arm computed using Greenwood's formula.

The primary analysis will be performed when at least 74 patients are evaluable based on 33 patients randomized in the phase I part and at least 46 in the phase II part).
As a sensitivity analysis, analysis of DFS will be repeated by calculating the time from the date of complete response following surgery and adjuvant therapy to any of the above listed events.

The difference in the distribution of DFS, DMFS and LRFS between TG4050 vs watchful waiting will be compared via a one-sided, log-rank test and a significance level of 5%, stratified or not by the factors of randomization (excluding center). The hazard ratio and the corresponding 95% CI will be estimated in a Cox proportional hazards model using randomized arm as a single covariate (unstratified) and using randomization factors (stratified). The DFS, DMFS and LRFS curves for each randomized arm will be estimated and plotted using the Kaplan-Meier product limit method. The percentage of patients without event will also be presented over time considering the proportions of patients without event at 12, 18 and 24 months, along with corresponding 95% CIs. Restricted mean of non-relapse time with 95% confidence interval will also be calculated. Tumor response according to RECIST 1.1 will be analyzed in patients with recurrent cancer. ORR will be calculated by arm with associated 95% CIs.

9.4.4 Translational research

A statistical analysis plan dedicated to translational research will be provided separately.

9.5 Methods of analysis

9.5.1 General considerations

Statistical summaries will be produced using SAS® software version 9.4 or higher. Continuous variables will be described using the number of observations (N), arithmetic mean (Mean), standard deviation (sd), minimum (MIN), median (Median), and maximum (MAX). Means will be further described with 95% confidence intervals (CIs) where appropriate. Categorical variables will be summarized by frequency (N) and percentage (%). Proportions will be estimated with their 95% CIs when appropriate.

9.5.2 Disposition of patients

The number of screen failure patients and reasons for screen failure will be summarized for each study period. A patient listing will be provided with the reason of screen failure.

9.5.3 Demographic and baseline characteristics

Demographics and baseline characteristics data will be listed and summarized by treatment arm. Qualitative data will be summarized by means of contingency tables, and quantitative data will be summarized by appropriate descriptive statistics (mean, standard deviation, median, Q1, Q3, minimum, and maximum). Summaries will be provided for each study period and by arm for the treatment period.

9.5.4 Treatments

9.5.4.1 Primary treatment for studied disease

Primary treatment will be listed and summarized (surgery, radiotherapy, chemotherapy).

The FAS set will be used for all summaries and listings of primary treatment.

9.5.4.2 Study treatment

Exposure to TG4050, will be provided by listing and summarizing the number of injections and the duration of treatment.
The SAF will be used for all summaries and listings of study treatment.
The number of patients with dose omission will be presented along with reasons for the dose omission.

9.5.4.3 Concomitant medications

Medications and/or non-drug therapies taken by the patient during the month prior to start of the study treatment (including started and stopped before first study treatment), as well as all medications and significant non-drug therapies taken during the course of the study up to 30 days after the last study treatment administration will be collected with their start date and end date, reason for prescription, route of administration and dose. In the phase I part, 30 days after last study treatment administration correspond to completion of first series of 20 TG4050 administrations in Arm A and to safety follow-up visit in both arms. In the phase II part, this corresponds to safety follow-up visit in both arms.
Medications will be coded using WHO Drug dictionary including the Anatomical Therapeutic Chemical (ATC) classification and will be listed.

- The prior medications started and stopped before the first study treatment will be summarized by ATC by means of frequency counts and percentages.
- The concomitant medications, ongoing at the start of study treatment or taken during the course of the study will be summarized by ATC by means of frequency counts and percentages.

The SAF will be used for all above-mentioned concomitant medication tables.

9.6 Protocol deviations

Any protocol deviations impacting statistical analyses will be classified as major and the list will be described in the statistical analysis plan in order to determine the evaluable population for efficacy. All these protocol deviations for included patients will be presented by patient in data listings with a distinction between deviations at inclusion and deviations during the study. The number of patients with at least one major protocol deviation will be summarized by type and overall, with a distinction between deviations at inclusion and deviations during the study.

10. CHANGES IN THE CONDUCT OF THE STUDY

10.1 Protocol amendments

Changes to this protocol will be performed through amendments issued by Transgene after mutual agreement of the Investigator(s) and Transgene. Both the Investigator(s) and Transgene will sign the amendments. When applicable, amendments are submitted to Health Authorities and the Independent Ethics Committee (IEC(s) and any other committees by Transgene or the sub-contractor or the Coordinating / Principal Investigator according to local regulations.
Authorization / approval will be required before implementation of any change to the protocol which could significantly affect the safety of patients, the scope of the investigation, the scientific quality of the study or any other aspect of the study. Other changes will be provided when required by local laws to IEC(s) and any other committees for information only.

An amendment intended to eliminate an apparent immediate hazard to patients may be implemented immediately. Such an amendment must be notified as soon as possible to Regulatory Authorities IEC(s) and any other committees as locally required for authorization / approval.

10.2 Premature termination or suspension of the study

During the safety run-in phase, the study may be suspended and an IDMC *ad-hoc* meeting immediately called if at least one of the following events occur:

- any treatment-emergent AE grade ≥3 toxicity that is at least possibly related to TG4050, with the following exception: pre-existing grade 2 fatigue at baseline that would worsen up to grade 3
- any death.

Beyond the safety run-in phase, the study may be suspended or terminated under any of the following circumstances:

- Sponsor’s decision
- Health Authorities’ decision
- Any death, unrelated to progressive disease, which occurs within 30 days of TG4050 administration
- IDMC recommendation in case of a safety concern. However, in this case, the final decision on whether to continue the study will be taken by the sponsor.

If the study needs to be terminated, Transgene and the Investigator will assure that adequate consideration is given to the protection of the patients. Transgene will notify the Health Authorities and the IEC(s)/IRB(s), any other committees of the premature study termination according to local regulations.

Should the study be prematurely stopped or put “on-hold” upon Health Authorities’ decision, Transgene will inform immediately the Investigators in written including measures to be implemented.

If the study is prematurely discontinued, all study data must be returned to Transgene. In addition, the site must conduct final disposition of all unused study drugs in accordance with Transgene procedures for the study.

11. ETHICAL CONSIDERATIONS

11.1 Independent Ethics Committee/Institutional Review Board

Before starting the study, the protocol, the written patient information sheet and informed consent form, and any other document specifically requested must be reviewed and approved by an IEC complying with the requirements of relevant local law.
Before enrollment of patients, Transgene must obtain from the IEC(s) a written authorization / approval and the list of members having participated in the meeting including their qualification.

In addition, IEC written approval must be obtained by Transgene for protocol amendment as described in Section 10.1.

11.2 Informed consent

The Investigator or his/her delegate will obtain a voluntary written consent from each patient after an appropriate explanation of the aims, methods, anticipated benefits, risks and any other aspect of the study relevant to the patient's decision to participate. Consent form and all verbal study related information must be in a language fully comprehensible to the prospective patient.
Patients will be informed that they are free not to participate in the study and that they may withdraw consent to participate at any time. They will be told which alternative treatments are available if they refuse to take part and that such refusal will not prejudice future treatment.
Patients will be informed that their records, including medical history, may be examined by competent authorities and authorized persons but that personal information will be treated as strictly confidential and will not be publicly available.
A written "patient information sheet" will be given to each patient to complete the verbal information. This written form should be reviewed orally with the patient. Patient must be given ample opportunity to inquire about details of the study.
The "patient information sheet" will explain that the data collected for this study will be stored in a computer database, with confidentiality maintained in accordance with national data legislation (for instance, the European General Data Protection Regulation for European countries).
Informed consent shall be documented by use of a written consent form approved by the IEC and signed and dated by the Investigator and the patient before undertaking any protocol-specific procedure. Any new version of the ICF will be signed by all ongoing patients.

A copy of the signed informed consent form must be given to the patient and to his/her legally authorized representative.
The original is filed at the study site in the Investigator Site File.

11.3 Confidentiality of patient data

The Investigator must assure that patients' anonymity is maintained and that their identities are protected from unauthorized parties. On eCRFs or other documents collected by Transgene or its representative, patients should not be identified by their names, but by an identification code system.
For European countries:
Confidentiality of patients' medical records will be maintained in accordance with the applicable data privacy laws such as, for instance, the European General Data Protection Regulation (Regulation EU 2016/679 of 27 April 2016 on the protection of natural persons regarding the processing of personal data and on the free movement of such data).

The Investigator should keep a patient identification log showing codes, names and addresses of all patients consented. A copy of this log without names and addresses will be filed at Transgene after study completion.

11.4 Independent Data Monitoring Committee

11.4.1 Responsibilities of the Independent Data Monitoring Committee

Members of the IDMC will be responsible for reviewing primarily the safety data as well as the conduct of the study.

For the phase I part the IDMC will review the safety data of the first 3 treated patients in Arm A and of the first 3 treated patients in Arm B who have completed their first 6 weeks of the TG4050 treatment or have discontinued the study treatment due to treatment-related toxicity. The IDMC will assess/recommend whether it is possible to pursue patient enrolment as per study protocol.
In addition to the reviews of the first 3 patients treated in Arm A and Arm B, respectively the IDMC will review safety data of all patients included every 3 months during the first year and every 6 months thereafter. With the initiation of the phase II part, safety data of patients in the phase I part will be continued to be reviewed at the schedule for the phase II part.
Ad hoc meetings may be held upon necessity.

For the phase II part, the IDMC will review safety data of all patients included every 12 months and at final analysis.
Ad hoc meetings may be held upon necessity.

11.4.2 Notification of the safety reviews to investigators

The outcome(s) of any safety review conducted by the IDMC during the study will be shared with all participating investigators including corrective measures, if appropriate. Timelines of communication to participating investigators will be specified in the IDMC charter. Written and signed recommendations from the IDMC will be available within 24 hours.

Further details on the organization, roles, and responsibilities of this committee will be established in an Independent Data Monitoring Committee charter signed by its members.

12. REGULATORY CONSIDERATIONS

This study will be conducted in accordance with:
- The updated Declaration of Helsinki adopted by the World Medical Association,
- The ICH E6 (R2) (International Conference on Harmonization) Good Clinical Practice (GCP) guidelines, and
- The local regulatory requirements.

12.1 Regulatory approval / authorization

The regulatory authorization / approval for conducting the study will be obtained from Regulatory Authorities in accordance with local regulatory requirements. Additional authorizations / approvals will be obtained from the national gene therapy committees, as required. All approvals must be obtained before a patient is exposed to a study-related procedure, including baseline screening tests for eligibility.

12.2 Investigators' obligations

Before the study starts, the Investigator shall provide Transgene with his/her curriculum vitae and complete a list giving the names, functions and authorized activities of all persons who will exercise any kind of responsibility in carrying out of the study. CVs of these people will also be collected.
The Investigator also provides to the site staff appropriate training. The staff training will be documented in the Investigator Site File.

The Investigator ensures supervising any individual or party to whom trial-related duties and functions conducted at the site have been delegated. When trial-related duties or functions are delegated to parties, the investigator should ensure the individual or party is qualified to perform the delegated tasks and should implement procedures to ensure the integrity of the trial-related duties and functions performed and any data generated.

The Investigator ensures the quality of the study through strict observance of the protocol, Good Clinical Practice and local regulations. Investigator must ensure that the study has been authorized / approved by all Regulatory Authorities, IEC and any other committees prior to enrolling patients and on an ongoing basis as locally required.

Investigator is required to obtain written informed consent from each patient prior a patient is exposed to a study-related procedure, including baseline screening tests for eligibility.

12.3 Insurance

Transgene certifies having taken out a civil liability insurance policy covering liability with regard to the participants in this study.

13. QUALITY CONTROL AND QUALITY ASSURANCE

13.1 On site monitoring

The monitor will contact and visit the Investigator periodically to evaluate study progress and protocol compliance with the first visit occurring as soon as possible after the first patient inclusion. Intervals may be adjusted according to patient accruals, protocol changes or site performance.
On site monitoring activities are based on instructions given within the Monitoring Plan and could be complemented by Central monitoring activities.

During the visit, the Investigator and any study staff member will co-operate with the monitor to ensure that any problems are resolved.

13.2 Audit and inspection

The main purposes of an audit or inspection are to confirm that the rights and well-being of the patients have been adequately protected, and that all data relevant for the evaluation of the IMP have been processed and reported in compliance with GCP and applicable regulatory requirements.
The sponsor is responsible for making sure that both his representatives (monitor, clinical research assistant) and the investigator fulfil their requirements as specified by the GCP guidelines. The audit can be made internally at Transgene and at the investigational site or at the Services Provider. Possibility to have a direct access to all study documentation is compulsory. The practical conditions for the audit will be discussed between the investigator and the Clinical Quality Assurance Department.
The Health Care Authorities may inspect any investigational site or the sponsor during the course of the study or following its completion, to verify the conduct of the study and quality of the data. The investigator will notify TRANSGENE immediately in case of inspection announced by any authority and will share with TRANSGENE any information such as inspection agenda and report upon receipt from the authorities.
After appropriate notification, the Investigator will make all study-related source data and documents available to a quality assurance auditor mandated by Transgene, or to domestic or foreign regulatory inspectors.

14. DATA HANDLING AND RECORD KEEPING

14.1 Source data and documents

Source data are all information available in original source document or certified copies of source document of any clinical findings, observations, or other activities that are necessary for the reconstruction and evaluation of the study.
The Investigator will record at least the following information in the source documents for all consented patients: date of birth, gender, medical history, reference to the study, visit dates, study treatments administrations, concomitant medications, evaluation criteria and nature of

adverse events with date of start and end and relationship to study treatments. The location of each source data will be identified on a dedicated form.

If computerized systems are used to record subjects' source data (i.e. subjects' electronic source documents), the following criteria should be met:

- documented evidence that the computer system has been validated,
- the system provides adequate security to ensure that only authorized people can access the system (log in / password) to enter/change data,
- the system allows audit trail of entries / changes,
- existence of a back-up system,
- existence of data security in case of a system break down,
- the users of the system are trained to the system,
- existence of a written procedure covering these aspects,
- servers containing data are physically protected,
- if an electronic signature is used:
    - the log in and password are entered upon signature
- if no electronic signature is in place, the PI agrees to print out subjects' source data periodically and to sign the printouts.

The Investigator will permit study-related monitoring, audit(s), and regulatory inspection(s), with a direct access to all the required source documents each time it is necessary provided that patient confidentiality is protected.

14.2 Case report forms

Electronic Data Capture (EDC) will be used for this study, meaning that all CRF data will be entered in electronic forms at the investigational site for each screened patient.
All data must be entered in English by the investigator.
The eCRFs should always reflect the latest observations on the patients participating in the study. Therefore, the eCRFs are to be completed without any delay after the patient's visit.
The Investigators must verify that all data entries in the eCRFs are accurate and correct. If some assessments are not done, or if certain information is not available, not applicable, or unknown, the Investigators should indicate this in the eCRF. The Investigators will be required to electronically sign off all the clinical data collected.

The Monitors will review the eCRFs and evaluate them for completeness and consistency. The eCRF will be compared with the source documents to ensure that there are no discrepancies.
All entries, corrections, and alterations are to be made by the Investigator or his/her delegate. Once clinical data have been submitted, corrections to the data fields will be audit trailed, meaning that the reason for change and the name of the person who performed the change, together with time and date, will be logged. Roles and rights of the site personnel responsible for entering the clinical data into the eCRF will be determined in advance and documented on the "delegation form".
If additional corrections or confirmation are needed, the Monitors, Data Manager(s) or authorized Transgene's medical monitors will raise a query.

After database lock, the Principal Investigator will receive a copy of the patient eCRF data for archiving at the investigational site.

15. CLINICAL STUDY REPORT AND PUBLICATION

15.1 Clinical study report

All relevant data will be reported in a clinical study report which will be prepared by Transgene/CRO and submitted for comments and signature to the coordinating / principal Investigators. The final report is used for regulatory purposes by Transgene according to local regulations and provided to each Investigator once finalized.

15.2 Confidentiality of study data

Any information provided by Transgene, including nonclinical data, protocols, eCRFs, verbal and written information, and results of the study will be kept strictly confidential and confined to the clinical personnel involved in conducting this study, and no disclosure shall be made except in accordance with any right of publication granted to the Investigator by Transgene.

15.3 Publication policy

The results of this study may be published or presented at scientific meetings. If this is envisaged, the coauthors agree to submit all manuscripts or abstracts to Transgene prior to scientific meeting or journal submission allowing for reasonable time to review, consistent with Transgene policy. This allows the sponsor to protect proprietary information and to provide medical/scientific review. For intellectual property protection purposes, Transgene can request the coauthors to delay publication or presentation of results.

Consistent with Good Publication Practices (GPP2), authorship is to follow the criteria outlined by the International Committee of Medical Journal Editors (ICMJE), and/or follow the policies outlined by the journal or scientific congress. Financial support for medical writing assistance or travel provided to the authors is also to be acknowledged.

In accordance with consistent editorial practice, Transgene supports the publication of primary study results from multicenter studies in their entirety prior to any secondary analyses. Publication of individual center data unless ancillary study / data is discouraged. A publication in which the contribution of the sponsor's personnel exceeded that of conventional monitoring will be considered for co-authorship provided all other criteria of ICMJE are met.

16. ARCHIVING

16.1 Investigator site file

In accordance with the ICH GCP standards, the Investigator is responsible for on-site storage and maintenance of all records pertaining to the study for the maximum period of time required by local requirements.

No study site document may be destroyed without prior written agreement between the Investigator and Transgene. Transgene must be notified if the Investigator assigns the study documentation to another party or moves it to another location.

If the Investigator cannot guarantee this archiving requirement on site for any or all of the documents, special arrangements must be made between the Investigator and Transgene to store the documents in a sealed container off-site so they can be returned sealed to the Investigator in case of an audit/inspection.

16.2 Trial master file

Transgene will archive the trial master file (TMF) in accordance with GCP and applicable regulatory requirements and will inform the Investigator when the archiving of the study documentation is no longer required.

17. APPENDICES

APPENDIX I	PERFORMANCE STATUS (ECOG) SCALE
APPENDIX II	CONTRACEPTIVE GUIDANCE AND WOMEN OF CHILDBEARING POTENTIAL
APPENDIX IIIA	PHASE I PART AND PHASE II PART: STUDY FLOW CHART FOR SCREENING PERIOD
APPENDIX IIIB	PHASE I PART: STUDY FLOW CHART FOR TREATMENT PERIOD
APPENDIX IIIC	PHASE II PART: STUDY FLOW CHART FOR TREATMENT PERIOD

18. REFERENCES

Ang K.K., Harris J., Wheeler R., Weber R., Rosenthal D.I., Nguyen-Tan P.F., Westra W.H., Chung C.H., Jordan R.C., Lu C., Kim H., Axelrod R., Silverman C.C., Redmond K.P. and Gillison M.L. "Human papillomavirus and survival of patients with oropharyngeal cancer." *The New England journal of medicine*. (2010) 363(1): 24-35.

Bendjama K. and Quemeneur E. "Modified Vaccinia virus Ankara-based vaccines in the era of personalized immunotherapy of cancer." *Hum Vaccin Immunother*. (2017) 13(9): 1997-2003.

Bernier J., Cooper J.S., Pajak T.F., van Glabbeke M., Bourhis J., Forastiere A., Ozsahin E.M., Jacobs J.R., Jassem J., Ang K.K. and Lefebvre J.L. "Defining risk levels in locally advanced head and neck cancers: a comparative analysis of concurrent postoperative radiation plus chemotherapy trials of the EORTC (#22931) and RTOG (# 9501)." *Head Neck*. (2005) 27(10): 843-850.

Bernier J., Domenge C., Ozsahin M., Matuszewska K., Lefebvre J.L., Greiner R.H., Giralt J., Maingon P., Rolland F., Bolla M., Cognetti F., Bourhis J., Kirkpatrick A., van Glabbeke M., European Organization for R. and Treatment of Cancer T. "Postoperative irradiation with or without concomitant chemotherapy for locally advanced head and neck cancer." *N Engl J Med*. (2004) 350(19): 1945-1952.

Bernier J., Vermorken J.B. and Koch W.M. "Adjuvant therapy in patients with resected poor-risk head and neck cancer." *J Clin Oncol*. (2006) 24(17): 2629-2635.

Brana I. and Siu L.L. "Locally advanced head and neck squamous cell cancer: treatment choice based on risk factors and optimizing drug prescription." *Ann Oncol*. (2012) 23 Suppl 10: 178-185.

Cooper J.S., Pajak T.F., Forastiere A.A., Jacobs J., Campbell B.H., Saxman S.B., Kish J.A., Kim H.E., Cmelak A.J., Rotman M., Machtay M., Ensley J.F., Chao K.S., Schultz C.J., Lee N., Fu K.K. and Radiation Therapy Oncology Group I. "Postoperative concurrent radiotherapy and chemotherapy for high-risk squamous-cell carcinoma of the head and neck." *N Engl J Med*. (2004) 350(19): 1937-1944.

Elizaga M.L., Vasan S., Marovich M.A., Sato A.H., Lawrence D.N., Chaitman B.R., Frey S.E. and Keefer M.C. "Prospective surveillance for cardiac adverse events in healthy adults receiving modified vaccinia ankara vaccines: a systematic review." *PLoS One*. (2013) 8(1): e54407.

Kimple R.J. and Harari P.M. "The prognostic value of HPV in head and neck cancer patients undergoing postoperative chemoradiotherapy." *Ann Transl Med*. (2015) 3(Suppl 1): S14.

Nguyen N., Bellile E., Thomas D., McHugh J., Rozek L., Virani S., Peterson L., Carey T.E., Walline H., Moyer J., Spector M., Perim D., Prince M., McLean S., Bradford C.R., Taylor J.M., Wolf G.T., Head and Neck S.P.I. "Tumor infiltrating lymphocytes and survival in patients with head and neck squamous cell carcinoma." *Head Neck*. (2016) 38(7): 1074-1084.

Ottensmeier C., Delord J.P., Jamet C., Lalanne A., Lantz O., Lacoste G., Tavernaro A., Brandely M., Grellier B., Bastien B., Makhloufi H., Huss T., Yamashita Y., Onoue K., Yamagata N., Tanaka Y., Malone B., Baker O., Quemeneur E., Bendjama K., Le Tourneau C. "Safety and Immunogenicity of TG4050: a personalized cancer vaccine in head and neck carcinoma". ASCO 2023 Abstract # 6082

Pagh A., Grau C. and Overgaard J. "Failure pattern and salvage treatment after radical treatment of head and neck cancer." *Acta Oncol*. (2016) 55(5): 625-632.

Quoix E., Lena H., Losonczy G., Forget F., Chouaid C., Papai Z., Gervais R., Ottensmeier C., Szczesna A., Kazarnowicz A., Beck J.T., Westeel V., Felip E., Debieuvre D., Madroszyk A., Adam J., Lacoste G., Tavernaro A., Bastien B., Halluard C., Palanche T. and Limacher J.M. "TG4010 immunotherapy and first-line chemotherapy for advanced non-small-cell lung cancer (TIME): results from the phase 2b part of a randomised, double-blind, placebo-controlled, phase 2b/3 trial." *Lancet Oncol*. (2016) 17(2): 212-223.

Shaw R.J., Lowe D., Woolgar J.A., Brown J.S., Vaughan E.D., Evans C., Lewis-Jones H., Hanlon R., Hall G.L. and Rogers S.N. "Extracapsular spread in oral squamous cell carcinoma." *Head Neck*. (2010) 32(6): 714-722.

Tosch C., Bastien B., Barraud L., Grellier B., Nourtier V., Gantzer M., Limacher J.M., Quemeneur E., Bendjama K. and Preville X. "Viral based vaccine TG4010 induces broadening of specific immune response and improves outcome in advanced NSCLC." *J Immunother Cancer*. (2017) 5(1): 70.

Ward M.J., Thirdborough S.M., Mellows T., Riley C., Harris S., Suchak K., Webb A., Hampton C., Patel N.N., Randall C.J., Cox H.J., Jogai S., Primrose J., Piper K., Ottensmeier C.H., King E.V. and Thomas G.J. "Tumour-infiltrating lymphocytes predict for outcome in HPV-positive oropharyngeal cancer." *Br J Cancer*. (2014) 110(2): 489-500.

Weir G.M., Liwski R.S. and Mansour M. "Immune modulation by chemotherapy or immunotherapy to enhance cancer vaccines." *Cancers (Basel)*. (2011) 3(3): 3114-3142.

Zitvogel L., Apetoh L., Ghiringhelli F. and Kroemer G. "Immunological aspects of cancer chemotherapy." *Nature reviews. Immunology*. (2008) 8(1): 59-73.

Zitzmann-Roth E.M., von Sonnenburg F., de la Motte S., Arndtz-Wiedemann N., von Krempelhuber A., Uebler N., Vollmar J., Virgin G. and Chaplin P. "Cardiac safety of Modified Vaccinia Ankara for vaccination against smallpox in a young, healthy study population." *PLoS One*. (2015) 10(4): e0122653.

APPENDIX I

PERFORMANCE STATUS (ECOG) SCALE

0: Fully active, able to carry on all pre-disease performance without restriction.

1: Restricted in physically strenuous activity but ambulatory and able to carry out work of a light or sedentary nature, e.g. light house work, office work.

2: Ambulatory and capable of self-care but unable to carry out any work activities. Up and about more than 50% of waking hours.

3: Capable of only limited self-care, confined to bed or chair more than 50% of waking hours.

4: Completely disabled. Cannot carry out any self-care. Totally confined to bed or chair.

APPENDIX II

CONTRACEPTIVE GUIDANCE AND WOMEN OF CHILDBEARING POTENTIAL

Birth control methods considered as highly effective

According to the Clinical Trials Facilitation Group (CTFG) "Recommendations related to contraception and pregnancy testing in clinical trials" methods that can achieve a failure rate of less than 1% per year when used consistently and correctly are considered as highly effective birth control methods, such as:

- combined (estrogen and progesterone containing) hormonal contraception associated with inhibition of ovulation[1] (oral, intravaginal, transdermal)
- progesterone-only hormonal contraception associated with inhibition of ovulation[1] (oral, injectable, implantable[2])
- intrauterine device (IUD)[2]
- intrauterine hormone-releasing system (IUS)[2]
- bilateral tubal occlusion[2]
- vasectomized partner[2,3]

[1] Hormonal contraception may be susceptible to interaction with the IMP, which may reduce the efficacy of the contraception method

[2] Contraception methods in the context of this guidance are considered to have low user dependency

[3] Vasectomised partner is a highly effective birth control method provided that the partner is the sole sexual partner of the woman of childbearing potential trial participant and that the vasectomized partner has received medical assessment of the surgical success. If not, an additional highly effective method of contraception should be used.

Woman of Childbearing Potential (WOCBP)

A woman is considered fertile following menarche and until becoming post-menopausal unless permanently sterile. Permanent sterilization methods include hysterectomy, bilateral salpingectomy, and bilateral oophorectomy.

Women in the following categories are not considered WOCBP

1. Premenopausal female with 1 of the following:

   Documented hysterectomy

   Documented bilateral salpingectomy

   Documented bilateral oophorectomy

      Note: Documentation can come from the site personnel's: review of participant's medical records, medical examination, or medical history interview.

2. Premenarchal

3. Postmenopausal female

Postmenopausal state is defined as ≥ 12 months of non-therapy-induced amenorrhea. A high follicle stimulating hormone (FSH) level in the postmenopausal range may be used to confirm a post-menopausal state in women not using hormonal contraception or hormonal replacement therapy (HRT). However, in the absence of 12 months of amenorrhea, a single FSH measurement is insufficient.

Females on HRT and whose menopausal status is in doubt will be required to use one of the non-hormonal highly effective contraception methods if they wish to continue their HRT during the study. Otherwise, they must discontinue HRT to allow confirmation of postmenopausal status before study enrollment.Postmenopausal female or who have undergone hysterectomy or bilateral oophorectomy are exempt from pregnancy testing.

● **Contraceptive Guidance**

**Highly Effective Contraceptive Methods That Are User Dependent**<br>Failure rate of <1% per year when used consistently and correctly.
Combined (estrogen- and progestogen-containing) hormonal contraception associated with inhibition of ovulation[b]<br>oral<br>intravaginal<br>transdermal
Progestogen-only hormonal contraception associated with inhibition of ovulation[b]<br>oral<br>injectable
**Highly Effective Methods That Are User Independent**
Implantable progestogen-only hormonal contraception associated with inhibition of ovulation[b]<br>Intrauterine device (IUD)<br>Intrauterine hormone-releasing system (IUS)<br>bilateral tubal occlusion
Vasectomized partner<br>(A vasectomized partner is a highly effective contraception method provided that the partner is the sole male sexual partner of the WOCBP and the absence of sperm has been confirmed. If not, an additional highly effective method of contraception should be used.
NOTES:<br>a) Typical use failure rates may differ from those when used consistently and correctly. Use should be consistent with local regulations regarding the use of contraceptive methods for participants participating in clinical studies.<br>b) Hormonal contraception may be susceptible to interaction with the study drug, which may reduce the efficacy of the contraceptive method. In this case two highly effective methods of contraception should be utilized during the treatment period and for at least 3 months after the last dose of study treatment

APPENDIX III-A

PHASE I PART AND PHASE II PART:

STUDY FLOW-CHART FOR SCREENING PERIOD

	Before surgery	Completion of surgery	Before adjuvant therapy start (3-12 weeks after surgery)[2]	Completion of adjuvant therapy
**ICF**	**X**			
**Demography, history of SCCHN, relevant medical history**		**X**		**X**
**Inclusion/exclusion criteria check**		**X**		**X**
**Physical examination**		**X**		**X**
**Concomitant medications**		**X**		**X**
**HCV and HBsAg, if applicable**		**X**		
**Blood sampling for ctDNA assessment**	**X**		**X**	**X**[2]
**Blood sampling for gene sequencing**	**X**			
**Pregnancy test**[(1)]		**X**		
**Archived tumor tissue**	**X**[(2)]	**X**		
**Imaging**		**X**		**X** [(*)]

*Check that complete response is documented on CT or MRI of the head and neck and CT-scan of the chest

[(1)] For women of childbearing potential (see definition in Appendix II)

[(2)] in phase II part only

APPENDIX III B

PHASE I PART: STUDY FLOW-CHART FOR TREATMENT PERIOD

Patient in Arm A

	**Baseline**			**First 6 weeks during first and second series of TG4050 administrations**							**Beyond 6 weeks during first and second series of TG4050 administrations**		**Completion of first series of 20 TG4050 administrations**	**Standard medical practice follow-up[15]**	**End of treatment**	**Safety Follow-up**	**Follow-up for progressive disease**	**Long-Term Follow-Up**
	**≤ 21 days prior to randomization**	**≤ 7 days prior to randomization**	**Randomization**	**D1**	**D8 (W1)** +/- 1 day	**D15 (W2)** +/- 1 day	**D22 (W3)** +/- 1 day	**D29 (W4)** +/- 1 day	**D36 (W5)** +/- 1 day	**D43 (W6)** +/- 1 day	**Q3 weeks** +/- 7 days	**Q6 weeks** +/- 7 days	**30 days after last administration of first series of 20 TG4050 administrations**	**Until recurrence**	**At definite discontinuation of study treatment**	**At least 30 days after last study treatment administration (+ 7 days)**	**Until progressive disease[6]**	**Up to 5 years after completion or stop of TG4050 treatment[16]**
**Randomization**			X															
**TG4050 administration**				X	X	X	X	X	X	X	X							
**Inclusion/exclusion criteria check**	X	X																
**Current medical conditions**	X													X[12]				
**Physical examination[1]**	X			X	X	X	X	X	X	X	X			X[12]	X	X		X[16]
**Performance status**	X			X	X	X	X	X	X	X	X				X	X		
**Concomitant medications**	X			X	X	X	X	X	X	X	X		X	X[12]	X	X		
**ECG**	X						X			X		X[10]		X[12]	X			

	**Baseline**			**First 6 weeks during first and second series of TG4050 administrations**							**Beyond 6 weeks during first and second series of TG4050 administrations**		**Completion of first series of 20 TG4050 administrations**	**Standard medical practice follow-up(15)**	**End of treatment**	**Safety Follow-up**	**Follow-up for progressive disease**	**Long-Term Follow-Up**
	**≤ 21 days prior to randomization**	**≤ 7 days prior to randomization**	**Randomization**	**D1**	**D8 (W1)** +/- 1 day	**D15 (W2)** +/- 1 day	**D22 (W3)** +/- 1 day	**D29 (W4)** +/- 1 day	**D36 (W5)** +/- 1 day	**D43 (W6)** +/- 1 day	**Q3 weeks** +/- 7 days	**Q6 weeks** +/- 7 days	**30 days after last administration of first series of 20 TG4050 administrations**	**Until recurrence**	**At definite discontinuation of study treatment**	**At least 30 days after last study treatment administration (+ 7 days)**	**Until progressive disease(6)**	**Up to 5 years after completion or stop of TG4050 treatment(16)**
**Physical examination of Head and Neck as per standard medical practice**	**X**									**X(14)**				**X(14)**				
**AEs/SAEs reporting(7)**	**X**	**X**		**X**	**X**	**X**	**X**	**X**	**X**	**X**	**X**		**X**		**X**	**X**		
**CBC**		**X**			**X**	**X**	**X**	**X**	**X**	**X**	**X(9)**			**X(12)**	**X**			
**Biochemistry**		**X**			**X**	**X**	**X**	**X**	**X**	**X**	**X(9)**			**X(12)**	**X**			
**Serum pregnancy test (hCG)(11)**		**X**					**X**			**X**	**X**		**X**	**X(12)**	**X**	**X**		
**Blood samples+ apheresis for translational research**		**X**		**X(13)**	**X(13)**	**X(2)**				**X(2)**	**X(2)**			**X(2,12)**	**X(2)**		**X(2)**	**X(16)**
**Tumor samples for translational research**														**X(3)**	**X(3)**			
**Skin biopsy**					**X(4)**					**X(4)**								
**Tumor assessment**	**X(8)**									**X(5)**		**X(5)**		**X(5)**			**X(5)**	

(1) Physical examination of the major organ systems, including vital signs (body temperature, pulse rate and blood pressure), and weight

(2) Blood samples for translational research will be collected on days 15, 43, 64, 85, 211 ,then every 12 weeks during the first 24 months after randomization (two timepoints being 18 months and 24 months after start of TG4050 treatment) and every 24 weeks thereafter until recurrence, and at recurrence.After recurrence on R-days 1, 8, 15, 43, starting from R-Day 64 every 12 weeks until progressive disease according to RECIST 1.1, at progressive disease, at End of Treatment, and during Follow-Up for progressive disease (for timepoints, please refer to section 6.2.3), if applicable. Apheresis on day 64 (only during first series of TG4050 treatment)

(3) Tumor samples for translational research will be collected at the time of recurrence & disease progression. A histological/cytological confirmation is required

(4) Skin biopsy to be performed at the injection site on days 8 and 43 in patients with injection site reaction

(5) Tumor assessment by imaging procedures will be performed in the event of clinical signs or symptoms before recurrence; every 6 weeks after recurrence (+/- 7 days)

(6) For patients who completed or prematurely stopped study treatment before documentation of disease progression by imaging

(7) All AEs/SAEs to be reported up to 30 days after last treatment (corresponding to "completion of first series of 20 TG4050 administrations" and to "safety follow-up visit" after definitive stop of study treatments), only SAEs at least possibly related to IMP and (S)ARs related to protocol-required procedures thereafter

(8) Not to be repeated if the previous imaging has been performed within 3 weeks prior to randomization

(9) CBC including Red Blood cells (RBC), White Blood cells (WBC) and differential, Hemoglobin (Hb), and platelets & Biochemistry every week up to week 6, then every 3 weeks up to Month 6 (Day 190), thereafter every 12 weeks

(10) ECG every 6 weeks up to Month 6 (Day 190), thereafter every 12 weeks

(11) For women of childbearing potential (see definition in Appendix II)

(12) At recurrence

(13) Only on R-Days

(14) On Day 43 and then every 12 weeks during the first 24 months after randomization and every 24 weeks thereafter: physical examination of the head and neck including panendoscopy if appropriate, MRI or CT of the head and neck in the event of clinical signs or symptoms of recurrence as per standard medical practice

(15) For patients who completed or prematurely stopped the study treatment before documentation of recurrence clinically and by imaging

(16) 6 months after end of treatment, 12 months after end of treatment and then yearly assessment until 5 years after end of treatment including Clinical history (any relevant clinical events [e.g., immune disease]), a physical examination and blood sampling for immune response to the vaccine

Patient in Arm B

	Baseline			Standard medical practice follow-up	First 6 weeks during TG4050 administrations							Beyond 6 weeks during TG4050 administrations		End of treatment	Safety Follow-up	Follow-up for progressive disease	Long-Term Follow-Up
	**≤ 21 days prior to randomization**	**≤ 7 days prior to randomization**	**Randomization**	**Until recurrence**	**R-D1**	**R-D8 (W1)** +/- 1 day	**R-D15 (W2)** +/- 1 day	**R-D22 (W3)** +/- 1 day	**R-D29 (W4)** +/- 1 day	**R-D36 (W5)** +/- 1 day	**R-D43 (W6)** +/- 1 day	**Q3 weeks** +/- 7 days	**Q6 weeks** +/- 7 days	**At definite discontinuation of study treatment**	**At least 30 days after last study treatment administration (+ 7 days)**	**Until progressive disease(6)**	**Up to 5 years after completion or stop of TG4050 treatement (14)**
**Randomization**			X														
**TG4050 administration**					X	X	X	X	X	X	X	X					
**Inclusion / exclusion criteria check**	X	X															
**Current medical conditions**	X			X(12)													
**Physical examination(1)**	X			X(12)	X	X	X	X	X	X	X	X		X	X		X(14)
**Performance status**	X				X	X	X	X	X	X	X	X		X	X		
**Concomitant medications**	X			X(12)	X	X	X	X	X	X	X	X		X	X		
**ECG**	X			X(12)				X			X		X(10)	X			
**Physical examination of Head and Neck as per standard medical practice**	X			X(13)													
**AEs/SAEs reporting(7)**	X	X		X(12)	X	X	X	X	X	X	X	X		X	X		
**CBC**		X		X(12)		X	X	X	X	X	X	X(9)		X			

	**Baseline**			**Standard medical practice follow-up**	**First 6 weeks during TG4050 administrations**							**Beyond 6 weeks during TG4050 administrations**		**End of treatment**	**Safety Follow-up**	**Follow-up for progressive disease**	**Long-Term Follow-Up**
	**≤ 21 days prior to randomization**	**≤ 7 days prior to randomization**	**Randomization**	**Until recurrence**	**R-D1**	**R-D8 (W1)** +/- 1 day	**R-D15 (W2)** +/- 1 day	**R-D22 (W3)** +/- 1 day	**R-D29 (W4)** +/- 1 day	**R-D36 (W5)** +/- 1 day	**R-D43 (W6)** +/- 1 day	**Q3 weeks** +/- 7 days	**Q6 weeks** +/- 7 days	**At definite discontinuation of study treatment**	**At least 30 days after last study treatment administration (+ 7 days)**	**Until progressive disease[6]**	**Up to 5 years after completion or stop of TG4050 treatement [14]**
**Biochemistry**		X		X[12]		X	X	X	X	X	X	X[9]		X			
**Serum pregnancy test (hCG))[11]**		X		X[12]				X			X	X		X	X		
**Blood samples + apheresis for translational research**		X		X[2,12]	X[2]	X[2]	X[2]				X[2]	X[2]		X[2]		X[2]	X[14]
**Tumor samples for translational research**				X[3]										X[3]			
**Skin biopsy**						X[4]					X[4]						
**Tumor assessment**	X[8]			X[5]							X[5]		X[5]			X[5]	

(1) Physical examination of the major organ systems, including vital signs (body temperature, pulse rate and blood pressure), and weight

(2) Blood samples for translational research will be collected during Standard medical practice follow-up every 12-weeks during the first 24 months after randomization (two timepoints being 18 months and 24 months after randomization) and every 24 weeks thereafter until recurrence, and at recurrence. After recurrence on R-days 1, 8 15, 43, starting from R-Day 64 every 12 weeks until progressive disease according to RECIST 1.1, at progressive disease, at End of Treatment, and during Follow-Up for progressive disease (for timepoints, please refer to section 6.2.3), if applicable. Apheresis on R-day 64

(3) Tumor samples for translational research will be collected at the time of recurrence & disease progression. A histological/cytological confirmation is required

(4) Skin biopsy to be performed at the injection site on days 8 and 43 in patients with injection site reaction

(5) Tumor assessment by imaging procedures will be performed in the event of clinical signs or symptoms before recurrence; every 6 weeks after recurrence (+/- 7 days)

(6) For patients who completed or prematurely stopped study treatment before documentation of disease progression by imaging

(7) All AEs/SAEs to be reported up to 30 days after last treatment (corresponding to safety follow-up visit after definitive stop of study treatments), only SAEs at least possibly related to IMP and (S)ARs related to protocol-required procedures thereafter

(8) Not to be repeated if the previous imaging has been performed within 3 weeks prior to randomization

(9) CBC including Red Blood cells (RBC), White Blood cells (WBC) and differential, Hemoglobin (Hb), and platelets & Biochemistry every week up to week 6, then every 3 weeks up to Month 6 (Day 190), thereafter every 12 weeks

(10) ECG every 6 weeks up to Month 6 (Day 190), thereafter every 12 weeks

(11) For women of childbearing potential (see definition in Appendix II)

(12) At recurrence

(13) On Day 43 and then every 12 weeks during the first 24 months after randomization and every 24 weeks thereafter: physical examination of the head and neck including panendoscopy if appropriate, MRI or CT of the head and neck in the event of clinical signs or symptoms of recurrence as per standard medical practice

(14) 6 months after end of treatment, 12 months after end of treatment and then yearly assessment until 5 years after end of treatment including Clinical history (any relevant clinical events [e.g., immune disease]), a physical examination and a blood sampling for immune response to the vaccine

APPENDIX III C

PHASE II PART: STUDY FLOW-CHART FOR TREATMENT PERIOD

<u>Patient in Experimental Arm</u>

	Baseline			First 6 weeks during TG4050 administrations							Beyond 6 weeks during TG4050 administrations			End of treatment	Safety Follow-up	At recurrence	Follow-up for recurrence	Long-Term Follow-Up
	≤ 21 days prior to randomization	≤ 7 days prior to randomization	Randomization	D1	D8 (W1) +/- 1 day	D15 (W2) +/- 1 day	D22 (W3) +/- 1 day	D29 (W4) +/- 1 day	D36 (W5) +/- 1 day	D43 (W6) +/- 1 day	Q3 weeks +/- 7 days	Q6 weeks +/- 7 days	Q12 weeks +/- 7 days	At definite discontinuation with or without recurrence	At least 30 days after last study treatment administration (+ 7 days)		Standard medical practice [13] Until recurrence	Up to 5 years after completion or stop of TG4050 treatment [14]
**Randomization**			X															
**TG4050 administration**				X	X	X	X	X	X	X	X							
**Inclusion/exclusion criteria check**	X	X																
**Current medical conditions**	X																	
**Physical examination[1]**	X			X	X	X	X	X	X	X	X			X	X			X[14]
**Performance status**	X			X	X	X	X	X	X	X	X			X	X			
**Concomitant medications**	X			X	X	X	X	X	X	X	X			X	X			
**ECG[10]**	X						X			X		X[10]		X				
**Physical examination of Head and Neck as per standard medical practice**	X									X[3]			X[3]				X[3]	

	**Baseline**			**First 6 weeks during TG4050 administrations**							**Beyond 6 weeks during TG4050 administrations**			**End of treatment**	**Safety Follow-up**	**At recurrence**	**Follow-up for recurrence**	**Long-Term Follow-Up**
	**≤ 21 days prior to randomization**	**≤ 7 days prior to randomization**	**Randomization**	**D1**	**D8 (W1)** +/- 1 day	**D15 (W2)** +/- 1 day	**D22 (W3)** +/- 1 day	**D29 (W4)** +/- 1 day	**D36 (W5)** +/- 1 day	**D43 (W6)** +/- 1 day	**Q3 weeks** +/- 7 days	**Q6 weeks** +/- 7 days	**Q12 weeks** +/- 7 days	**At definite discontinuation with or without recurrence**	**At least 30 days after last study treatment administration (+ 7 days)**		**Standard medical practice (13)** **Until recurrence**	**Up to 5 years after completion or stop of TG4050 treatment (14)**
**AEs/SAEs reporting(7)**	X	X		X	X	X	X	X	X	X	X			X	X			
**CBC(9)**		X			X	X	X	X	X	X	X(9)			X				
**Biochemistry(9)**		X			X	X	X	X	X	X	X(9)			X				
**Serum pregnancy test (hCG)(11)**		X					X			X	X			X	X			
**Blood samples for translational research**		X				X(2)				X(2)	**Day 64** X		X(2)	X		X	X(2)	X(14)
**Apheresis**		X(4)									**Day 64** X(4)							
**Tumor samples for translational research**														X(5, 12)		X(5)		
**Tumor assessment**	X(8)									X(6)		X(6)					X(6)	

(1) Physical examination of the major organ systems, including vital signs (body temperature, pulse rate and blood pressure), and weight

(2) Blood samples for translational research will be collected on days 15, 43, starting from 64 every 12 weeks during the first 24 months after randomization (two timepoints being 18 months and 24 months after start of TG4050 treatment) and every 24 weeks thereafter, at End of Treatment, at recurrence, and during Follow-up for recurrence (for timepoints please refer to section 6.2.7), if applicable

(3) On Day 43 and then every 12 weeks during the first 24 months after randomization and every 24 weeks thereafter: physical examination of the head and neck including panendoscopy if appropriate, MRI or CT of the head and neck in the event of clinical signs or symptoms of recurrence as per standard medical practice

(4) In selected centers, apheresis for collection of PBMC will be performed at Baseline and after 9 weeks of treatment, corresponding to Day 64. If clinically not feasible, the apheresis procedure can be replaced by a 150 ml blood draw. In centers not selected for apheresis procedure, a 150 ml blood draw is mandatory at Baseline and after 9 weeks of treatment

(5) Tumor samples for translational research will be collected at the time of recurrence. A histological/cytological confirmation is required

(6) Tumor assessment by imaging procedures will be performed in the event of clinical signs or symptoms until recurrence

(7) All AEs/SAEs to be reported up to 30 days after last treatment corresponding to "safety follow-up visit", only SAEs at least possibly related to IMP and (S)ARs related to protocol-required procedures thereafter

(8) Not to be repeated if the previous imaging has been performed within 3 weeks prior to randomization

(9) CBC (including Red Blood cells [RBC], White Blood cells [WBC] and differential, Hemoglobin [Hb), and platelets) & Biochemistry every week up to week 6, then every 3 weeks up to Month 6 (Day 190), thereafter every 12 weeks

(10) ECG every 6 weeks up to Month 6 (Day 190), thereafter every 12 weeks

(11) For women of childbearing potential (see definition in Appendix II)

(12) If End of treatment is at recurrence

(13) For patients who completed or prematurely stopped the study treatment before documentation of recurrence by imaging

(14) 6 months after end of treatment, 12 months after end of treatment and then yearly assessment until 5 years after end of treatment including Clinical history (any relevant clinical events [e.g., immune disease]), a physical examination and a blood sampling for immune response to the vaccine

Patient in Control Arm

	**Baseline**			**Standard medical practice follow-up**	**At recurrence**	**First 6 weeks during TG4050 administrations**							**Beyond 6 weeks during TG4050 administrations**			**End of treatment**	**Safety Follow-up**	**Follow-up for progressive disease**	**Long-Term Follow-Up**
	**≤ 21 days prior to randomization**	**≤ 7 days prior to randomization**	**Randomization**			**R-D1**	**R-D8 (W1)** +/- 1 day	**R-D15 (W2)** +/- 1 day	**R-D22 (W3)** +/- 1 day	**R-D29 (W4)** +/- 1 day	**R-D36 (W5)** +/- 1 day	**R-D43 (W6)** +/- 1 day	**Q3 weeks** +/- 7 days	**Q6 weeks** +/- 7 days	**Q12 weeks** +/- 7 days	**At definite discontinuation of study treatment**	**At least 30 days after last study treatment administration (+ 7 days)**	**Until progressive disease(8)**	**Up to 5 years after completion or stop of TG4050 treatment(14)**
**Randomization**			X																
**TG4050 administration**						X	X	X	X	X	X	X	X						
**Inclusion / exclusion criteria check**	X	X																	
**Current medical conditions**	X				X														
**Physical examination(1)**	X					X	X	X	X	X	X	X	X			X	X		X(14)
**Performance status**	X					X	X	X	X	X	X	X	X			X	X		
**Concomitant medications**	X				X	X	X	X	X	X	X	X	X			X	X		
**ECG**	X				X				X			X		X(12)		X			
**Physical examination of Head and Neck as per standard medical practice**	X			X(4)															
**AEs/SAEs reporting(9)**	X	X				X	X	X	X	X	X	X	X			X	X		

	**Baseline**			**Standard medical practice follow-up**	**At recurrence**	**First 6 weeks during TG4050 administrations**							**Beyond 6 weeks during TG4050 administrations**			**End of treatment**	**Safety Follow-up**	**Follow-up for progressive disease**	**Long-Term Follow-Up**
	**≤ 21 days prior to randomization**	**≤ 7 days prior to randomization**	**Randomization**			**R-D1**	**R-D8 (W1)** +/- 1 day	**R-D15 (W2)** +/- 1 day	**R-D22 (W3)** +/- 1 day	**R-D29 (W4)** +/- 1 day	**R-D36 (W5)** +/- 1 day	**R-D43 (W6)** +/- 1 day	**Q3 weeks** +/- 7 days	**Q6 weeks** +/- 7 days	**Q12 weeks** +/- 7 days	**At definite discontinuation of study treatment**	**At least 30 days after last study treatment administration (+ 7 days)**	**Until progressive disease[8]**	**Up to 5 years after completion or stop of TG4050 treatment[14]**
**CBC**		X			X		X	X	X	X	X	X	X[11]			X			
**Biochemistry**		X			X		X	X	X	X	X	X	X[11]			X			
**Serum pregnancy test (hCG))[13]**		X			X				X			X	X			X	X		
**Blood samples for translational research**		X		X[2]	X	X	X	X				X	R- Day 64 X		X[3]	X		X[3]	X[14]
**Apheresis**		X[5]											R- Day 64 X[5]						
**Tumor samples for translational research**					X[6]														
**Tumor assessment**	X[10]			X[7]								X[7]		X[7]				X[7]	

(1) Physical examination of the major organ systems, including vital signs (body temperature, pulse rate and blood pressure), and weight

(2) Before recurrence blood samples for translational research will be collected on Day 15, on Day 43, starting from Day 64 every 12 weeks during the first 24 months after randomization (two timepoints being 18 months and 24 months after randmization) and every 24 weeks thereafter and at recurrence

(3) After recurrence blood samples for translational research will be collected on R-Day 1, 8, 15, 43, starting from R-Day 64 every 12 weeks until progressive disease according to RECIST 1.1, at progressive disease, and during Follow-up for progressive disease (for timepoints please refer to section 6.2.7), if applicable

(4) On Day 43 and then every 12 weeks during the first 24 months after randomization and every 24 weeks thereafter: physical examination of the head and neck including panendoscopy if appropriate, MRI or CT of the head and neck in the event of clinical signs or symptoms of recurrence as per standard medical practice

(5) In selected centers, apheresis for collection of PBMC will be performed at Baseline and after 9 weeks of treatment, corresponding to R-Day 64. If clinically not feasible, the apheresis procedure can be replaced by a 150 ml blood draw. In centers not selected for apheresis procedure, a 150 ml blood draw is mandatory at Baseline and after 9 weeks of treatment

(6) Tumor samples for translational research will be collected at the time of recurrence. A histological/cytological confirmation is required

(7) Tumor assessment by imaging procedures will be performed in the event of clinical signs or symptoms before recurrence. After recurrence for patients who have started treatment with TG4050 in combination with SOC: every 6 weeks (+/- 7 days) imaging until documented disease progression according to RECIST 1.1

(8) For patients who completed or prematurely stopped study treatment before documentation of disease progression according to RECIST 1.1

(9) All AEs/SAEs to be reported up to 30 days after last treatment corresponding to "safety follow-up visit", only SAEs at least possibly related to IMP and (S)ARs related to protocol-required procedures thereafter

(10) Not to be repeated if the previous imaging has been performed within 3 weeks prior to randomization

(11) CBC (including Red Blood cells [RBC], White Blood cells [WBC] and differential, Hemoglobin [Hb], and platelets) & Biochemistry every week up to week 6, then every 3 weeks up to Month 6 (R-Day 190), thereafter every 12 weeks

(12) ECG every 6 weeks up to Month 6 (R-Day 190), thereafter every 12 weeks

(13) For women of childbearing potential (see definition in Appendix II)

(14) 6 months after end of treatment, 12 months after end of treatment and then yearly assessment until 5 years after end of treatment including Clinical history (any relevant clinical events [e.g., immune disease]), a physical examination, and blood sampling for immune response to the vaccine
