# Supplementary material for: A viral-based individualized neoantigen vaccine as adjuvant treatment in resected head and neck squamous cell carcinoma: a randomized Phase I trial
Source: Nat Commun. 2026 Aug 17;17:9863. doi: 10.1038/s41467-026-76667-1 (PMC13578212; doi:10.1038/s41467-026-76667-1)
Supplement: Supplementary file 2 [file 41467_2026_76667_MOESM2_ESM.pdf]

Title: Supplementary Data 1.

Description: E*x vivo* IFN-γ ELISpot raw data and definition of responses

Title: Supplementary Data 2.

Description: Differential gene expression analysis between neoantigen-specific $CD8^+$ T cells and virus-specific (MVA, CMV and flu) $CD8^+$ T cells analyzed by scRNAseq

Title: Supplementary Data 3.

Description: Clonotypic analysis of neoantigen-specific $CD8^+$ T cells and virus-specific (MVA, CMV and flu) $CD8^+$ T cells analyzed by scRNAseq
