# Supplementary material for: A viral-based individualized neoantigen vaccine as adjuvant treatment in resected head and neck squamous cell carcinoma: a randomized Phase I trial
Source: Nat Commun. 2026 Aug 17;17:9863. doi: 10.1038/s41467-026-76667-1 (PMC13578212; doi:10.1038/s41467-026-76667-1)
Supplement: Supplementary file 3 [file 41467_2026_76667_MOESM6_ESM.pdf]

nature portfolio

Corresponding author(s): Christian Ottensmeier

Last updated by author(s): Jul 27, 2026

Reporting Summary

Nature Portfolio wishes to improve the reproducibility of the work that we publish. This form provides structure for consistency and transparency in reporting. For further information on Nature Portfolio policies, see our Editorial Policies and the Editorial Policy Checklist.

Statistics

For all statistical analyses, confirm that the following items are present in the figure legend, table legend, main text, or Methods section.

n/a	Confirmed	
☐	☒	The exact sample size (*n*) for each experimental group/condition, given as a discrete number and unit of measurement
☐	☒	A statement on whether measurements were taken from distinct samples or whether the same sample was measured repeatedly
☐	☒	The statistical test(s) used AND whether they are one- or two-sided<br>*Only common tests should be described solely by name; describe more complex techniques in the Methods section.*
☐	☒	A description of all covariates tested
☒	☐	A description of any assumptions or corrections, such as tests of normality and adjustment for multiple comparisons
☐	☒	A full description of the statistical parameters including central tendency (e.g. means) or other basic estimates (e.g. regression coefficient) AND variation (e.g. standard deviation) or associated estimates of uncertainty (e.g. confidence intervals)
☐	☒	For null hypothesis testing, the test statistic (e.g. *F*, *t*, *r*) with confidence intervals, effect sizes, degrees of freedom and *P* value noted<br>*Give P values as exact values whenever suitable.*
☒	☐	For Bayesian analysis, information on the choice of priors and Markov chain Monte Carlo settings
☒	☐	For hierarchical and complex designs, identification of the appropriate level for tests and full reporting of outcomes
☒	☐	Estimates of effect sizes (e.g. Cohen's *d*, Pearson's *r*), indicating how they were calculated

Our web collection on statistics for biologists contains articles on many of the points above.

Software and code

Policy information about availability of computer code

Data collection	This study used the Ennov Clinical v8.2.300 Electronic Data Capture System for data collection.
Data analysis	Statistical analysis of clinical data: SAS version 9.4 (SAS Institute, Inc., Cary, NC, USA), amapping and variant calling, and neoantigen peptides identification: proprietary custom bioinformatics analysis - NEC ; VacDesignR®, design of the personalized vaccine cassettes: a proprietary vaccine design software developed by Transgene SA, flow cytometry: FlowJo, version 10.10.0; single cell data processing with Cellranger (version 8.0.1); analyses with R language (version 4.4.1), Seurat R package (version 5.2.1), ggplot2 R package (3.5.1)

For manuscripts utilizing custom algorithms or software that are central to the research but not yet described in published literature, software must be made available to editors and reviewers. We strongly encourage code deposition in a community repository (e.g. GitHub). See the Nature Portfolio guidelines for submitting code & software for further information.

Data

Policy information about availability of data

All manuscripts must include a data availability statement. This statement should provide the following information, where applicable:

- Accession codes, unique identifiers, or web links for publicly available datasets
- A description of any restrictions on data availability
- For clinical datasets or third party data, please ensure that the statement adheres to our policy

The study protocol is available as Supplementary Information. De-identified patient data supporting the findings of this study are included in the Source Data files

provided with this paper. Raw scRNAseq data are publicly available in the Gene Expression Omnibus (GEO) database under accession number GSE327189 (https://www.ncbi.nlm.nih.gov/geo/query/acc.cgi?acc=GSE327189), with sample correspondence in Supplementary Table 4. Patient WES and RNAseq data used for vaccine design will not be shared due to patient consent and data privacy laws.

Research involving human participants, their data, or biological material

Policy information about studies with human participants or human data. See also policy information about sex, gender (identity/presentation), and sexual orientation and race, ethnicity and racism.

Reporting on sex and gender	Gender (male / female) has been collected in the eCRFand is based on self-reporting.<br>Gender has not been considered neither in the study design nor in analyses. As the presented trial is a phase I trial with a very limited number of participants it was not deemed approriate to perform gender based analyses.<br>Written informed consent has been obtained to share pseudonymized individual-level data.<br>Summarized gender information (Male no [%]) is given in Table 1: Baseline characteristics in the intent-to-treat (ITT) and per-protocol populations.<br>Within the 33 patients randomized, 9 were female (6 in Arm A, 3 in Arm B) and 24 were male (11 in Arm A and 13 in Arm B).
Reporting on race, ethnicity, or other socially relevant groupings	Race, ethnicity or other socially relevant groupings have not been reported in the eCRF.
Population characteristics	Information on age, gender, alcohol and tobacco consumption, ECOG performance status, medical history and concomitant medications, as well as disease specific characteristics haven been collected. Disease specific characteristics included primary tumor site, clinical and pathological stage, risk factors identified during pathologist review, and completed primary treatment for locally advanced disease. No subanalyses based on population characteristics were conducted due to the limited sample size in this phase 1 trial.
Recruitment	Patients were recruited by the clinical trial center per eligibility criteria outlined in the manuscript and detailed in the clinical trial protocol. All patients meeting the eligibility requirements were considered for enrollment regardless of gender or age. Prior to any nonroutine analysis, sampling or intervention, each patient was fully informed about the study, and signed an informed consent form. Patients were not compensated for participating in the trial.
Ethics oversight	The trial was conducted in accordance with the Declaration of Helsinki and Good Clinical Practice guidelines.<br>• France : Comté de Protection des Personnes Ile de France 5, ID: 35725 (EUCT 2023-508561-33); Initial approval on 01 Oct 2019<br>• UK : South Central - Oxford A Research Ethics Committee, ID: 19/SC/0500 (EudraCT 2018-003267-58); Initial approval on 26 Mar 2020<br>• US : Mayo Clinic Institutional Review Boards, ID: 20-007421; Initial approval on 19 Feb 2021

Note that full information on the approval of the study protocol must also be provided in the manuscript.

Field-specific reporting

Please select the one below that is the best fit for your research. If you are not sure, read the appropriate sections before making your selection.

☒ Life sciences ☐ Behavioural & social sciences ☐ Ecological, evolutionary & environmental sciences

For a reference copy of the document with all sections, see nature.com/documents/nr-reporting-summary-flat.pdf

Life sciences study design

All studies must disclose on these points even when the disclosure is negative.

Sample size	We targeted to randomize up to 40 patients. No power calculations for sample size considerations have been performed for this study that employed descriptive and exploratory analyses and had no planned hypothesis testing.
Data exclusions	No data was excluded
Replication	Clinical samples were analyzed longitudinally. As many of the biological samples used for translational analyses were obtained on trial, capacity for replications was limited by the availability of biological samples. Triplicates were performed as feasible (e.g. x vivo IFN-γ ELISpot analysis).
Randomization	Randomization was performed based on a dynamic method (minimization) according to pathological tumor stage (pIII vs. pIV), cisplatin use, and center
Blinding	This was an open label non blinded study.

Reporting for specific materials, systems and methods

We require information from authors about some types of materials, experimental systems and methods used in many studies. Here, indicate whether each material, system or method listed is relevant to your study. If you are not sure if a list item applies to your research, read the appropriate section before selecting a response.

Materials & experimental systems

n/a	Involved in the study
☐	☒ Antibodies
☐	☒ Eukaryotic cell lines
☒	☐ Palaeontology and archaeology
☒	☐ Animals and other organisms
☐	☒ Clinical data
☒	☐ Dual use research of concern
☒	☐ Plants

Methods

n/a	Involved in the study
☒	☐ ChIP-seq
☐	☒ Flow cytometry
☒	☐ MRI-based neuroimaging

Antibodies

Antibodies used

CD45RA FITC BD 555488
CD39 Percp-ef710 eBioscience 46-0399-42
PD-1 APC-CY7 Biolegend 329922
CCR7 BV421 Biolegend 353208
CD27 PC7 BD 560609
CD8 BUV396 BD 563795
CD3 BUV737 BD 612751
PE Streptavidin BD 554061
Brilliant Violet 711 Streptavidin BIOLEGEND 405241
Brilliant Violet 605 Streptavidin BIOLEGEND 405229
Brilliant Violet 650 Streptavidin BIOLEGEND 405231
PE-CF594 Streptavidin BD 562284
PE-Cy5 Streptavidin BD 554062
APC Streptavidin BD 554067

Validation

Antibodies listed are for research use but were listed in multiple publications.

Eukaryotic cell lines

Policy information about cell lines and Sex and Gender in Research

Cell line source(s)

Primary chicken embryo fibroblasts (CEFs)

Authentication

These cells were used for TG4050 production under GMP conditions

Mycoplasma contamination

These cells were negative for mycoplasma contamination

Commonly misidentified lines
(See ICLAC register)

Name any commonly misidentified cell lines used in the study and provide a rationale for their use.

Clinical data

Policy information about clinical studies

All manuscripts should comply with the ICMJE guidelines for publication of clinical research and a completed CONSORT checklist must be included with all submissions.

Clinical trial registration

NCT04183166; EUCT 2023-508561-33; EudraCT 2018-003267-58

Study protocol

The clinical study protocol is provided redacted for personal information as a Supplementary Information file.

Data collection

Thirty-three (33) participants were randomized between January 2021 and April 2023 across 3 sites in France and the United Kingdom. Data cut-off for primary efficacy data was 22 April 2025. An additional efficacy analysis was performed on 31 March 2026 after a median follow-up of 41 months.

Outcomes

Primary objective was evaluation of safety. Dose-limiting toxicities (DLTs) were defined as as any treatment-emergent Adverse Event (AE) grade ≥3 toxicity that is at least possibly related to TG4050, with the exception of pre-existing grade 2 fatigue at baseline that would worsen up to grade 3, and any death during the Safety run-in phase (first 3 treated patients in each arm observed during 6 weeks after the first administration). Beyond the Safety run-in phase DLTs were defined as any treatment-emergent AE ≥3 toxicity that is at least possibly related to TG4050, with the exceptions of pre-existing grade 2 fatigue at baseline that would worsen up to grade 3. Endpoints were Adverse Events (AEs), serious AEs (SAEs) and AEs leading to treatment discontinuation, as well as changes in laboratory values based on CTCAE v4.3, physical examination findings. The secondary objective of the trial was the evaluation of antitumor activity in terms of Disease Free Survival (DFS) calculated from the date of randomization to occurrence of any of the following events, whichever occurs first: Locoregional recurrence, occurrence of distant metastases, second primary HNSCC, or death

from any cause. Other secondary objectives included overall response rate based (ORR), and progression free survival (PFS) based on RECIST 1.1 in patients with recurrent cancer. The secondary objective Failure to provide TG4050 was calculated based on the number of productions for eligible patients.

Plants

Seed stocks

Report on the source of all seed stocks or other plant material used. If applicable, state the seed stock centre and catalogue number. If plant specimens were collected from the field, describe the collection location, date and sampling procedures.

Novel plant genotypes

Describe the methods by which all novel plant genotypes were produced. This includes those generated by transgenic approaches, gene editing, chemical/radiation-based mutagenesis and hybridization. For transgenic lines, describe the transformation method, the number of independent lines analyzed and the generation upon which experiments were performed. For gene-edited lines, describe the editor used, the endogenous sequence targeted for editing, the targeting guide RNA sequence (if applicable) and how the editor was applied.

Authentication

Describe any authentication procedures for each seed stock used or novel genotype generated. Describe any experiments used to assess the effect of a mutation and, where applicable, how potential secondary effects (e.g. second site T-DNA insertions, mosiacism, off-target gene editing) were examined.

Flow Cytometry

Plots

Confirm that:

☒ The axis labels state the marker and fluorochrome used (e.g. CD4-FITC).

☒ The axis scales are clearly visible. Include numbers along axes only for bottom left plot of group (a 'group' is an analysis of identical markers).

☒ All plots are contour plots with outliers or pseudocolor plots.

☒ A numerical value for number of cells or percentage (with statistics) is provided.

Methodology

Sample preparation

PBMCs were thawed in RPMI medium containing 10% FBS, 1% penicillin/streptomycin, and DNase (Merck; ref: DN25-100mg) at 10 µg/mL, then centrifuged at 300g for 8 minutes. The cell pellet was resuspended in medium with dasatinib at 500 nM and incubated for 30 minutes at 37°C. After incubation, cells were filtered through a 40 µm cell strainer and counted. CD8+ T cell enrichment was performed using the EasySep Negative Human CD8+ Enrichment Kit (StemCell; ref: 19053), a separation buffer (PBS 1X, 2% FBS and 1 mM EDTA) and EasyEights Magnet (StemCell). CD8+ T cells enriched from PBMC were resuspended in the tetramer mix in 50 µL Brilliant Stain Buffer (BSB, BD; ref: 566349), incubated for 20 minutes at room temperature in the dark, washed with PBS 1X containing 0.4 g/L human albumin, and resuspended in the antibody mix in 50 µL Brilliant Stain Buffer. Enriched CD8+ T cells were stained with a viability stain (SYTOX Blue, Invitrogen S34857), the respective tetramer mix and T cell phenotypic markers CD3 BUV737 (BD 12751), CD8 BUV396 (BD 563795), CD45RA FITC (BD 555488), CCR7 BV421 (Biolegend 353208), CD27 PC7 (BD 560609), PD-1 APC-CY7 (Biolegend 329922) and CD39 Percp-ef710 (eBioscience 46-0399-42).
For sorting of Tetramer+ cells, barcoded tetramers were used. Thawed PBMCs underwent the same CD8+ enrichment process as for tetramer analysis, excluding DNase treatment to prevent DNA barcode digestion. Tetramers and antibody staining were performed using the usual protocol.

Instrument

Samples were acquired on a BD Fortessa cytometer. For sorting of tetramer+ cells, a BD ARIA Fusion was used with the same antibody panel

Software

Data was analyzed using FlowJo v10.10.0.

Cell population abundance

Purity was not specifically analyzed as the aim of the enrichment was to increase the total number of CD8+ T cells for analysis. Tetramer+ cells were analyzed within enriched CD8+ T cells and reported as percentage of their parent population.

Gating strategy

Singe cells were gated based on SSC-A vs SSC-W. Lymphocytes were gated within single cells based on SSC-A vs FSC-A. Live cells were gated within lymphocytes based on SYTOX Blue vs FSC-A as cells negative for SYTOX Blue viability stain. CD8 T cells were gated within live cells based on CD3 BUV737 vs CD8 BUV396 as cells positive for both CD3 and CD8. Within CD8 T cells, each tetramer was gated independently based on CD3 vs Tetramer label (PE, Brilliant Violet 711, Brilliant Violet 605, Brilliant Violet 650, PE-CF594, PE-Cy5 and APC). For each patient series (samples acquired in the same analytical run and used for relative analysis), the same gates were applied, based on the pre-treatment sample.
Tetramer-positive populations were analyzed by combinatorial gating within live CD8+ T cells. For screening of T cell responses at baseline vs D64, where at least 200,000 CD8+ T cells were acquired for each sample, a tetramer response was reported if a distinct population of at least 10 cells positive for both tetramer fluorophores was present. Tetramer responses were defined as de novo if not detected at baseline but detected at D64, amplified if detected at baseline but increased more than four-fold at D64, and stable if detected at baseline and D64 at comparable levels (less than four-fold increase).
Gating strategies for analysis and cell sorting are provided as Supplementary Fig 2 and 7, respectively.

☒ Tick this box to confirm that a figure exemplifying the gating strategy is provided in the Supplementary Information.
