# Supplementary material for: A viral-based individualized neoantigen vaccine as adjuvant treatment in resected head and neck squamous cell carcinoma: a randomized Phase I trial
Source: Nat Commun. 2026 Aug 17;17:9863. doi: 10.1038/s41467-026-76667-1 (PMC13578212; doi:10.1038/s41467-026-76667-1)
Supplement: Supplementary file 4 [file 41467_2026_76667_MOESM7_ESM.pdf]

nature portfolio

Peer Review File

A Viral-based individualized neoantigen vaccine as adjuvant treatment in resected head and neck squamous cell carcinoma: a randomized Phase I trial

Corresponding Author: Professor Christian Ottensmeier

This file contains all reviewer reports in order by version, followed by all author rebuttals in order by version.

Version 0:

Reviewer comments:

Reviewer #1

(Remarks to the Author)
DFS analysis is severely underpowered (3 events in 1 arm), and only one-sided p-value is used, which is misleading. It's also anti-conservative that 1 event from arm A is removed, as per protocol. DFS p-value should be deemphasized due to lack of events. In addition there are some small imbalances in patient characteristics (e.g. higher stage at diagnosis in arm B, although not statistically significant) which makes arms harder to compare.

Note that the trial wasn't powered for any particular hypothesis in phase 1, results of which are presented, so the only strength of this study is in correlative endpoints. However, all such endpoints are presented on arm A. Not clear what valuable information is supplied by arm B.

The conclusions about correlative endpoints highlighted in the abstract lack quantitative details. for example it states "Polyepitopic responses to vaccine neoantigens
35 were detected in the blood of patients from both arms after treatment initiation" but in results ". T cell responses to at least one vaccine neoantigen were
207 detected in 8/13 (61.5%) patients". Even results quoted in Results section seem to be descriptive.

It's unclear why 3 patients in Arm B relapsed but only 2 received TG4050.
why there are only 13 patients in Figure 3? it's stated they have "sufficient PBMC samples" - please comment on the remaining patients.

Reviewer #2

(Remarks to the Author)
This paper describes a randomized phase I trial evaluating TG4050, an individualized neoantigen vaccine delivered via a Modified Vaccinia Ankara (MVA) vector, as adjuvant monotherapy in resected head and neck squamous cell carcinoma (HNSCC). The topic is timely and of high translational relevance. The authors approach to interrogate personalized neoantigen vaccination in the minimal residual disease in randomized design and the effort to interrogate vaccine-induced immune responses using of ex vivo ELISpot assays and peptide–MHC tetramer staining are commendable.

However, several important matters require clarification and, in some cases, tempering of conclusions before the manuscript would be suitable for publication.

• The manuscript lacks information about the origin and nature of the selected neoantigens. From which genes where they derived from? Were recurrent mutations observed across patients? Did any targets correspond to oncogenic drivers? Understanding whether the vaccine targets private passenger mutations versus shared or driver mutations is important for both biological interpretation and broader applicability.

• The manuscript does not adequately reference prior studies reporting immune responses to personalized neoantigen

vaccines in solid tumors. Multiple groups have published neoantigen-targeting approaches using mRNA, synthetic long peptides, and other platforms. Proper contextualization within this growing body of literature is essential, particularly when comparing response and durability across platforms.

• On that subject, prior studies of individualized cancer vaccines using SLPs or mRNA have noted long manufacture times given the personalized manufacture required. Could the authors comment and provide the median time needed for manufacture of the TG4050 vaccine?

• In reviewing Figure 3B, there is apparently a low rate of immune responses to the individual neoantigens included in the vaccine—roughly 10% of the targets included per vaccine. This is quite different than the ~60% reported by Blass et al Cell 2025 in their SLP trial, or the mRNA vaccine trials that have reported a range of frequencies. Can the authors comment on that—was that expected, or why the low rate of immune reactivity?

• Concerns arise regarding the robustness of some reported ex vivo ELISpot responses. Several responses classified as positive appear to be of very low magnitude (e.g., ~10 SFC/10^6 PBMC after background subtraction). For example, patient 1010 is classified as having a stable response whereas patient 2040 is classified as non-responsive, yet the magnitude of responses in Figure 3C appears comparable. Similarly, responses in patients 2025, 2033, 2008, and 20016 that are counted as positive are also of the same low magnitude. Representative tetramer staining or convincing longitudinal kinetics for these patients are also lacking (except for patient 2033). For these low-level responses, raw ELISpot images, and tetramer staining with appropriate negative controls should be shown, to allow readers to assess reproducibility, spot quality and distribution.

• In review of the methods for quantification of Elispot reactivity, this seems not quite standard, per prior established harmonized methods for Elispot quantification (by S. Janetzski)—typically 2-3 SDs over the median of the negative control. Please comment and correct.

• The majority of past published studies of individualized vaccines using SLPs or mRNA have identified ~90% of the included vaccine targets to generate de novo responses, wheras the authors identify a substantive fraction that are amplified or stable, signifying that these stimulate preexisting responses. Please comment. Is this related to antigen selection criteria? Or the vaccine delivery platform?

• Perhaps among the most novel findings of the work was the stimulation of ex vivo CD8 responses and the ability to thereafter phenotype these neoantigen specific CD8 T cells over time. The authors should be commended for this. However, it is very difficult to discern the extent to which CD8 vs CD4 responses were generated (CD4 responses have been the predominant responses following SLP or mRNA vaccines). Please comment. And provide this information.

• Any difference in the magnitude or the breadth of T cell responses that were detected in the up front vaccinated patients vs the ones that were treated at recurrence.

• Clarification is needed regarding MVA peptide pool stimulation experiments: how many peptides were combined per pool, and how were pools structured? Pool size directly affects sensitivity and interpretability of T cell assays in Figure 3.

• It is unclear why A02-Flu control responses in Figure 3F were not included longitudinally for patient 2021, who appear to have one of the strongest neoantigen responses. Inclusion of viral control responses across time would help contextualize the magnitude and durability of vaccine-induced responses relative to recall immunity.

• Although tetramer sorting and transcriptomic profiling strengthen the dataset, it would be important to address direct functional relevance beyond IFN-γ ELISpot. To address this, authors should test direct cytotoxicity or polyfunctional cytokine secretion of tetramer positive cells against target cells after cultures with target cells pulsed with a corresponding peptide or autologous tumors if available. Functional validation would substantially strengthen the claim that these responses are truly cytotoxic and tumor-relevant.

• Arm B patients were under observation and were not administered empty MVA as a control. While ethical considerations may have influenced this decision, the absence of an MVA-only arm complicates interpretation of vector-specific versus neoantigen-specific immune effects and limits conclusions about clinical efficacy. This is especially relevant given the strong immunogenicity of MVA itself, and that recent study showed that unrelated vaccine such as SARS-CoV-2 mRNA can improve immune response to cancer (https://doi.org/10.1038/s41586-025-09655-y). The authors suggest that durable neoantigen responses indicate that anti-vector immunity does not overshadow TG4050-induced responses. However, without a non-MVA comparator or direct assessment of vector-dominant responses over time, this conclusion is not fully supported. Moreover, the argument that strong anti-MVA responses may be beneficial due to tissue residency and durability remains speculative without comparative data. The clinical efficacy claims should be substantially toned down. The recurrence difference (3 relapses in the observation arm versus none in the immediate vaccination arm) is intriguing but based on a very small cohort and limited number of events. The absence of an empty-vector control further complicates this interpretation. The manuscript should avoid language suggesting prevention of recurrence and instead frame the findings as hypothesis-generating.

• T cell persistence following individualized cancer vaccines have been noted by other investigators of this modality (Rojas Nature 2023; Hu Leet & Allesoe Nat Med 2021, among others). This finding should be contextualized.

• The manuscript lacks discussion on the limitations of the study.

Reviewer #3

(Remarks to the Author)
Ottensmeier present nice work demonstrating the potential of neoepitope-specific personalized immunotherapy for advanced HNSCC. This is the first work I have seen that characterizes the use of such a personalized immunotherapy after definitive surgery plus risk-stratified adjuvant treatment treatment of HNSCC in general.

Critiques:

Durability of neoepitope specific responses is among the most important data in this paper and it is very difficult to see the plots in 3f. Revise. The figures in general should be cleaned up to display consistent font type and size and clarity. The inability to clearly read some of the figure data really distracts from this nice work.

Should provide the tetramer sorted T cell single cell RNA and VDJ sequencing results (shown in Figure 4c-f) as a supplemental table of processed data. This is likely to be the dataset most interesting to readers that wish to analyze the data themselves.

TRM-like cells are difficult to detect in the blood. By definition they are depleted in blood because they are tissue resident. It is entirely possible that TRM0like cells that were in tumor can leave tumor and be present in the blood, but I would argue that expression of relatively greater ZNF683 alone is not convincing for a TRM-like phenotype. What is the expression of ITGAE in these cells? TRM cells harboring tumor-specific TCRs tend to hold on to ITGAE expression for a bit after existing tissue (relative to T cells that have not been in in tissue). As you know, CD69 in tissue is another TRM marker typically used, but CD69 drops quickly upon withdraw of TCR signaling once the T cell leaves the tumor.

How does the gene expression profile of these circulating tumor-specific T cells identified in this report in compare to the gene expression profile of circulating tumor-specific T cells in patients with relapsed cancer identified in Yossef, et al. Cancer Cell 2023? Is the profile similar? How is similarity determined? If they are different, is this because these expression profiles shown in Fig. 4e are after neoepitope vaccination and the Yossef, et al. tumor-specific T cells are not associated with treatment? Are the signatures shown in Fig. 4e after treatment? If so, how did the neoepitope-specific T cells in circulation change after treatment compared to before treatment? Is this analysis possible?

This neoepitope vaccination method in general excluded the patients that could benefit the most – early recurrences. If this is because of the time needed to GMP generate the vaccine, this should be listed as a limitation of the approach in general.

Version 1:

Reviewer comments:

Reviewer #1

(Remarks to the Author)
My comments were sufficiently addressed

Reviewer #2

(Remarks to the Author)
Overall, the authors were responsive to the critiques, but the highly descriptive nature of the responses and the lack of further samples to do more feels like a missed opportunity. A few suggestions for further clarification are detailed in the document, with our responses in red.

Reviewer #3

(Remarks to the Author)
The authors have appropriately and completely responded to my concerns.

Open Access This Peer Review File is licensed under a Creative Commons Attribution 4.0 International License, which permits use, sharing, adaptation, distribution and reproduction in any medium or format, as long as you give appropriate credit to the original author(s) and the source, provide a link to the Creative Commons license, and indicate if changes were made.
In cases where reviewers are anonymous, credit should be given to 'Anonymous Referee' and the source.
The images or other third party material in this Peer Review File are included in the article's Creative Commons license, unless indicated otherwise in a credit line to the material. If material is not included in the article's Creative Commons license and your intended use is not permitted by statutory regulation or exceeds the permitted use, you will need to obtain permission directly from the copyright holder.
To view a copy of this license, visit https://creativecommons.org/licenses/by/4.0/

REVIEWER COMMENTS

Reviewer #1 (Remarks to the Author):

1) DFS analysis is severely underpowered (3 events in 1 arm), and only one-sided p-value is used, which is misleading. It's also anti-conservative that 1 event from arm A is removed, as per protocol. DFS p-value should be deemphasized due to lack of events. In addition there are some small imbalances in patient characteristics (e.g. higher stage at diagnosis in arm B, although not statistically significant) which makes arms harder to compare.

We agree with Reviewer #1 that the statistical analysis of the DFS is limited due to the small number of events. We have removed it from Figure 2 and from the text section "Clinical outcomes" and kept the Kaplan-Meier curve as descriptive. We also have modified the description of the efficacy results in the discussion.

Although there was a higher percentage of patients with pathological stage IVB in Arm B (43.8% vs 29.4% in Arm A), other known risk factors such as perineural infiltration were higher in Arm A (76.5% vs 43.8% in Arm B). Overall, we could not identify baseline parameters associated with relapse in our study. The 3 patients whose disease recurred in Arm B had a pathological stage at baseline of I/II, III and IVB, respectively. Therefore, we do not expect that the small imbalances in patient characteristics can explain the difference in recurrence we observe.

We added this information in the text in the section "Clinical outcomes".

2) Note that the trial wasn't powered for any particular hypothesis in phase 1, results of which are presented, so the only strength of this study is in correlative endpoints. However, all such endpoints are presented on arm A. Not clear what valuable information is supplied by arm B.

The aim of the randomized Phase 1 trial was to assess the immunogenicity of TG4050 and to collect preliminary efficacy signals. In Arm A, TG4050 was given immediately after confirmation of complete response whereas in Arm B, TG4050 could be given at recurrence. Immunogenicity of TG4050 was assessed in both cases. Because only two patients were treated at recurrence in Arm B, the amount of data for Arm B is limited and is therefore only described succinctly at the end of the section "Induction of neoantigen-specific T cell responses" and associated Supplementary Figure 3 (S4 in revised version).

Biological samples were not collected in Arm B during the observation phase before relapse, so no further correlative analysis can be performed. We are collecting these samples in the ongoing Phase 2 part of the study and are planning to address more systematically the association between tumor antigen-specific T cell responses (whether natural or vaccine-induced) and relapse.

3) The conclusions about correlative endpoints highlighted in the abstract lack quantitative details. for example it states "Polyepitopic responses to vaccine neoantigens
35 were detected in the blood of patients from both arms after treatment initiation" but in results ". T cell responses to at least one vaccine neoantigen were
207 detected in 8/13 (61.5%) patients". Even results quoted in Results section seem to be descriptive.

The immunogenicity analysis was designed as descriptive. We reported quantitative measures in the text in the section “Induction of neoantigen-specific T cell responses” to describe the frequency, magnitude and breadth of responses, with corresponding data in Figures 3a-3c and Supplementary Figure 1 (Supplementary Table 3 in revised version).

We added the following points to strengthen quantitative aspects:

- in the abstract the sentence "Polyepitopic responses to vaccine neoantigens were detected in the blood of patients from both arms after treatment initiation" was modified to “T cell responses to vaccine neoantigens were detected in 73.3% of patients treated with TG4050 immediately following standard of care adjuvant therapy, with a median of 3 neoantigens per responder.”
- in the text section “Induction of neoantigen-specific T cell responses”, a quantification of the rate of *de novo* responses vs amplified was included
- The raw data for *ex vivo* ELISpot was added in Supplementary Table 3, for tetramer in Supplementary Figure 4 to enable readers to assess other quantitative parameters they may be interested in.

We would be happy to add additional parameters in the main text upon specific request.

4) It's unclear why 3 patients in Arm B relapsed but only 2 received TG4050.

Of the 3 patients who relapsed in Arm B, one patient refused TG4050 treatment. This information was added in the text in section “Patients, study design and feasibility”.

5) Why there are only 13 patients in Figure 3? it's stated they have "sufficient PBMC samples" - please comment on the remaining patients.

PBMC samples were collected at baseline and 64 days after the start of TG4050 treatment for all 19 patients treated in Arm A or Arm B. For all patients, T cell responses were initially measured by ELISpot testing individual neoantigens using a research assay. In parallel, tetramer screening was performed for 16 patients (14 in Arm A and 2 in Arm B).

The ELISpot assay was re-established at an independent, GCP-compliant laboratory. However, for some patients with lower PBMC quantity or quality, all collected samples had been used in the first testing campaign. All 13 patients with sufficient PBMC material left were retested, leading to the data reported in Figure 3a-3c. Overall, when combined with the tetramer data, immunogenicity assessment is presented for 17 of the 19 patients treated. The full dataset is summarized in Supplementary Table 3.

Reviewer #2 (Remarks to the Author):

This paper describes a randomized phase I trial evaluating TG4050, an individualized neoantigen vaccine delivered via a Modified Vaccinia Ankara (MVA) vector, as adjuvant monotherapy in resected head and neck squamous cell carcinoma (HNSCC). The topic is timely and of high translational relevance. The authors approach to interrogate personalized neoantigen vaccination in the minimal residual disease in randomized design and the effort to interrogate vaccine-induced immune responses using of ex vivo ELISpot assays and peptide–MHC tetramer staining are commendable.

However, several important matters require clarification and, in some cases, tempering of conclusions before the manuscript would be suitable for publication.

6) The manuscript lacks information about the origin and nature of the selected neoantigens. From which genes where they derived from? Were recurrent mutations observed across patients? Did any targets correspond to oncogenic drivers? Understanding whether the vaccine targets private passenger mutations versus shared or driver mutations is important for both biological interpretation and broader applicability.

Ranking and selection of the vaccine neoantigens was performed solely based on expression as well as predicted processing, binding presentation and immunogenicity scores. No specific weighting or prioritization was applied based on the origin of the mutation (single nucleotide polymorphism, in-frame insertion, deletion or frameshift) or their biological function (cancer driver vs passenger mutations). As expected for our patient population, we detected a high frequency of mutations in oncogenic drivers, notably TP53. However, the vast majority of vaccine neoantigens arose from private passenger mutations, for which a potential mechanistic link to the disease is not known.

We added in Supplementary Figure 1 and the associated text in “Patients, study design and feasibility” an analysis of shared vaccine elements in the 19 patients treated with TG4050 in Arms A and B. Seven TP53-derived vaccine elements were included in six vaccines. Despite this recurrence at the gene level, each mutation occurred at a distinct TP53 locus and therefore generated unique neoantigens. The

impact of these mutations on the p53 function is not known. Overall, among the 540 vaccine neoantigens analyzed across the 19 patients, none was shared between any two vaccines.

Interestingly, none of the 7 TP53-derived neoantigens were found immunogenic by ELISpot or tetramer.

7) The manuscript does not adequately reference prior studies reporting immune responses to personalized neoantigen vaccines in solid tumors. Multiple groups have published neoantigen-targeting approaches using mRNA, synthetic long peptides, and other platforms. Proper contextualization within this growing body of literature is essential, particularly when comparing response and durability across platforms.

The introduction was modified to contextualize the individualized approaches that were reported before or during our study. The following paragraph was added: “In recent years, several groups have applied this concept for the development of personalized neoantigen vaccines using different platforms in different tumor types and clinical settings. mRNAs (Gainor et al, 2024, Rojas et al, 2023, Lopez et al, 2025, Sahin et al, 2026), adjuvanted synthetic long peptides (Awad et al, 2022, Braun et al, 2024, Blass et al, 2025), DNA (Yarchoan et al, 2024) and viral vectors (D’Alise et al, 2024) encoding tumor-specific neoantigens, often used in combination with immune checkpoints inhibitors as standard of care, were able to induce broad and long-lasting T-cell responses to the encoded neoepitopes. Although these studies were single-armed and not designed for efficacy analysis, encouraging signals were observed in terms of disease progression. A formal clinical proof-of-concept for individualized neoantigen vaccines was obtained in the KEYNOTE-942 study, where the mRNA-based mRNA-4157 in combination with pembrolizumab significantly improved recurrence-free survival compared to pembrolizumab alone in resected high-risk melanoma (Weber et al, 2024).”

Comparison of T cell responses from these studies and ours is further developed in response to point 9 and included in the discussion.

8) On that subject, prior studies of individualized cancer vaccines using SLPs or mRNA have noted long manufacture times given the personalized manufacture required. Could the authors comment and provide the median time needed for manufacture of the TG4050 vaccine?

This study was for us the first requiring GMP production of individualized MVA vectors. The aim was to assess the feasibility of the approach. Due to the design of the clinical trial, manufacturing was not a limiting factor: TG4050 treatment start was set at confirmation of complete response >3 months after completion of adjuvant (chemo)radiotherapy.

In Phase 1, we reached a median of 201.5 days (range 146 to 271 days) between surgery and TG4050 product release. Based on the experience gained, we optimized turnaround time in the Phase 2 part of the trial. Preliminary analysis on 62 Phase 2 vaccine productions indicates that the median turnaround time was reduced to 126 days (range 92 – 224 days), and further improvements are being implemented. As this data remains preliminary and not in the scope of this manuscript, we have not added it in the text at this point.

However, we agree that lead time to vaccine administration is one of the main challenges for implementation of individualized cancer vaccines strategies in general, as early treatment will give the best chances to the patients. Using a viral vector comes with higher manufacturing complexity compared to other platforms. We added this limitation, also suggested by reviewer 3 in point 24, in the discussion.

9) In reviewing Figure 3B, there is apparently a low rate of immune responses to the individual neoantigens included in the vaccine—roughly 10% of the targets included per vaccine. This is quite different than the ~60% reported by Blass et al Cell 2025 in their SLP trial, or the mRNA vaccine trials that have reported a range of frequencies. Can the authors comment on that—was that expected, or why the low rate of immune reactivity?

Comparison of the rate of responses between studies is challenging due to the differences in biological factors and analytical methodologies. To support this discussion, we have prepared a table summarizing key parameters and findings in the studies listed in point 7 (see attached Supporting Table for reviewers - Benchmarking immune responses).

We believe that the main factor influencing the reported fraction of immunogenic neoantigens is the analytical method and criteria used to define responders. We based our ELISpot response definition on distribution-free resampling analysis (see point 10) of *ex vivo* ELISpot without prior expansion step for each individual neoantigen at baseline vs D64, 8 weeks after the start of treatment. We found ~10% of responses to treatment for individual neoantigens, with overall 61% of patients responding and a median of 2 responses per patient. Studies using comparable methodology (Rojas et al, 2023, Lopez et al, 2025, Sahin et al, 2026) report response rate comparable to ours with between 50% et 86% of patients responding to at least one neoantigen, 10 to 16% of immunogenic single neoantigens and a median of 2 responses per patient. Studies reporting higher breadth of response used deconvolution of single reactive epitopes after an *ex vivo* expansion step of 10 - 14 days (Braun et al, 2024, Blass et al, 2025, Gainor et al, 2024), based on multiple post-treatment time-points (Yarchoan et al, 2024), or based on different cytokine secretion assay (Awad et al, 2022). Intra-study comparisons of different analytical methods support methodological bias: in Awad et al, 2022, responses to 55% of single neoepitopes were detected by cytokine secretion assay, but only half of those

could be detected by *ex vivo* ELISpot. In Lopez et al, 2025 and Sahin et al, 2026, both *ex vivo* ELISpot and IVS ELISpot were performed. The response rates after IVS were > 50% but only 16 – 20% without the expansion step. Consistent with method-dependent detection rates, we detected additional responses by tetramer not detected by ELISpot. Therefore, it is likely that as far as comparisons are possible, the responses we report are in a comparable range as those measured in prior studies for different platforms.

We cannot fully exclude that the circulating neoantigen-specific T cells response rate we report is lower than in other studies. Below are potential factors which could play a part:

- The HNSCC patient population presents with a high prevalence of lifestyle factors associated with immunosuppression, such as heavy tobacco and alcohol consumption, poor nutrition or previous treatment with chemotherapy affecting the lymphoid compartment
- HNSCC, like PDAC, RCC or TNBC, has a lower TMB than other indications, such as NSCLC or melanoma. Prediction of neoantigens from a smaller pool of mutations may lead to the selection of neoepitopes with lower prediction scores compared to tumors where the selection pool is higher. Consistent with this, in our data we do see an association between higher TMB and higher number of immunogenic neoepitopes. This data has now been added in Supplementary Figure 3.
- Our study tested TG4050 as monotherapy, whereas many other studies combined personalized vaccine with an ICI, which may increase T cell priming and expansion and thus the odds of detection.
- Circulating T cell responses are reported in all studies. $CD^{+}8$ T cells being present in smaller numbers in the blood than $CD4^{+}$ T cells, CD4-polarized responses as described for SLP may be easier to detect. In addition, different platforms may elicit different levels of tissue-resident immunity, which is difficult to detect but likely more relevant for anti-tumor efficacy

Individualized neoantigen vaccination is still new and with limited matching immune and clinical data available, clear correlates of efficacy are yet to be established. Our results, while still purely descriptive, are in line with what the field expects from T cells with anti-tumor activity. With time, we hope that we and others will be able to generate a body of data to narrow down which characteristics of the target antigens, levels of T-cell responses and/or T-cell subsets are linked to protection and enable more quantitative interpretation of immunomonitoring data.

10) Concerns arise regarding the robustness of some reported ex vivo ELISpot responses. Several responses classified as positive appear to be of very low magnitude (e.g., ~10 SFC/10^6 PBMC after background subtraction). For example,

patient 1010 is classified as having a stable response whereas patient 2040 is classified as non-responsive, yet the magnitude of responses in Figure 3C appears comparable. Similarly, responses in patients 2025, 2033, 2008, and 20016 that are counted as positive are also of the same low magnitude. Representative tetramer staining or convincing longitudinal kinetics for these patients are also lacking (except for patient 2033). For these low-level responses, raw ELISpot images, and tetramer staining with appropriate negative controls should be shown, to allow readers to assess reproducibility, spot quality and distribution.
In review of the methods for quantification of Elispot reactivity, this seems not quite standard, per prior established harmonized methods for Elispot quantification (by S. Janetzski)—typically 2-3 SDs over the median of the negative control. Please comment and correct.

Despite multiple attempts over the years, there is no standard way to define ELISpot responses. In the studies referenced in point 9 above and described in the supporting table, some used a threshold of 2, 2.5 or 3 SD above negative control, a fold change of 2 or 3 fold above negative control, a combination of both, or a statistical distribution-free resampling (DFR) method together or not with a minimum spot number to define responses.

Our statisticians suggested the use of the DFR method as described in Moodie et al, 2010 and used to define neoantigen-specific T cell responses in Rojas et al, 2023, Lopez et al, 2025 and Sahin et al, 2026. The DFR(eq) method uses distribution-free resampling to perform, for each antigen considered, the difference in means for all possible permutations of the antigen and control wells replicates and assesses the likelihood that the distribution of the data across the two conditions could have been obtained by chance.

Unlike methods based on thresholds, the DFR takes into account the distribution of both the negative control and test condition to determine positivity without making assumptions on the distribution of the data. Methods based on SD, on the other hand, implicitly assume that negative controls are normally distributed and that the mean and SD are reliably estimated - assumptions that are often violated in practice, especially with small numbers of replicates.

In the cross-laboratory harmonization initiative described in the Moodie et al reference, the DFR(eq) was found to have one of the best detection rate of all methods tested (75% of true positives) together with an overall false positive rate of 11%. To reduce the reporting of false positives, we performed two independent DFR(eq) tests: one comparing the test peptide and negative control condition, and one longitudinal comparing the background-subtracted values for the test peptide before and after treatment (baseline vs D64).

Our *ex vivo* ELISpot assay had low background (median of 4 SFC ± 1.3 SFC in negative control wells), making it possible to quantify low magnitude signals. Responses below 5 SFC/well after background subtraction were excluded, effectively

bringing the lowest reported response to 17 SFC/10^6 PBMC after background subtraction, and this only if both DFR(eq) test conditions were met.

For discussion purposes only we performed a second analysis of our data with a threshold of 3 SD over negative control. Overall, we detected a higher number of responses than with the DFR(eq) method, but some were based on low number of SFC with high variability between replicates. Therefore, we are confident that the response rate we report with the DFR method is robust. We have added the source ELISpot and tetramer data as Supplementary Table 4 and the tetramer gating strategy and images for all responses as Supplementary Figures 3 and 4, to enable readers to perform their own evaluation of the T cell responses reported.

Nevertheless, we recognize the perspective that statistical significance alone may not capture the biological intensity required for clinical efficacy; however, given the complexities of measuring circulating cells, we prioritized the DFR approach for its reproducibility and superior control over the false discovery rate.

Reference: Moodie Z, Price L, Gouttefangeas C, Mander A, Janetzki S, Löwer M, Welters MJ, Ottensmeier C, van der Burg SH, Britten CM. Response definition criteria for ELISPOT assays revisited. Cancer Immunol Immunother. 2010 Oct;59(10):1489-501. doi: 10.1007/s00262-010-0875-4. Epub 2010 Jun 15. PMID: 20549207; PMCID: PMC2909425.

11) The majority of past published studies of individualized vaccines using SLPs or mRNA have identified ~90% of the included vaccine targets to generate de novo responses, wheras the authors identify a substantive fraction that are amplified or stable, signifying that these stimulate preexisting responses. Please comment. Is this related to antigen selection criteria? Or the vaccine delivery platform?

We considered only *de novo* and amplified responses as responses to treatment. Stable responses, which are above background control but not different between baseline and D64, are difficult to interpret due to potential non-specific effects of the peptides used for stimulation. Because each neoantigen and patient combination is different, it is difficult to exclude such artefacts in the absence of adequate negative control for each condition.

Among neoantigen-specific ELISpot responses to treatment we report, 26 (81.2%) were *de novo* responses and 6 (18.8%) were amplified. Among tetramer responses, 16 (88.9%) were *de novo* responses and 2 (11.1%) were amplified. It is important to note that classification of response as *de novo* or amplified based on the detection or not of immunoreactive T cells at baseline is inherently linked to limit of detection of methods, which can vary significantly from study to study. Taking this into account, we consider that our results are comparable to those of past published studies. This quantification has been added to the text in the section “Induction of neoantigen-specific T cell responses”.

We have evaluated the different steps in neoantigen prediction and delivery and could not identify specific steps in the antigen selection, vector design or ELISpot peptide design that may theoretically explain preferential stimulation of pre-existing T cell responses over new ones.

12) Perhaps among the most novel findings of the work was the stimulation of ex vivo CD8 responses and the ability to thereafter phenotype these neoantigen specific CD8 T cells over time. The authors should be commended for this. However, it is very difficult to discern the extent to which CD8 vs CD4 responses were generated (CD4 responses have been the predominant responses following SLP or mRNA vaccines). Please comment. And provide this information.

We have not evaluated yet the relative contribution of the $CD4^+$ vs $CD8^+$ T cell response and we agree it is an important aspect in understanding the mechanism of action of TG4050. Unfortunately, we do not have sufficient PBMC leftover from the Phase 1 part of the study for a meaningful analysis. We are planning to generate this data in the Phase 2 part of the study.

Based on our tetramer analysis, it is clear that $CD8^+$ T cells are a major component of the response to TG4050. This $CD8^+$ T cell induction is expected from a viral vector and likely more prominent than for SLP vaccines.

13) Any difference in the magnitude or the breadth of T cell responses that were detected in the up front vaccinated patients vs the ones that were treated at recurrence.

Only tetramer analysis was performed for the 2 Arm B patients treated at recurrence. Results are presented in Supplementary Table 3 and Supplementary Figures 4 and 5. No response was detected in the first patient out of 10 tetramers screened. For the second patient, 2 / 33 tetramers were reactive. The peak frequency was 0.04% and 0.42% of tetramer+ cells within $CD8^+$ T cells, respectively, comparable to frequencies detected in Arm A patients (see Figure 3f). Both tetramers remained detectable up to end of treatment at D158 at frequencies of 0.02% and 0.27%, respectively.

A comment about the breadth, magnitude and durability was added to the sentence describing these results in the section “Induction of neoantigen-specific T cell responses”.

14) Clarification is needed regarding MVA peptide pool stimulation experiments: how many peptides were combined per pool, and how were pools structured? Pool size directly affects sensitivity and interpretability of T cell assays in Figure 3.

The peptide pool used to assess T cell responses to MVA by ex vivo ELISpot in this study is a mix of two commercial vaccinia peptide pools: Mabtech 3670-1 (PepPool: Vaccinia (CD8), human) and Mabtech 3635-1 (PepPool: Vaccinia (CD4),

human). They contain 102 defined pan-HLA MHC class I-restricted peptides from 59 vaccinia virus proteins and 56 defined MHC class II-restricted peptides from 34 vaccinia virus proteins, respectively. A more detailed description was added to the Methods section.

The number of peptides in the MVA pool is therefore much higher than the three overlapping 15-mers tested for each neoantigen. However, not all peptides in the MVA pool match the patient's HLA alleles. The aim of the MVA control was not to compare directly the magnitude of response to that of neoantigens, but to confirm the ability of the patients to mount any T cell responses after treatment and exclude general immunosuppression.

15) It is unclear why A02-Flu control responses in Figure 3F were not included longitudinally for patient 2021, who appear to have one of the strongest neoantigen responses. Inclusion of viral control responses across time would help contextualize the magnitude and durability of vaccine-induced responses relative to recall immunity.

The HLA-A*02:01 FLU MP 58-66 tetramer was tested for patient 02-0602021 in the longitudinal study. Flu-specific cells were detected but at very low frequencies throughout the time series. Below are the representative gating from Baseline and full longitudinal quantification:

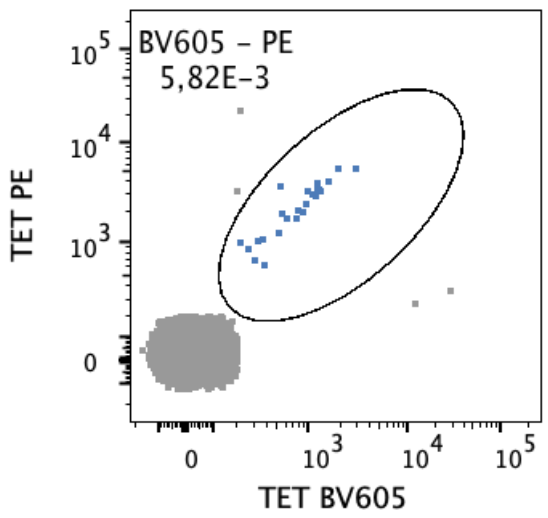

	Singlets/Lymphocytes/ Live lymphocytes/CD8 T cells | Count	Singlets/Lymphocytes/ Live lymphocytes/CD8 T cells/02-0602021_FLU | Freq. of Parent	Singlets/Lymphocytes/ Live lymphocytes/CD8 T cells/02-0602021_FLU | Count
0602021_Baseline_001.fcs	705000	6.67E−3 %	47
0602021_D15_002.fcs	344794	4.93E−3 %	17
0602021_D43_003.fcs	438000	5.94E−3 %	26
0602021_D64_004.fcs	745000	6.04E−3 %	45
0602021_D85_005.fcs	588000	6.46E−3 %	38
0602021_D211_006.fcs	1030000	5.65E−3 %	58
0602021_M18_003.fcs	526000	1.90E−4 %	1
0602021_M24_004.fcs	485000	1.24E−3 %	6

In the longitudinal study, we aligned the overall limit of detection to the samples with the lowest cell numbers. Therefore, only populations with a frequency > 0.01% of $CD8^+$ T cells for at least one time-point were represented in the graphs. The flu-specific responses for this patient did not meet this criterion and were thus not reported.

16) Although tetramer sorting and transcriptomic profiling strengthen the dataset, it would be important to address direct functional relevance beyond IFN-γ ELISpot. To address this, authors should test direct cytotoxicity or polyfunctional cytokine secretion of tetramer positive cells against target cells after cultures with target cells pulsed with a corresponding peptide or autologous tumors if available. Functional validation would substantially strengthen the claim that these responses are truly cytotoxic and tumor-relevant.

As described in the response to point 5, the amount of leftover PBMC available from the Phase 1 part of the trial does not allow performing further immunogenicity assessment. We agree that it is an important point to address and it is one of our priorities for the ongoing Phase 2 part of the trial.

17) Arm B patients were under observation and were not administered empty MVA as a control. While ethical considerations may have influenced this decision, the absence of an MVA-only arm complicates interpretation of vector-specific versus neoantigen-specific immune effects and limits conclusions about clinical efficacy. This is especially relevant given the strong immunogenicity of MVA itself, and that recent study showed that unrelated vaccine such as SARS-CoV-2 mRNA can improve immune response to cancer (https://doi.org/10.1038/s41586-025-09655-y). The authors suggest that durable neoantigen responses indicate that anti-vector immunity does not overshadow TG4050-induced responses. However, without a non-MVA comparator or direct assessment of vector-dominant responses over time, this conclusion is not fully supported. Moreover, the argument that strong anti-MVA responses may be beneficial due to tissue residency and durability remains speculative without comparative data. The clinical efficacy claims should be substantially toned down. The recurrence difference (3 relapses in the observation arm versus none in the immediate vaccination arm) is intriguing but based on a very small cohort and limited number of events. The absence of an empty-vector control further complicates this interpretation. The manuscript should avoid language suggesting prevention of recurrence and instead frame the findings as hypothesis-generating.

We agree, as also responded to Reviewer #1, that the efficacy analysis is limited due to the small number of events and we have toned down the claims.

Our comments regarding anti-vector immunity related to the observation that neoantigen-specific T cell responses could develop even in the presence of MVA-

specific T cell responses, as we found no association (either negative or positive) between the T cell response to MVA antigens and the T cell response to neoantigens (Supplementary Figure 2). One risk when using a viral vector is that anti-vector responses, whether pre-existing of developing with repeated injections, could completely blunt the response to encoded neoantigens. Here, we see that at the epitope level neoantigen-specific $CD8^+$ T cell responses develop with kinetics and magnitude comparable to that of MVA-specific $CD8^+$ T cells in the same patient (Figure 3f), indicating that MVA epitopes are not so overly immunodominant that they completely blunt neoantigen-specific CD8 T cell responses.

The effect of the neoantigen-specific T cell response vs the contribution of the unspecific stimulation induced by the vectors for tumor control is an interesting one and is true for all vaccination platforms. As MVA, LNP/mRNAs and adjuvants included in SLPs activate innate immune pathways which may have a lasting effect in remodeling local and systemic immunity. Unfortunately, dissecting the contribution of vaccine components to anti-tumor efficacy will be difficult to implement in the clinics for all platforms.

Multiple studies have now highlighted the contribution of unspecific cross-immunity conferred by innate immune activation in cancer and infectious diseases protection (the Grippi n et al SARS-CoV-2/ICI study, Lee et al showing BCG confer partial protection against SARS-CoV-2 infection…). Even though the effect of this innate immune memory via epigenetic and metabolic reprogramming of myeloid cells is longer-lasting than the typical systemically detected acute innate immune response to vaccination, it is expected to last in the range of 3-4 months and not years, and to be able to potentiate but not substitute to antigen-specific responses.

The effects of empty vs tumor antigen-encoding viral vectors on tumor control were tested in preclinical studies for different platforms. While empty vectors could have a small and transitory effect on tumor growth, tumor-encoding vectors were necessary for long-term control of tumor growth. We observed this with MVA in EG.7-OVA, TC1 and other mouse models (unpublished data). This was also observed with other viral vectors, such as arenaviruses in the TC1 model (Bonilla et al, Cell Reports, 2021) or AAV in OVA model (Franke et al, Molecular Therapy Advances, 2023). Together, we believe that as for all vaccine platforms, vector components will have multifaceted immune-stimulating activities, but that neoantigen-encoding vaccines will be the most effective at ensuring long-term tumor control.

References: Bonilla WV, Kirchhammer N, Marx AF, Kallert SM, Krzyzaniak MA, Lu M, Darbre S, Schmidt S, Raguz J, Berka U, Vincenti I, Pauzuolis M, Kerber R, Hoepner S, Günther S, Magnus C, Merkler D, Orlinger KK, Zippelius A, Pinschewer DD. Heterologous arenavirus vector prime-boost overrules self-tolerance for efficient tumor-specific CD8 T cell attack. Cell Rep Med. 2021 Mar 3;2(3):100209. doi: 10.1016/j.xcrm.2021.100209. PMID: 33763654; PMCID: PMC7974551.

Franke AC, Hardet R, Prager L, Bentler M, Demeules M, John-Neek P, Jäschke NM, Ha TC, Hacker UT, Adriouch S, Büning H. Capsid-modified adeno-associated virus vectors as novel vaccine platform for cancer immunotherapy. Mol Ther Methods Clin Dev. 2023 Mar 21;29:238-253. doi: 10.1016/j.omtm.2023.03.010. PMID: 37090479; PMCID: PMC10120303.

18) T cell persistence following individualized cancer vaccines have been noted by other investigators of this modality (Rojas Nature 2023; Hu Leet & Allesoe Nat Med 2021, among others). This finding should be contextualized.

This was included in the paragraph added to the introduction in response to point 7.

19) The manuscript lacks discussion on the limitations of the study.
We have reworded the discussion to include more specific references to:

- The limitations of the study related to sample size
- The absence of established correlate of protection to link immune responses and efficacy
- The limitations of the individualized vaccine approach, and specifically those based on viral vectors in terms of manufacturing time
- The limitations of individualized vaccination monotherapy in the context of established tumors

Reviewer #3 (Remarks to the Author):

Ottensmeier present nice work demonstrating the potential of neoepitope-specific personalized immunotherapy for advanced HNSCC. This is the first work I have seen that characterizes the use of such a personalized immunotherapy after definitive surgery plus risk-stratified adjuvant treatment treatment of HNSCC in general.

Critiques:

20) Durability of neoepitope specific responses is among the most important data in this paper and it is very difficult to see the plots in 3f. Revise. The figures in general should be cleaned up to display consistent font type and size and clarity. The inability to clearly read some of the figure data really distracts from this nice work.

All figures have been reformatted to increase legibility. To achieve this, patient identifiers had to be shortened throughout the manuscript.

21) Should provide the tetramer sorted T cell single cell RNA and VDJ sequencing results (shown in Figure 4c-f) as a supplemental table of processed data. This is likely to be the dataset most interesting to readers that wish to analyze the data themselves.

A table containing clonotype IDs, specificity, CDR3A and CDR3B sequences for all cells analyzed in scRNAseq and cell count has been added as Supplementary Table 6.

In addition, the full scRNAseq data was uploaded to GEO under the accession GSE327189 (https://www.ncbi.nlm.nih.gov/geo/query/acc.cgi?acc=GSE327189, token: edifsiygbtkjraj)

22) TRM-like cells are difficult to detect in the blood. By definition they are depleted in blood because they are tissue resident. It is entirely possible that TRM0like cells that were in tumor can leave tumor and be present in the blood, but I would argue that expression of relatively greater ZNF683 alone is not convincing for a TRM-like phenotype. What is the expression of ITGAE in these cells? TRM cells harboring tumor-specific TCRs tend to hold on to ITGAE expression for a bit after existing tissue (relative to T cells that have not been in in tissue). As you know, CD69 in tissue is another TRM marker typically used, but CD69 drops quickly upon withdraw of TCR signaling once the T cell leaves the tumor.

Our scRNAseq analysis compares blood $CD8^+$ T cells with different antigen specificities. Overall, all subsets were relatively close to each other and few markers were differentiating. The combination of markers differentiating MVA/neoantigen-specific cells from CMV and flu-specific cells was ZNF683 combined with GZMB, PRF1, GNLY, TGFB1 and SELL, together with low expression of CCR7 and TCF7. ZNF683 and TGFB1 being often reported as drivers of Trm phenotype, we termed these clusters “Trm-like”, understanding that they do not recapitulate all features of true tissue-resident cells but express more Trm markers relative to the other blood $CD8^+$ T cells in our dataset. The definition of “Trm-like” was also used in Sethna et al, 2025, to describe the signature the signature of MALAT1, GNLY, KLF2, GZMB, ZNF683, GZMM, GZMH, CXCR4, NKG7 and TGFB1 when evaluating blood T cell responses to individualized mRNA neoantigen vaccines. Therefore, we propose to keep the term “Trm-like” but added clarification in the text of the manuscript to highlight the relative nature of this definition.

The markers ITGAE and CD69 (now added to Figure 4e) do not discriminate clusters. ITGAE expression is low in all clusters and CD69 is detected at comparable levels in clusters containing $CD8^+$ T cells with different antigen specificity. Nonetheless, our analysis window is in patients having undergone definitive surgery before disease recurrence. In this context, neoantigen-specific T cells may have not recently encountered their target antigen even if patrolling in tissues, so it is unclear whether CD69 expression could be expected.

Reference: Sethna Z, Guasp P, Reiche C, Milighetti M, Ceglia N, Patterson E, Lihm J, Payne G, Lyudovyk O, Rojas LA, Pang N, Ohmoto A, Amisaki M, Zebboudj A, Odgerel Z, Bruno EM, Zhang SL, Cheng C, Elhanati Y, Derhovanessian E, Manning L, Müller F, Rhee I, Yadav M, Merghoub T, Wolchok JD, Basturk O, Gönen M, Epstein

AS, Momtaz P, Park W, Sugarman R, Varghese AM, Won E, Desai A, Wei AC, D'Angelica MI, Kingham TP, Soares KC, Jarnagin WR, Drebin J, O'Reilly EM, Mellman I, Sahin U, Türeci Ö, Greenbaum BD, Balachandran VP. RNA neoantigen vaccines prime long-lived CD8⁺ T cells in pancreatic cancer. Nature. 2025 Mar;639(8056):1042-1051. doi: 10.1038/s41586-024-08508-4. Epub 2025 Feb 19. PMID: 39972124; PMCID: PMC11946889.

23) How does the gene expression profile of these circulating tumor-specific T cells identified in this report in compare to the gene expression profile of circulating tumor-specific T cells in patients with relapsed cancer identified in Yossef, et al. Cancer Cell 2023? Is the profile similar? How is similarity determined? If they are different, is this because these expression profiles shown in Fig. 4e are after neoepitope vaccination and the Yossef, et al. tumor-specific T cells are not associated with treatment? Are the signatures shown in Fig. 4e after treatment? If so, how did the neoepitope-specific T cells in circulation change after treatment compared to before treatment? Is this analysis possible?

To answer this point, we assessed the overlap between genes differentially expressed when comparing NeoTCRPBL $CD8^+$ T cells and virus-specific $CD8^+$ T cells (CMV, EBV, flu) in Yossef et al, and when comparing post-TG4050 neoantigen-specific $CD8^+$ T cells and virus-specific $CD8^+$ T cells (CMV, flu, MVA) in our study. This analysis is now added as Supplementary Table 5. Of the 476 transcripts positively enriched in NeoTCRPBL, 197 (41.4%) were also enriched in post-TG4050 neoantigen-specific $CD8^+$ T cells, including 3 of the 10 top genes constituting the signature (PASK, S100A4, ITGB1). The overlapping transcripts mapped to metabolic functions such as oxidative phosphorylation and T cell activation. Both DEG lists contained different sets of markers of tissue residency (ITGAE for NeoTCRPBL and ZNF683 for post-TG4050) and antigen experience (TIGIT for NeoTCRPBL and ENTPD1/CD39, TNFRSF9/4-1-BB, HAVCR2/TIM3 and CTLA4 for post-TG4050). Whether this reflects biological differences linked to differences in time and location of priming and antigen exposure, or technical differences, is unknown. Interestingly, the enrichment of SELL (CD62L), a canonical marker of stemness and memory normally not expected in an effector T cell subset, was detected in both NeoTCRPBL and TG4050-associated neoantigen-specific $CD8^+$ T cells.

To gain insight into potential biological differences between NeoTCRPBL and post-TG4050 $CD8^+$ T cells, we focused on transcripts that were regulated in opposite directions in both comparisons. A subset of 43 transcripts was enriched in Yossef's NeoTCRPBL in comparison to viral $CD8^+$ T cells but decreased in TG4050-associated $CD8^+$ T cells compared to viral $CD8^+$ T cells. Of those, several were associated with IFN-mediated responses (BST2, MX1, IFITM2, B2M), MHC-II antigen processing and presentation (HLA-DRB1, HLA-DRB5, HLA-DPA1, HLA-DPB1, HLA-DQA2, HLA-DQB1, CD74) and chronic antigen stimulation (JUNB, TIGIT). On the other hand, 61 transcripts were enriched in TG4050-associated $CD8^+$ T cells

compared to viral $CD8^+$ T cells but decreased in NeoTCRPBL in comparison to viral $CD8^+$ T cells. These included several markers of cytotoxic effector functions (NKG7, GZMH, GZMB, PRF1, FGFBP2). Together, this may indicate that the NeoTCRPBL $CD8^+$ T cells represent a cell subset in a state of chronic stimulation and inflammation, less functional than the TG4050-associated $CD8^+$ T cells. To formally demonstrate that this difference is due to the effect of the vectored vaccine, comparison of scRNAseq profiles of neoantigen-specific $CD8^+$ T cells pre-existing TG4050 treatment and their matching counterpart would be necessary. It is not currently possible to obtain meaningful data due to the small number of $tetramer^+$ cells at baseline for neoantigen-specific cells, but we will continue to investigate and hope to perform this analysis in the Phase 2 part of the trial.

24) This neoepitope vaccination method in general excluded the patients that could benefit the most – early recurrences. If this is because of the time needed to GMP generate the vaccine, this should be listed as a limitation of the approach in general.

This paragraph has been added to the discussion:

Manufacturing timelines remain a significant challenge to maximizing patient benefit. In this study, TG4050 was administered three months after completion of primary adjuvant treatment, a cut-off selected because patients are typically considered clinically disease-free at this time point. However, 15 of the 76 patients who underwent surgery (19.7%) experienced relapse before this cut-off and were therefore unable to receive TG4050. While ongoing improvements in manufacturing may shorten production timelines to under 100 days and allow earlier post-surgical administration, very early relapses occurring shortly after surgery would still preclude treatment during the optimal tumor-free window.

Reviewer #2 (Remarks to the Author):

This paper describes a randomized phase I trial evaluating TG4050, an individualized neoantigen vaccine delivered via a Modified Vaccinia Ankara (MVA) vector, as adjuvant monotherapy in resected head and neck squamous cell carcinoma (HNSCC). The topic is timely and of high translational relevance. The authors approach to interrogate personalized neoantigen vaccination in the minimal residual disease in randomized design and the effort to interrogate vaccine-induced immune responses using of ex vivo ELISpot assays and peptide–MHC tetramer staining are commendable. However, several important matters require clarification and, in some cases, tempering of conclusions before the manuscript would be suitable for publication. 6) The manuscript lacks information about the origin and nature of the selected neoantigens. From which genes where they derived from? Were recurrent mutations observed across patients? Did any targets correspond to oncogenic drivers? Understanding whether the vaccine targets private passenger mutations versus shared or driver mutations is important for both biological interpretation and broader applicability.

Authors: Ranking and selection of the vaccine neoantigens was performed solely based on expression as well as predicted processing, binding presentation and immunogenicity scores. No specific weighting or prioritization was applied based on the origin of the mutation (single nucleotide polymorphism, in-frame insertion, deletion or frameshift) or their biological function (cancer driver vs passenger mutations). As expected for our patient population, we detected a high frequency of mutations in oncogenic drivers, notably TP53. However, the vast majority of vaccine neoantigens arose from private passenger mutations, for which a potential mechanistic link to the disease is not known.

We added in Supplementary Figure 1 and the associated text in “Patients, study design and feasibility” an analysis of shared vaccine elements in the 19 patients treated with TG4050 in Arms A and B. Seven TP53-derived vaccine elements were included in six vaccines. Despite this recurrence at the gene level, each mutation occurred at a distinct TP53 locus and therefore generated unique neoantigens. The impact of these mutations on the p53 function is not known. Overall, among the 540 vaccine neoantigens analyzed across the 19 patients, none was shared between any two vaccines.

Interestingly, none of the 7 TP53-derived neoantigens were found immunogenic by ELISpot or tetramer.

The authors now clarify that neoantigen prioritization was agnostic to biological function and largely driven by predicted immunogenicity and expression. The observation that most vaccine neoantigens derive from private passenger mutations is expected and useful information. The additional analysis showing no shared neoantigens across patients is also valuable. However, the response remains somewhat descriptive and misses an opportunity for deeper biological interpretation.

The manuscript still lacks analysis of whether immunogenic neoantigens differed from non-immunogenic ones in terms of clonality, expression, predicted affinity, or mutational class. Authors should provide a table with all targeted neoantigens per patient, or, at least, immunogenic neoantigens per patient.

Authors: The sequences of all neoantigens tested by ELISpot and those reactive by tetramer were added to Supplementary Table 4 and Supplementary Fig 3, respectively.

A new analysis of the association between prediction score and measured immunogenicity was added in Supplementary Fig 5, with the following text in the main manuscript: *We examined whether predicted antigen presentation, mutant allele expression, binding affinity or the composite immunogenicity score used for epitope selection, which integrates expression, presentation likelihood, binding affinity and predicted T-cell repertoire recognition, were associated with experimentally detected immunogenicity by ELISpot, tetramer, or both assays (Supplementary Fig 5). Although HLA binding affinity score showed a positive association with responses detected by both assays (Supplementary Fig 5b), only composite immunogenicity scores significantly associated with responses detected by ELISpot and/or tetramer staining (Supplementary Fig 5d).*

7) The manuscript does not adequately reference prior studies reporting immune responses to personalized neoantigen vaccines in solid tumors. Multiple groups have published neoantigen-targeting approaches using mRNA, synthetic long peptides, and other platforms. Proper contextualization within this growing body of literature is essential, particularly when comparing response and durability across platforms.

Authors: The introduction was modified to contextualize the individualized approaches that were reported before or during our study. The following paragraph was added: “In recent years, several groups have applied this concept for the development of personalized neoantigen vaccines using different platforms in different tumor types and clinical settings. mRNAs (Gainor et al, 2024, Rojas et al, 2023, Lopez et al, 2025, Sahin et al, 2026), adjuvanted synthetic long peptides (Awad et al, 2022, Braun et al, 2024, Blass et al, 2025), DNA (Yarchoan et al, 2024) and viral vectors (D’Alise et al, 2024) encoding tumor-specific neoantigens, often used in combination with immune checkpoints inhibitors as standard of care, were able to induce broad and long-lasting T-cell responses to the encoded neoepitopes. Although these studies were single-armed and not designed for efficacy analysis, encouraging signals were observed in terms of disease progression. A formal clinical proof-of-concept for individualized neoantigen vaccines was obtained in the KEYNOTE-942 study, where the mRNA-based mRNA-4157 in combination with pembrolizumab significantly improved recurrence-free survival compared to pembrolizumab alone in resected high-risk melanoma (Weber et al, 2024).”

Comparison of T cell responses from these studies and ours is further developed in response to point 9 and included in the discussion.

This concern has been satisfactorily addressed.

8) On that subject, prior studies of individualized cancer vaccines using SLPs or mRNA have noted long manufacture times given the personalized manufacture required. Could the authors comment and provide the median time needed for manufacture of the TG4050 vaccine?

Authors: This study was for us the first requiring GMP production of individualized MVA vectors. The aim was to assess the feasibility of the approach. Due to the design of the clinical trial, manufacturing was not a limiting factor: TG4050 treatment start was set at confirmation of complete response >3 months after completion of adjuvant (chemo)radiotherapy.

In Phase 1, we reached a median of 201.5 days (range 146 to 271 days) between surgery and TG4050 product release. Based on the experience gained, we optimized turnaround time in the Phase 2 part of the trial. Preliminary analysis on 62 Phase 2 vaccine productions indicates that the median turnaround time was reduced to 126 days (range 92 – 224 days), and further improvements are being implemented. As this data remains preliminary and not in the scope of this manuscript, we have not added it in the text at this point.

However, we agree that lead time to vaccine administration is one of the main challenges for implementation of individualized cancer vaccines strategies in general, as early treatment will give the best chances to the patients. Using a viral vector comes with higher manufacturing complexity compared to other platforms. We added this limitation, also suggested by reviewer 3 in point 24, in the discussion.

This concern has been satisfactorily addressed.

9) In reviewing Figure 3B, there is apparently a low rate of immune responses to the individual neoantigens included in the vaccine—roughly 10% of the targets included per vaccine. This is quite different than the ~60% reported by Blass et al Cell 2025 in their SLP trial, or the mRNA vaccine trials that have reported a range of frequencies. Can the authors comment on that—was that expected, or why the low rate of immune reactivity?

Authors: Comparison of the rate of responses between studies is challenging due to the differences in biological factors and analytical methodologies. To support this discussion, we have prepared a table summarizing key parameters and findings in the studies listed in point 7 (see attached Supporting Table for reviewers - Benchmarking immune responses).

We believe that the main factor influencing the reported fraction of immunogenic neoantigens is the analytical method and criteria used to define responders. We based our ELISpot response definition on distribution-free resampling analysis (see

point 10) of *ex vivo* ELISpot without prior expansion step for each individual neoantigen at baseline vs D64, 8 weeks after the start of treatment. We found ~10% of responses to treatment for individual neoantigens, with overall 61% of patients responding and a median of 2 responses per patient. Studies using comparable methodology (Rojas et al, 2023, Lopez et al, 2025, Sahin et al, 2026) report response rate comparable to ours with between 50% et 86% of patients responding to at least one neoantigen, 10 to 16% of immunogenic single neoantigens and a median of 2 responses per patient. Studies reporting higher breadth of response used deconvolution of single reactive epitopes after an *ex vivo* expansion step of 10 - 14 days (Braun et al, 2024, Blass et al, 2025, Gainor et al, 2024), based on multiple post-treatment time-points (Yarchoan et al, 2024), or based on different cytokine secretion assay (Awad et al, 2022). Intra-study comparisons of different analytical methods support methodological bias: in Awad et al, 2022, responses to 55% of single neoepitopes were detected by cytokine secretion assay, but only half of those could be detected by *ex vivo* ELISpot. In Lopez et al, 2025 and Sahin et al, 2026, both *ex vivo* ELISpot and IVS ELISpot were performed. The response rates after IVS were > 50% but only 16 – 20% without the expansion step. Consistent with method-dependent detection rates, we detected additional responses by tetramer not detected by ELISpot. Therefore, it is likely that as far as comparisons are possible, the responses we report are in a comparable range as those measured in prior studies for different platforms.

We cannot fully exclude that the circulating neoantigen-specific T cells response rate we report is lower than in other studies. Below are potential factors which could play a part:

- The HNSCC patient population presents with a high prevalence of lifestyle factors associated with immunosuppression, such as heavy tobacco and alcohol consumption, poor nutrition or previous treatment with chemotherapy affecting the lymphoid compartment

- HNSCC, like PDAC, RCC or TNBC, has a lower TMB than other indications, such as NSCLC or melanoma. Prediction of neoantigens from a smaller pool of mutations may lead to the selection of neoepitopes with lower prediction scores compared to tumors where the selection pool is higher. Consistent with this, in our data we do see an association between higher TMB and higher number of immunogenic neoepitopes. This data has now been added in Supplementary Figure 3.

- Our study tested TG4050 as monotherapy, whereas many other studies combined personalized vaccine with an ICI, which may increase T cell priming and expansion and thus the odds of detection.

- Circulating T cell responses are reported in all studies. CD8+ T cells being present in smaller numbers in the blood than CD4+ T cells, CD4-polarized responses as described for SLP may be easier to detect. In addition, different platforms may elicit

different levels of tissue-resident immunity, which is difficult to detect but likely more relevant for anti-tumor efficacy

Individualized neoantigen vaccination is still new and with limited matching immune and clinical data available, clear correlates of efficacy are yet to be established. Our results, while still purely descriptive, are in line with what the field expects from T cells with anti-tumor activity. With time, we hope that we and others will be able to generate a body of data to narrow down which characteristics of the target antigens, levels of T-cell responses and/or T-cell subsets are linked to protection and enable more quantitative interpretation of immunomonitoring data. While ex vivo assays are more stringent compared to expansion assays, the extremely low magnitude of some responses remains difficult to interpret functionally.

We appreciate a detailed and technically informed explanation for the relatively low apparent response rate. Benchmarking against comparable studies using similar methodologies is helpful. It is notable that the studies that used comparable ex vivo methodology (Rojas et al, 2023, Lopez et al, 2025, Sahin et al, 2026) had lover number of neoantigens in their vaccine (20 vs up to 30 in this study), and used more stringent response definition criteria with distribution free resampling DMSO control and ≥7 SFU (compared to 5 in this study). Overall, the response is thoughtful but still does not fully resolve concerns regarding the relatively modest breadth of detectable responses.

Authors: The justification of the 5 SFC threshold is developed in the next question. We do agree that even taking into account the limitations of quantitative cross-study comparisons, we report relatively modest levels of circulating T cell responses with the ELISpot methodology. ELISpot is a relatively insensitive assay, and indeed several CD8+ T cell responses detected by tetramer in our study were under the detection limit of ELISpot. Therefore, ELISpot likely underestimates the total number of responses, in our study as in others, and misses low level T cell responses which may still have biological activity.

The magnitude of circulating T cell responses has been shown to be a generally poor predictor of anti-tumor activity. Other factors such as functionality and localization are critical for clinical benefit. This is why we do not see the ELISpot response levels as a concern. Accumulation of a large number of clinical and translational data, in the randomized phase 2 and beyond, will help refine correlates and thresholds of protection for neoantigen-specific T cell responses.

10) Concerns arise regarding the robustness of some reported ex vivo ELISpot responses. Several responses classified as positive appear to be of very low magnitude (e.g., ~10 SFC/10^6 PBMC after background subtraction). For example, patient 1010 is classified as having a stable response whereas patient 2040 is classified as non-responsive, yet the magnitude of responses in Figure 3C appears comparable. Similarly, responses in patients 2025, 2033, 2008, and 20016 that are counted as positive are also of the same low magnitude. Representative tetramer

staining or convincing longitudinal kinetics for these patients are also lacking (except for patient 2033). For these low-level responses, raw ELISpot images, and tetramer staining with appropriate negative controls should be shown, to allow readers to assess reproducibility, spot quality and distribution.

In review of the methods for quantification of Elispot reactivity, this seems not quite standard, per prior established harmonized methods for Elispot quantification (by S. Janetzski)—typically 2-3 SDs over the median of the negative control. Please comment and correct.

Authors: Despite multiple attempts over the years, there is no standard way to define ELISpot responses. In the studies referenced in point 9 above and described in the supporting table, some used a threshold of 2, 2.5 or 3 SD above negative control, a fold change of 2 or 3 fold above negative control, a combination of both, or a statistical distribution-free resampling (DFR) method together or not with a minimum spot number to define responses.

Our statisticians suggested the use of the DFR method as described in Moodie et al, 2010 and used to define neoantigen-specific T cell responses in Rojas et al, 2023, Lopez et al, 2025 and Sahin et al, 2026. The DFR(eq) method uses distribution-free resampling to perform, for each antigen considered, the difference in means for all possible permutations of the antigen and control wells replicates and assesses the likelihood that the distribution of the data across the two conditions could have been obtained by chance.

Unlike methods based on thresholds, the DFR takes into account the distribution of both the negative control and test condition to determine positivity without making assumptions on the distribution of the data. Methods based on SD, on the other hand, implicitly assume that negative controls are normally distributed and that the mean and SD are reliably estimated - assumptions that are often violated in practice, especially with small numbers of replicates.

In the cross-laboratory harmonization initiative described in the Moodie et al reference, the DFR(eq) was found to have one of the best detection rate of all methods tested (75% of true positives) together with an overall false positive rate of 11%. To reduce the reporting of false positives, we performed two independent DFR(eq) tests: one comparing the test peptide and negative control condition, and one longitudinal comparing the background-subtracted values for the test peptide before and after treatment (baseline vs D64).

Our *ex vivo* ELISpot assay had low background (median of 4 SFC ± 1.3 SFC in negative control wells), making it possible to quantify low magnitude signals. Responses below 5 SFC/well after background subtraction were excluded, effectively bringing the lowest reported response to 17 SFC/10^6 PBMC after background subtraction, and this only if both DFR(eq) test conditions were met.

For discussion purposes only we performed a second analysis of our data with a threshold of 3 SD over negative control. Overall, we detected a higher number of responses than with the DFR(eq) method, but some were based on low number of SFC with high variability between replicates. Therefore, we are confident that the response rate we report with the DFR method is robust. We have added the source

ELISpot and tetramer data as Supplementary Table 4 and the tetramer gating strategy and images for all responses as Supplementary Figures 3 and 4, to enable readers to perform their own evaluation of the T cell responses reported.

Nevertheless, we recognize the perspective that statistical significance alone may not capture the biological intensity required for clinical efficacy; however, given the complexities of measuring circulating cells, we prioritized the DFR approach for its reproducibility and superior control over the false discovery rate.

Reference: Moodie Z, Price L, Gouttefangeas C, Mander A, Janetzki S, Löwer M, Welters MJ, Ottensmeier C, van der Burg SH, Britten CM. Response definition criteria for ELISPOT assays revisited. Cancer Immunol Immunother. 2010 Oct;59(10):1489-501. doi: 10.1007/s00262-010-0875-4. Epub 2010 Jun 15. PMID: 20549207; PMCID: PMC2909425.

The inclusion of gating strategies, and tetramer plots in the supplementary materials is appreciated and substantially improves transparency. We also appreciate the inclusion of the raw ELISpot numbers in Supplementary table 4. However, we still think that raw ELISpot images for these low-level responses and appropriate negative controls should be included. Additionally, Rojas et al, 2023, Lopez et al, 2025 and Sahin et al, 2026 excluded the responses below 7 SFC/well after background subtraction. What was the reason to opt for a threshold of 5 SFC/well in this study?

Authors: The threshold of 5 SFC per well after background subtraction to define a response was based on the measured performance of our assay. DMSO control wells across all patients and samples showed a mean SD of 1.63 SFC between triplicates. We considered that values within 3 SD of the DMSO background could still be arising by chance, and therefore set the minimal response threshold to 4.89, rounded to 5 SFC per well above DMSO control.

11) The majority of past published studies of individualized vaccines using SLPs or mRNA have identified ~90% of the included vaccine targets to generate de novo responses, wheras the authors identify a substantive fraction that are amplified or stable, signifying that these stimulate preexisting responses. Please comment. Is this related to antigen selection criteria? Or the vaccine delivery platform?

Authors: We considered only *de novo* and amplified responses as responses to treatment. Stable responses, which are above background control but not different between baseline and D64, are difficult to interpret due to potential non-specific effects of the peptides used for stimulation. Because each neoantigen and patient

combination is different, it is difficult to exclude such artefacts in the absence of adequate negative control for each condition.

Among neoantigen-specific ELISpot responses to treatment we report, 26 (81.2%) were *de novo* responses and 6 (18.8%) were amplified. Among tetramer responses, 16 (88.9%) were *de novo* responses and 2 (11.1%) were amplified. It is important to note that classification of response as *de novo* or amplified based on the detection or not of immunoreactive T cells at baseline is inherently linked to limit of detection of methods, which can vary significantly from study to study. Taking this into account, we consider that our results are comparable to those of past published studies. This quantification has been added to the text in the section “Induction of neoantigen-specific T cell responses”.

We have evaluated the different steps in neoantigen prediction and delivery and could not identify specific steps in the antigen selection, vector design or ELISpot peptide design that may theoretically explain preferential stimulation of pre-existing T cell responses over new ones.

This response is satisfactory overall. The clarification that only de novo and amplified responses were considered treatment-induced resolves much of the concern. The authors appropriately acknowledge that assay sensitivity strongly influences classification of “de novo” versus “pre-existing” responses.

12) Perhaps among the most novel findings of the work was the stimulation of ex vivo CD8 responses and the ability to thereafter phenotype these neoantigen specific CD8 T cells over time. The authors should be commended for this. However, it is very difficult to discern the extent to which CD8 vs CD4 responses were generated (CD4 responses have been the predominant responses following SLP or mRNA vaccines). Please comment. And provide this information.

Authors: We have not evaluated yet the relative contribution of the CD4+ vs CD8+ T cell response and we agree it is an important aspect in understanding the mechanism of action of TG4050. Unfortunately, we do not have sufficient PBMC leftover from the Phase 1 part of the study for a meaningful analysis. We are planning to generate this data in the Phase 2 part of the study.

Based on our tetramer analysis, it is clear that CD8+ T cells are a major component of the response to TG4050. This CD8+ T cell induction is expected from a viral vector and likely more prominent than for SLP vaccines.

The authors appropriately acknowledge this as a limitation, and the lack of sufficient PBMC material is understandable. We appreciate adding comment on this limitation to the discussion. However, this remains a major unresolved issue.

13) Any difference in the magnitude or the breadth of T cell responses that were detected in the up front vaccinated patients vs the ones that were treated at recurrence.

Authors: Only tetramer analysis was performed for the 2 Arm B patients treated at recurrence. Results are presented in Supplementary Table 3 and Supplementary Figures 4 and 5. No response was detected in the first patient out of 10 tetramers screened. For the second patient, 2 / 33 tetramers were reactive. The peak frequency was 0.04% and 0.42% of tetramer+ cells within CD8+ T cells, respectively, comparable to frequencies detected in Arm A patients (see Figure 3f). Both tetramers remained detectable up to end of treatment at D158 at frequencies of 0.02% and 0.27%, respectively.

A comment about the breadth, magnitude and durability was added to the sentence describing these results in the section “Induction of neoantigen-specific T cell responses”.

This concern has been satisfactorily addressed.

14) Clarification is needed regarding MVA peptide pool stimulation experiments: how many peptides were combined per pool, and how were pools structured? Pool size directly affects sensitivity and interpretability of T cell assays in Figure 3.

Authors: The peptide pool used to assess T cell responses to MVA by ex vivo ELISpot in this study is a mix of two commercial vaccinia peptide pools: Mabtech 3670-1 (PepPool: Vaccinia (CD8), human) and Mabtech 3635-1 (PepPool: Vaccinia (CD4), human). They contain 102 defined pan-HLA MHC class I-restricted peptides from 59 vaccinia virus proteins and 56 defined MHC class II-restricted peptides from 34 vaccinia virus proteins, respectively. A more detailed description was added to the Methods section.

The number of peptides in the MVA pool is therefore much higher than the three overlapping 15-mers tested for each neoantigen. However, not all peptides in the MVA pool match the patient’s HLA alleles. The aim of the MVA control was not to compare directly the magnitude of response to that of neoantigens, but to confirm the ability of the patients to mount any T cell responses after treatment and exclude general immunosuppression.

This concern has been satisfactorily addressed.

15) It is unclear why A02-Flu control responses in Figure 3F were not included longitudinally for patient 2021, who appear to have one of the strongest neoantigen responses. Inclusion of viral control responses across time would help contextualize the magnitude and durability of vaccine-induced responses relative to recall immunity.

Authors: The HLA-A*02:01 FLU MP 58-66 tetramer was tested for patient 02-0602021 in the longitudinal study. Flu-specific cells were detected but at very low

frequencies throughout the time series. Below are the representative gating from Baseline and full longitudinal quantification:

In the longitudinal study, we aligned the overall limit of detection to the samples with the lowest cell numbers. Therefore, only populations with a frequency > 0.01% of CD8+ T cells for at least one time-point were represented in the graphs. The flu-specific responses for this patient did not meet this criterion and were thus not reported.

This concern has been satisfactorily addressed.

16) Although tetramer sorting and transcriptomic profiling strengthen the dataset, it would be important to address direct functional relevance beyond IFN-γ ELISpot. To address this, authors should test direct cytotoxicity or polyfunctional cytokine secretion of tetramer positive cells against target cells after cultures with target cells pulsed with a corresponding peptide or autologous tumors if available. Functional validation would substantially strengthen the claim that these responses are truly cytotoxic and tumor-relevant.

Authors: As described in the response to point 5, the amount of leftover PBMC available from the Phase 1 part of the trial does not allow performing further immunogenicity assessment. We agree that it is an important point to address and it is one of our priorities for the ongoing Phase 2 part of the trial.

While limited PBMC availability is understandable, the absence of direct functional validation remains a significant weakness of the study.

17) Arm B patients were under observation and were not administered empty MVA as a control. While ethical considerations may have influenced this decision, the absence of an MVA-only arm complicates interpretation of vector-specific versus neoantigen-specific immune effects and limits conclusions about clinical efficacy. This is especially relevant given the strong immunogenicity of MVA itself, and that recent study showed that unrelated vaccine such as SARS-CoV-2 mRNA can improve immune response to cancer (https://doi.org/10.1038/s41586-025-09655-y). The authors suggest that durable neoantigen responses indicate that anti-vector immunity does not overshadow TG4050-induced responses. However, without a non-MVA comparator or direct assessment of vector-dominant responses over time, this conclusion is not fully supported. Moreover, the argument that strong anti-MVA responses may be beneficial due to tissue residency and durability remains speculative without comparative data. The clinical efficacy claims should be substantially toned down. The recurrence difference (3 relapses in the observation arm versus none in the immediate vaccination arm) is intriguing but based on a very small cohort and limited number of events. The absence of an empty-vector control further complicates this interpretation. The manuscript should avoid language suggesting prevention of recurrence and instead frame the findings as hypothesis-generating.

Authors: We agree, as also responded to Reviewer #1, that the efficacy analysis is limited due to the small number of events and we have toned down the claims.

Our comments regarding anti-vector immunity related to the observation that neoantigen-specific T cell responses could develop even in the presence of MVA-specific T cell responses, as we found no association (either negative or positive) between the T cell response to MVA antigens and the T cell response to neoantigens (Supplementary Figure 2). One risk when using a viral vector is that anti-vector responses, whether pre-existing of developing with repeated injections, could completely blunt the response to encoded neoantigens. Here, we see that at the epitope level neoantigen-specific CD8+ T cell responses develop with kinetics and magnitude comparable to that of MVA-specific CD8+ T cells in the same patient (Figure 3f), indicating that MVA epitopes are not so overly immunodominant that they completely blunt neoantigen-specific CD8 T cell responses.

The effect of the neoantigen-specific T cell response vs the contribution of the unspecific stimulation induced by the vectors for tumor control is an interesting one and is true for all vaccination platforms. As MVA, LNP/mRNAs and adjuvants included in SLPs activate innate immune pathways which may have a lasting effect in remodeling local and systemic immunity. Unfortunately, dissecting the contribution of vaccine components to anti-tumor efficacy will be difficult to implement in the clinics for all platforms.

Multiple studies have now highlighted the contribution of unspecific cross-immunity conferred by innate immune activation in cancer and infectious diseases protection (the Grippi n et al SARS-CoV-2/ICI study, Lee et al showing BCG confer partial protection against SARS-CoV-2 infection…). Even though the effect of this innate immune memory via epigenetic and metabolic reprogramming of myeloid cells is longer-lasting than the typical systemically detected acute innate immune response to vaccination, it is expected to last in the range of 3-4 months and not years, and to be able to potentiate but not substitute to antigen-specific responses.

The effects of empty vs tumor antigen-encoding viral vectors on tumor control were tested in preclinical studies for different platforms. While empty vectors could have a small and transitory effect on tumor growth, tumor-encoding vectors were necessary for long-term control of tumor growth. We observed this with MVA in EG.7-OVA, TC1 and other mouse models (unpublished data). This was also observed with other viral vectors, such as arenaviruses in the TC1 model (Bonilla et al, Cell Reports, 2021) or AAV in OVA model (Franke et al, Molecular Therapy Advances, 2023). Together, we believe that as for all vaccine platforms, vector components will have multifaceted immune-stimulating activities, but that neoantigen-encoding vaccines will be the most effective at ensuring long-term tumor control.

References: Bonilla WV, Kirchhammer N, Marx AF, Kallert SM, Krzyzaniak MA, Lu M, Darbre S, Schmidt S, Raguz J, Berka U, Vincenti I, Pauzuolis M, Kerber R, Hoepner S, Günther S, Magnus C, Merkler D, Orlinger KK, Zippelius A, Pinschewer DD.

Heterologous arenavirus vector prime-boost overrules self-tolerance for efficient tumor-specific CD8 T cell attack. Cell Rep Med. 2021 Mar 3;2(3):100209. doi: 10.1016/j.xcrm.2021.100209. PMID: 33763654; PMCID: PMC7974551.

Franke AC, Hardet R, Prager L, Bentler M, Demeules M, John-Neek P, Jäschke NM, Ha TC, Hacker UT, Adriouch S, Büning H. Capsid-modified adeno-associated virus vectors as novel vaccine platform for cancer immunotherapy. Mol Ther Methods Clin Dev. 2023 Mar 21;29:238-253. doi: 10.1016/j.omtm.2023.03.010. PMID: 37090479; PMCID: PMC10120303.

Most importantly, the manuscript should remain careful not to imply causality between vaccine-induced neoantigen responses and absence of recurrence. The current dataset supports association and feasibility, not proof of mechanism or clinical efficacy. The authors appropriately toned-down efficacy claims.

18) T cell persistence following individualized cancer vaccines have been noted by other investigators of this modality (Rojas Nature 2023; Hu Leet & Allesoe Nat Med 2021, among others). This finding should be contextualized.

Authors: This was included in the paragraph added to the introduction in response to point 7.

Adequately addressed.

19) The manuscript lacks discussion on the limitations of the study.

Authors: We have reworded the discussion to include more specific references to:

- The limitations of the study related to sample size

- The absence of established correlate of protection to link immune responses and efficacy

- The limitations of the individualized vaccine approach, and specifically those based on viral vectors in terms of manufacturing time

- The limitations of individualized vaccination monotherapy in the context of established tumors

Adequately addressed.

Additional comments from the authors: The DFS analysis at the time of submission was dated from 31 March 2025, corresponding to a median follow-up of 30 months. We have recently performed a new analysis, with a median follow-up of 41 months. There were no additional relapses in either arm. We propose to include this information as one sentence in the text in the efficacy section, without modifying the figures or previously established conclusions.
